# HIC1 (hypermethylated in cancer 1) SUMOylation is dispensable for DNA repair but is essential for the apoptotic DNA damage response (DDR) to irreparable DNA double-strand breaks (DSBs)

**DOI:** 10.18632/oncotarget.13807

**Published:** 2016-12-07

**Authors:** Sonia Paget, Marion Dubuissez, Vanessa Dehennaut, Joe Nassour, Brennan T. Harmon, Nathalie Spruyt, Ingrid Loison, Corinne Abbadie, Brian R. Rood, Dominique Leprince

**Affiliations:** ^1^ University Lille, CNRS, Institut Pasteur de Lille, UMR 8161-M3T-Mechanisms of Tumorigenesis and Targeted Therapies, Lille, France; ^2^ Genomics Core, Children's National Medical Center, Washington DC, USA; ^3^ Center for Cancer and Immunology Research, Children's National Medical Center, Washington DC, USA; ^4^ Present Address: Maisonneuve-Rosemont Hospital Research Center, Maisonneuve-Rosemont Hospital, Boulevard l'Assomption Montreal, Canada; ^5^ Present Address: The Salk Institute for Biological Studies, Molecular and Cell Biology Department, La Jolla, California, USA

**Keywords:** DNA damage response, HIC1, ATM, MTA1, SUMOylation

## Abstract

The tumor suppressor gene *HIC1 (Hypermethylated In Cancer 1)* encodes a transcriptional repressor mediating the p53-dependent apoptotic response to irreparable DNA double-strand breaks (DSBs) through direct transcriptional repression of *SIRT1*. HIC1 is also essential for DSB repair as silencing of endogenous *HIC1* in BJ-hTERT fibroblasts significantly delays DNA repair in functional Comet assays. HIC1 SUMOylation favours its interaction with MTA1, a component of NuRD complexes. In contrast with irreparable DSBs induced by 16-hours of etoposide treatment, we show that repairable DSBs induced by 1 h etoposide treatment do not increase HIC1 SUMOylation or its interaction with MTA1. Furthermore, HIC1 SUMOylation is dispensable for DNA repair since the non-SUMOylatable E316A mutant is as efficient as wt HIC1 in Comet assays. Upon induction of irreparable DSBs, the ATM-mediated increase of HIC1 SUMOylation is independent of its effector kinase Chk2. Moreover, irreparable DSBs strongly increase both the interaction of HIC1 with MTA1 and MTA3 and their binding to the *SIRT1* promoter. To characterize the molecular mechanisms sustained by this increased repression potential, we established global expression profiles of BJ-hTERT fibroblasts transfected with HIC1-siRNA or control siRNA and treated or not with etoposide. We identified 475 genes potentially repressed by HIC1 with cell death and cell cycle as the main cellular functions identified by pathway analysis. Among them, *CXCL12*, *EPHA4*, *TGFβR3* and *TRIB2*, also known as MTA1 target-genes, were validated by qRT-PCR analyses. Thus, our data demonstrate that HIC1 SUMOylation is important for the transcriptional response to non-repairable DSBs but dispensable for DNA repair.

## INTRODUCTION

The genomic integrity of all living organisms is constantly challenged by deleterious attacks due to endogenous or exogenous genotoxic stress. DNA damage and in particular DNA double-strand breaks (DSBs) are highly deleterious since they can be lethal if unrepaired or predispose to oncogenic transformation if misrepaired. To cope with these lesions, cells have developed multiple interacting pathways called the DNA damage response (DDR) that lead either to damage repair or to programmed cell death depending on the extent of the damage [1]. A multi-branched, highly coordinated signaling cascade of Post-Translational Modifications (PTM) allows the effective recruitment, stabilization and retention at DSBs of numerous proteins including sensors, mediators and effectors of the DDR [2]. A major transducer of DNA damage signaling in the case of DSBs is the activation of the PIKKs (Phosphatidylinositol 3 kinase-like protein kinase) ATM or DNA-PKcs proteins. In particular, the apical ATM kinase phosphorylates hundreds of proteins including histones (H2AX), repair factors (BRCA1), its effector kinase (CHK2) or transcription factors such as P53 [3]. Previous studies demonstrated that together with P53, SIRT1 and the tumor suppressor gene *HIC1* (*Hypermethylated in cancer 1*), which is epigenetically silenced by promoter hypermethylation in many types of human cancers [4, 5], plays a critical role in the DNA damage response [6–8]. Indeed, *HIC1* is a direct target-gene of P53 and upon induction of irreparable DSBs, HIC1 regulates the p53-dependant apoptotic DNA damage response [6]. When treated overnight with etoposide, a DSB inducer, wt Murine Embryo Fibroblasts (MEFs) rapidly begin to die whereas *Hic1–/–* MEFs are resistant to apoptosis. Conversely, re-expression of HIC1 in MCF-7 cells through adenoviral infection restores their sensitivity to P53-induced apoptosis [6]. This effect relies mainly on the HIC1-mediated direct transcriptional repression of *SIRT1*, which deacetylates and inactivates P53 allowing cells to by-pass P53 induced apoptosis and survive DNA damage [6]. Recently, we have shown that HIC1 is also a key player in the response to repairable DNA damage. Down-regulation of endogenous *HIC1* expression through RNA interference in normal human fibroblasts treated for 1 hour with Etoposide delays DNA repair, as shown by functional comet assays [8].

*HIC1* encodes a transcriptional repressor containing an N-terminal BTB domain and five C-terminal C_2_H_2_ *Krüppel*-like Zinc fingers [9] We have shown that HIC1 interacts with 4 major co-repressors complexes involved in chromatin remodelling and epigenetic regulation; CtBP, SWI/SNF, NuRD and the *Polycomb* PRC2 complex [9]. In particular, we have demonstrated through yeast two-hybrid screening and various biochemical approaches that HIC1 interacts with the C-terminal region of MTA1, a core component of NuRD, through a SUMOylation consensus motif in the HIC1 central region [10, 11]. SUMOylation is a highly dynamic and labile PTM that plays a key role in the assembly of multi-protein complexes [12]. The HIC1-MTA1 interaction is regulated by two mutually exclusive PTM of Lysine 314, promotion by SUMOylation and inhibition by acetylation [10, 11]. Previously, we demonstrated that irreparable DSBs induced by a 16 h treatment with etoposide result in a specific increase of HIC1 SUMOylation in an ATM-dependant manner [8]. This increase of HIC1 SUMOylation is correlated with an increased interaction of endogenous HIC1 and MTA1 proteins in etoposide treated normal human fibroblasts, thereby favouring the recruitment of NuRD repressive complexes onto HIC1 target genes [8]. This provides the first mechanism by which the transcriptional repression function of HIC1 is activated upon DNA damage.

In this study, we further investigated the function and regulation of HIC1 SUMOylation during the DNA damage response to repairable and non-repairable DSBs. First, we demonstrate that HIC1 SUMOylation does not increase upon induction of repairable DSBs by a 1 h etoposide treatment. In addition, results from functional DNA repair assays such as Comet assays using overexpression of wt or non-SUMOylatable (E316A) HIC1 in Cos-7 cells that do no express endogenous HIC1 demonstrated that SUMOylation on Lysine 314 is not implicated in DSB repair. Indeed, the efficiency and kinetics of repair exhibited by the E316A point mutant and wild-type HIC1 are virtually indistinguishable. Furthermore, we show that the increased SUMOylation of HIC1 in the presence of irreparable DSBs induced by a 16 hours etoposide treatment is primarily dependent on ATM which is stabilized and activated on chromatin but independent of its nucleoplasmic effector kinase CHK2. As for the HIC1-MTA1 interaction, we showed that it depends on a non-covalent interaction between SUMOylated HIC1 and the SUMO-interacting motif (SIM) in the C-terminal part of MTA1. Furthermore, we demonstrated that HIC1 also interacts with the related corepressor MTA3 and that irreparable DSBs increase this interaction, as shown for MTA1. By ChIP experiments, we showed that induction of irreparable DSBs results in an increased recruitment of MTA1, MTA3 and also of HIC1 onto HIC1-response elements (HiRE) in the *SIRT1* promoter. To further characterize the molecular mechanisms sustained by this increased repression potential, we established global expression profiles of BJ-hTERT fibroblasts transfected with HIC1-siRNA or control siRNA and treated or not with etoposide. We identified 475 genes potentially repressed by HIC1 with cell death and cell cycle as the main cellular functions identified by pathway analysis. Cross referencing this list with the 1024 MTA1 target genes identified by comparing *wt* MEFs (Murine Embryos Fibroblasts) with *Mta1 –/–* MEFs identified 17 common genes. Among them, *CXCL12*, *EPHA4*, *LPHN2*, TGFβR3 and *TRIB2* were shown to be activated in siHIC1 fibroblasts and to be more repressed in control cells treated with Etoposide to increase HIC1 SUMOylation.

In summary, our results demonstrate that HIC1 SUMOylation is dispensable for DNA repair but is important for the p53-dependent apoptotic transcriptional response to irreparable DSBs, notably through the recruitment of MTA1 or MTA3 containing NuRD repressive complexes to the *SIRT1* promoter and other potential direct target genes.

## RESULTS

### Repairable DNA DSBs do not result in a PIKK-dependant increase of HIC1 SUMOylation and interaction with MTA1

**Figure 1:**
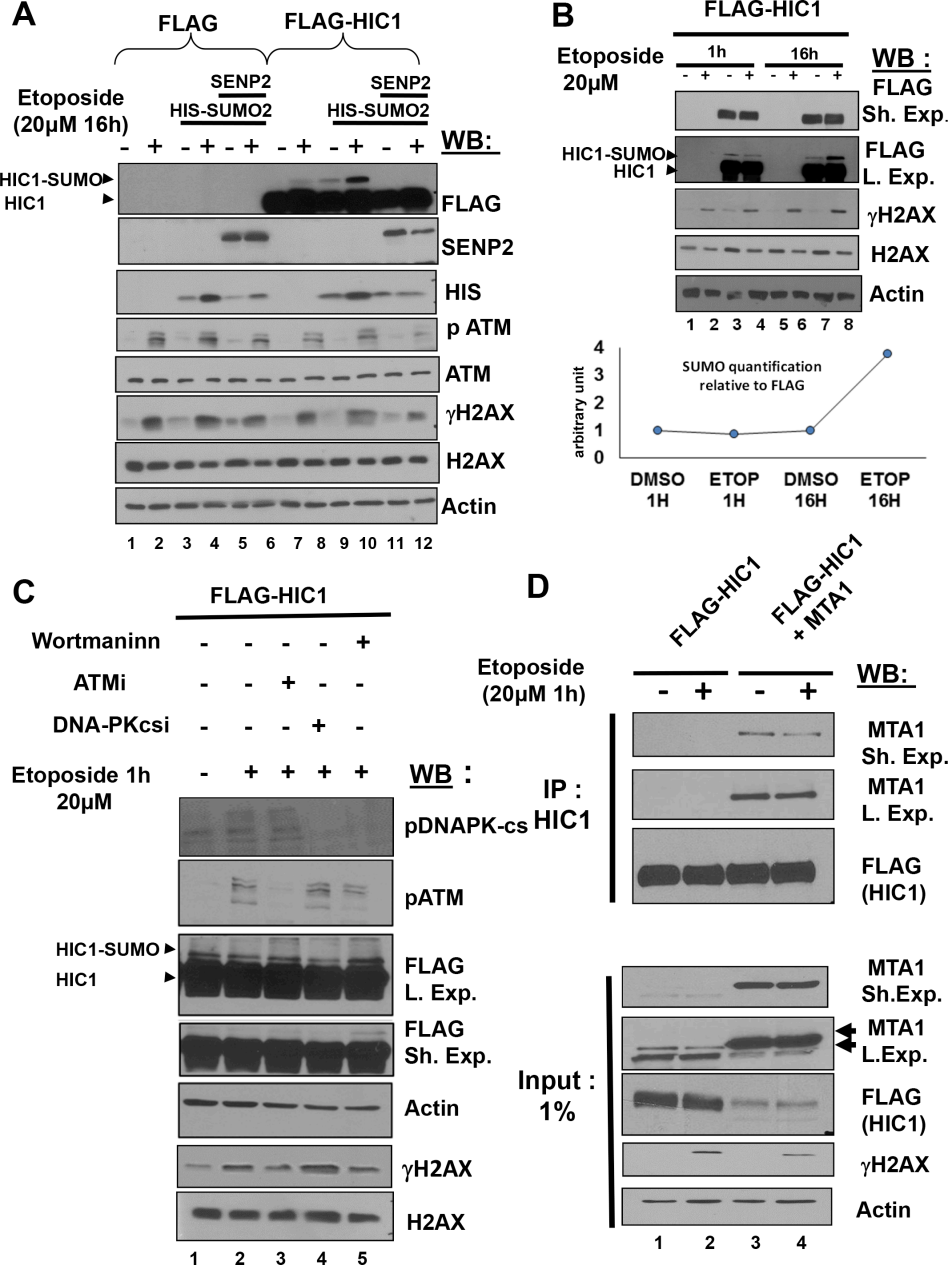
Repairable DNA double-strand breaks (DSBs) induced by a 1 hour etoposide treatment do not lead to an ATM-dependent increase of HIC1 SUMOylation (**A**) Etoposide-induced non-repairable DSBs lead to an increase of HIC1 SUMOylation. HEK293T cells were transfected with the indicated combination of empty FLAG, FLAG-HIC1, SENP2 and SUMO2 expression vectors. 32 hours after transfection cells were incubated for 16 hours with 20 μM etoposide (+) or DMSO (–) as control before direct lysis in denaturing conditions. Total cell extracts were analyzed by Western Blotting (WB) using the indicated antibodies. (**B**) HEK 293T cells were transfected with FLAG-HIC1 and treated with etoposide or DMSO for 1 hour or 16 hours. Cell extracts were prepared as described in panel A) and analyzed by immunoblotting using the indicated antibodies. Quantification of SUMO-HIC1 to total HIC1 (FLAG) was performed with the Fujifilm MultiGauge software (Bottom Panel) (**C**) HEK 293T cells were transfected with FLAG-HIC1 and treated with etoposide or DMSO for 1 hour. Transfected cells were pre-treated or not with the following inhibitors (Wortmannin; ATMi, ATM inhibitor and DNAPKcsi, DNA-PKcs inhibitor) 1 hour before etoposide treatment, as indicated. Cell extracts were prepared as described in panel B) and analyzed by immunoblotting using the indicated antibodies. (**D**) HEK293T cells were co-transfected with the indicated combinations of expression vectors for FLAG-HIC1 and MTA1 and then incubated for 1 hour in etoposide or with DMSO as control. After lysis in IPH buffer, cells extracts were co-immunoprecipitated with anti-HIC1 antibodies. The immunoprecipitates as well as 2% of the whole cell extract (Input) were analyzed by Western blotting with the anti FLAG and anti MTA1 antibody. Note that the MTA1 antibodies detect a doublet of endogenous proteins in non transfected cells whereas the ectopically expressed MTA1 protein co-migrates with the upper band of the doublet (arrowheads).

We previously demonstrated that induction of non-repairable DSBs by overnight (16 h) treatment of transfected HEK293T cells with 20 μM etoposide, an inhibitor of topoisomerase II known to induce DSBs, results in a significant increase of HIC1 SUMOylation [8]. Such a prolonged assault leads to the accumulation of non-repairable damage resulting in a p53-dependent apoptotic response [6]. HEK293T cells were transfected with the empty FLAG or FLAG-HIC1 expression vectors with or without an expression vector for His-SUMO2 and/or the de-SUMOylase SENP2. 48 hours after transfection, cells were treated with 20 μM etoposide for 16 hours to induce non-repairable DNA damage and immediately lysed under denaturing conditions. Total extracts were then analyzed by Western blot with anti-FLAG monoclonal antibodies to detect HIC1 and its SUMOylated forms. As shown in Figure 1A, the isoform of higher molecular weight, corresponding to the SUMOylated form of HIC1, disappeared in presence of SENP2 and is significantly increased after 16 h etoposide treatment, as previously shown [8].

Since endogenous HIC1 also activates the kinetics and/or efficiency of DSB repair in BJ-hTERT fibroblasts, we next wanted to address the importance of HIC1 SUMOylation in the repair process [8]. In striking contrast with the ATM-dependent increase of HIC1 SUMOylation observed in HEK293T cells treated for 16 hours with etoposide [8], HIC1 SUMOylation levels do not increase after the induction of repairable DSBs by a short (1 hour) etoposide treatment (Figure 1B, compare lanes 3 and 4 to lanes 7 and 8). This HIC1 SUMOylation is also independent of ATM activation since its level remains constant when cells are pre-incubated for 1 h with the specific ATM inhibitor Ku-55939 prior to the 1 hour etoposide treatment (Supplementary Figure S1A).

DNA-damaging agents that create DSBs activate a DDR primarily relying on the activation of kinases of the PIKKs (Phosphatidylinositol 3 kinase-like protein kinase) family, ATM or DNA-PKcs proteins [13]. After induction of repairable damage, inhibition of ATM and DNA-PKcs by Wortmaninn, a PI3K inhibitor also inhibiting PIKKs, or by pharmacological inhibitors specific for each PIKK, has no significant effects on HIC1 SUMOylation (Figure 1C). Previously, we demonstrated that SUMOylation potentiates the repressive potential of HIC1 by favoring its interaction with MTA1 [10, 11], most notably during the response to non-repairable DSBs [8]. However, after induction of repairable DSBs, co-immunoprecipitation experiments in HEK293T cells transfected with expression vectors for HIC1 and MTA1 clearly failed to demonstrate a stronger interaction between these two proteins (Figure 1D).

In conclusion, repairable and non-repairable DSBs, induced by 1 hour or 16 hours etoposide treatments respectively, have different impacts on HIC1 SUMOylation and hence on the interaction between HIC1 and the NuRD complex, potentially in line with the biological outcomes of the DNA damage responses to these different genotoxic insults, repair or apoptosis.

### Lack of HIC1 SUMOylation does not impair DNA repair

**Figure 2:**
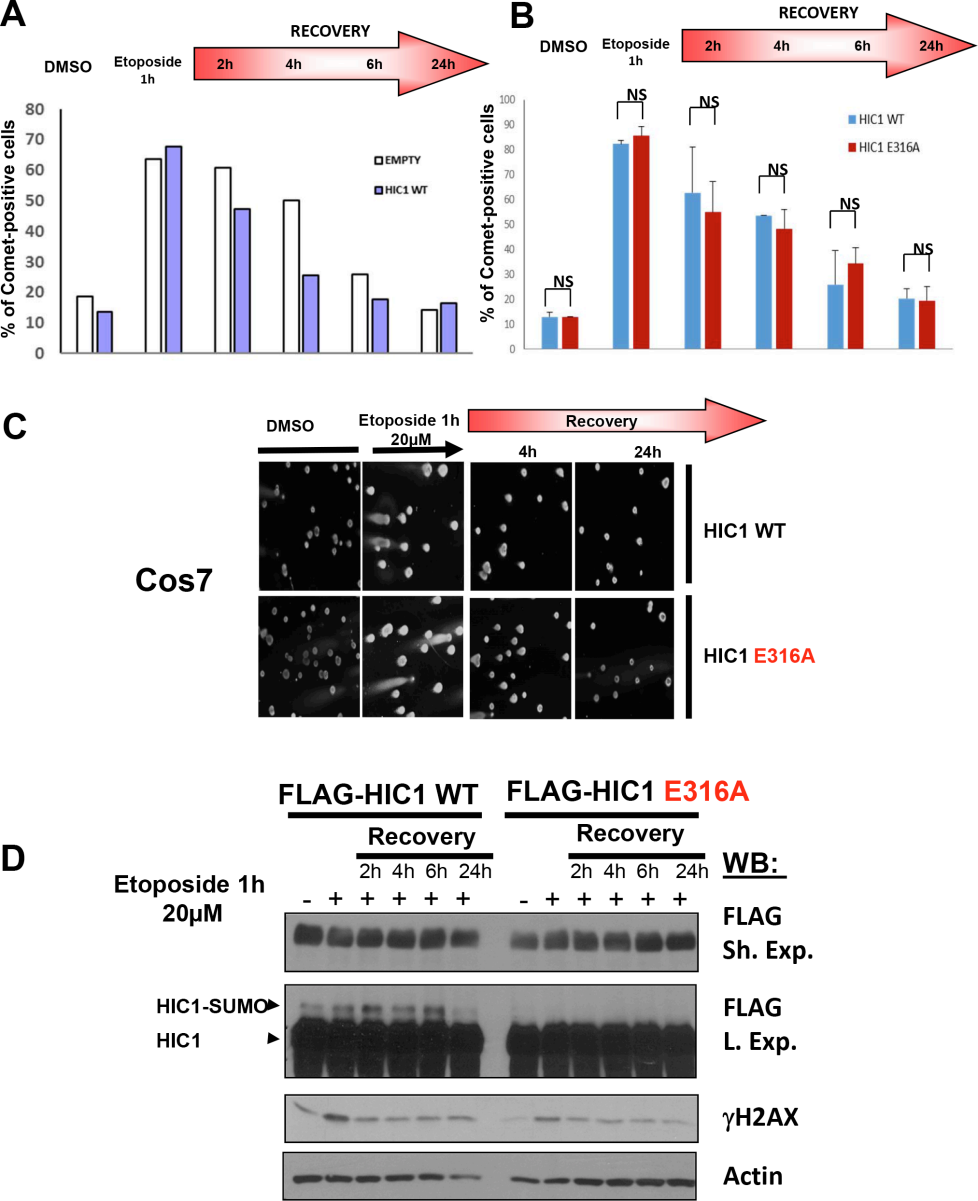
HIC1 SUMOylation is not required for efficient DSBs repair (**A**) Cos7 cells were transfected for 48 hours with wt FLAG-HIC1 or with the empty pcDNA3FLAG expression vector. Cells were either mock-treated with DMSO (–) or treated with 20 μM etoposide (+) for 1 hour. After removal of the drug, cells were allowed to recover in normal medium for various times (2, 4, 6 and 24 hours) and DSBs were monitored by neutral Comet assay. The percentage of Comet positive cells reflecting unrepaired DNA breaks is depicted after counting at least 100 cells in each condition. (**B**) Cos7 cells were transfected for 48 hours with wt FLAG-HIC1 or with the non-SUMOylatable E316A point mutant. Neutral Comet assays were performed and analyzed as described in panel A). The error bar indicates mean +/– standard deviation of three independent experiments (NS: not significant). (**C**) Representative Comet images of mock-treated (DMSO) and of cells treated with etoposide for 1 hour after transfection of wt HIC1 or of E316A HIC1 with or without recovery in normal medium for 4 and 24 hours, respectively. (**D**) Western blot analyses of cells transfected with wt HIC1 or with E316A HIC1 Samples of cells in each condition were taken before the Comet assays and immediately lysed in Laemmli loading buffer. These whole cell extracts were analyzed by Western blot with anti-FLAG antibodies to detect HIC1 and its SUMOylated forms. γH2AX and actin levels were used as controls for DSB induction and equal loading, respectively.

To address the functional impact of HIC1 SUMOylation on the time-course of DSBs repair, we first tested wt HIC1 and the empty FLAG expression vector in the neutral Comet assay which specifically measured DSBs at the level of individual cells. To that end, 48 hours after transfection, Cos7 cells which do not express HIC1 at significant endogenous levels were treated for 1 hour with etoposide before recovery in complete culture medium without etoposide for various times. As shown in Figure 2A, ectopic expression of HIC1 slightly accelerates DSBs repair notably during the early steps of recovery (2 h and 4 h). These observations are in close agreement with the slower repair induced in BJ-hTERT human fibroblasts by inactivation of endogenous *HIC1* expression through siRNA interference [8]. We next compared the DNA repair capacity of HEK293T cells transfected with wt HIC1 or with the non SUMOylatable E316A point mutant. HIC1 Lysine 314 can be acetylated and SUMOylated [10, 11]. Therefore, we used the E316A mutant (non SUMOylatable since the SUMOylation consensus is ϕKxE) instead of the K314R mutant since this latter would impede not only SUMOylation but also acetylation or any other potential post-translational modifications on this lysine residue [14]. Results showed that the same amount of DSBs were induced in HEK293T transfected with the two expression vectors. Furthermore, no salient differences were observed during the time-course recovery in normal medium of cells expressing the wt or the E316A SUMO-deficient HIC1 mutant (Figure 2B–2D). Thus, these results unambiguously demonstrate that HIC1 SUMOylation on lysine K314 is not essential for DSBs repair.

### HIC1 SUMOylation increase after 16 h etoposide treatment is dependent of ATM

**Figure 3:**
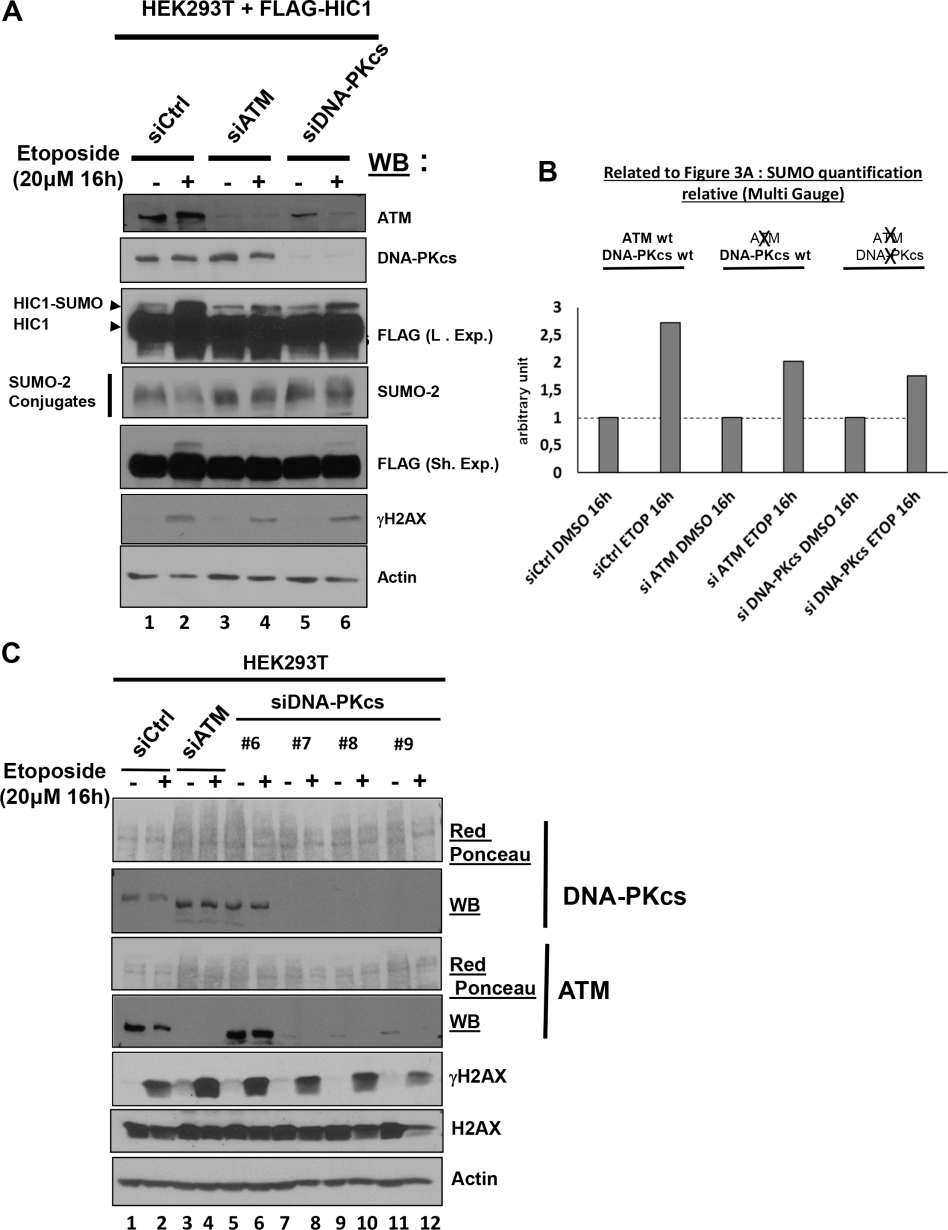
The SUMOylation increase of HIC1 upon induction of irreparable DSBs is dependent on ATM but independent of DNA-PKcs (**A**) HEK293T cells were transfected either with nontargeted control siRNA (siCtrl), either with a pool of four siRNAs targeting ATM (siATM) or with a pool of four siRNAs targeting DNAPKcs (siDNAPKcs). The next day, these cells were transfected with a FLAG-HIC1 expression vector for 24 hours and were then treated with 20 μM etoposide (+) or mock-treated with DMSO (–) as control for 16 hours before direct lysis in denaturing conditions. Total cell extracts were analyzed by Western Blotting (WB) using the indicated antibodies. (**B**) Quantification of SUMO-HIC1. The HIC1 SUMOylated band in control conditions (siCtrl, DMSO 16 h; lane 1 in panel A) was quantified with the Fujifilm MultiGauge software and given the arbitrary value of 1. The other HIC1 SUMOylated bands (lanes 2 to 6 in panel A) were quantified relative to this value. (**C**) HEK293T cells were transfected either with nontargeted control siRNA (siCtrl), a pool of four siRNAs targeting ATM (siATM) or with each individual siRNA from the pool targeting DNA-PKcs (siDNA-PKcs). Then, cells were treated with etoposide and total cell extracts were analyzed by Western blot on three different gels (two 6% polyacrylamide gels for DNAPK-cs and ATM; a 15% polyacrylamide gel for γH2AX, H2AX and actin) as described in panel A.

DSBs elicit a DNA damage response primarily relying on the activation of the ATM or DNA-PKcs kinases which have complementary and non-redundant functions [13, 15]. Whereas ATM has hundreds of substrates, DNA-PKcs phosphorylates a smaller group of proteins involved in DSBs end joining. [16]. We tried to inhibit ATM and DNA-PKcs by a 1 hour pre-treatment with Wortmaninn or with specific pharmacological inhibitors for each PIKK prior to a 16 hours etoposide treatment to induce non-repairable DSBs in the presence of these inhibitors as previously performed with the ATM specific inhibitor [8]. However, in these conditions where the DNA-PKcs inhibitor was kept on the cells for 17 (1+16) hours, a strong cytotoxic effect precluding further analyses was observed (data not shown). To circumvent this technical problem, we then used siRNA interference to inactivate DNA-PKcs or ATM, as a positive control. Upon induction of irreparable damage, the increase of HIC1 SUMOylation was observed in cells tranfected with control siRNAs and also in cells transfected with a pool of siRNAs efficiently targeting ATM albeit to a lesser extent (Figure 3A, lanes 1 to 4 and Figure 3B). These findings therefore nicely confirmed our previous results obtained with the pharmacological ATM inhibitor, Ku-55933 [8]. Silencing of DNA-PKcs by siRNAs did not fully abolish the increase of HIC1 SUMOylation but did significantly impair it to levels similar to those obtained with ATM siRNAs (Figure 3A, lanes 5 and 6 and Figure 3B). However, in the control Western blots, we noticed that this pool of siRNAs targeting DNA-PKcs down regulate not only the expression of DNA-PKcs but also surprisingly the expression of ATM (Figure 3A, lanes 5 and 6). These results were confirmed by qRT-PCR analyses of ATM expression levels in the same transfected cells (Supplementary Figure S2). To explain this, we performed another experiment testing individually the four siRNAs of the pool targeting DNAPKcs. In Western blots, the three siRNAs that efficiently inhibit DNA-PKcs expression (#7, #8 and #9) also inhibit ATM expression whereas the siRNA DNA-PKcs #6 has no effect on DNAPK-cs and ATM expression (Figure 3C, lanes 5 to 12). As expected, the control siRNAs have no effect on ATM and DNAPK-cs and the pool of siRNAs targeting ATM has no effect on DNA-PKcs expression (Figure 3A, lanes 1 to 4). After a 16 h etoposide treatment, the SUMOylation increase observed in cells transfected with the ATM siRNAs pool which express DNA-PKcs and in cells tranfected with the DNAPK-cs siRNAs pool, in which the expression of both DNAPK-cs and ATM are severely inhibited, appears reduced but similar (Figure 3A and 3B). Thus, the increase of HIC1 SUMOylation observed upon induction of irreparable DSBs appears to be primarily dependent upon ATM.

### HIC1 SUMOylation increase is dependent on the apical kinase ATM but independent of its effector kinase Chk2

**Figure 4:**
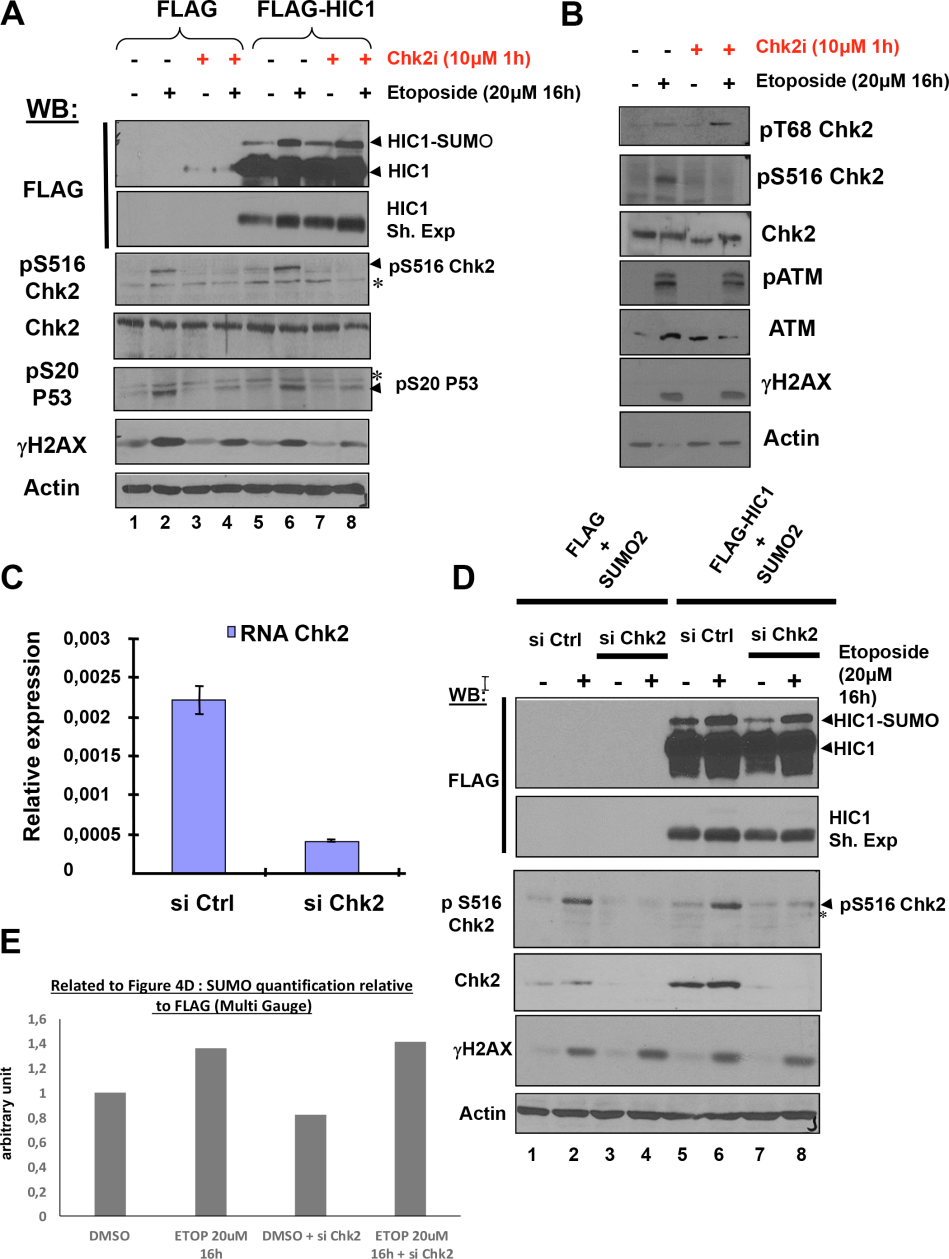
The increase of HIC1 SUMOylation upon irreparable DSB induction by etoposide requires ATM but not its effector kinase Chk2 (**A**) HEK293T cells were transfected with the FLAG and FLAG-HIC1 vectors. 48 hours after transfection, cells were pre-incubated or not with the Chk2 inhibitor (Chk2i) for 1 hour and then with etoposide for 16 hours as indicated. Cell extracts were prepared and Western blotting was performed with the indicated antibodies. *refers to non-specific bands detected by the anti pS516Chk2 (autophosphoylation) and by the anti pS20P53 (Chk2 target) antibodies. γH2AX and actin levels were used as controls for DSBs induction and equal loading, respectively. (**B**) To control for the inhibition of Chk2, HEK293T cells were transfected and pre-incubated or not with the Chk2 inhibitor (Chk2i) for 1 hour and then with etoposide for 16 hours exactly as in panel A) before lysis and Western blot analyses with the indicated antibodies (**C**) HEK293T cells grown in normal medium were transfected with siRNA control (siCtrl) or with a Chk2 siRNA pool (siChk2). Total RNAs were extracted and the mRNA expression levels of *Chk2* were assessed by qRT-PCR. Values were normalized to *18S*. (**D**) HEK293T cells were transfected either with non-target control siRNA (siCtrl) or with a Chk2 siRNA pool (siChk2) before being transfected with the indicated combination of FLAG, FLAG-HIC1 and SUMO-2 expression vectors. Cells were either incubated with DMSO (–) or with 20 μM etoposide (+) for 16 hours. Total cell extracts were prepared and analyzed by Western blotting with the indicated antibodies. *refers to a non-specific band detected by the anti pS516Chk2, used as a control for Chk2 kinase activity. γH2AX and actin levels were used as controls for DSBs induction and equal loading, respectively. (**E**) Quantification of SUMO-HIC1 to total HIC1 (FLAG) for lanes 5 to 8 in panel D) was performed with the Fujifilm MultiGauge software.

After detection of DSBs by sensors such as the MRN complex, DNA damage signaling is rapidly induced by the activation of the ATM/Chk2 pathway. Whereas the apical ATM kinase is recruited to and stabilized on the DSBs sites, its effector kinase Chk2 becomes phosphorylated by ATM at damage sites but then rapidly dissociates and is distributed throughout the nucleus to phosphorylate numerous downstream targets [2, 17]. To determine whether the HIC1 SUMOylation increase after irreparable DSB induction requires the complete activation of the ATM/Chk2 pathway and is dependent on both ATM and Chk2 activation, FLAG-HIC1-transfected cells were pre-incubated with C3742, a specific inhibitor of Chk2, prior to a 16 hour etoposide treatment (Figure 4A). Both the basal and enhanced SUMOylation of HIC1 observed after induction of irreparable DSBs remained unchanged in presence of the Chk2 inhibitor (Figure 4A, lanes 4 to 8). Similar results were obtained with cells treated with etoposide for 1 hour (Supplementary Figure S4). As controls for the effectiveness of the Chk2 inhibitor, we observed a strong decrease of Chk2 autophosphorylation on Serine 516 as well as of phosphorylation of P53 on Serine 20, a well-known target of Chk2 but no significant effects on two ATM substrates (T68Chk2 and γH2AX) or on ATM autophosphorylation (Figure 4A and 4B).

To confirm these results with an independent assay, we used siRNA interference to inactivate Chk2. We first demonstrated the efficiency of a pool of siRNAs (Dharmacon) targeting Chk2 by transfection in HEK293T grown in standard conditions (no etoposide treatment) followed by qRT-PCR and Western blot analyses (Figure 4C and 4D). Using this pool of siRNAs targeting Chk2, we further showed that the increase of HIC1 SUMOylation observed after a 16 hour etoposide treatment is not significantly affected by Chk2 inhibition (Figure 4D and 4E).

In conclusion, the increase of HIC1 SUMOylation upon induction of non-repairable DSBs is dependent upon the apical kinase ATM [8] but not its downstream effector kinase Chk2. Thus, these results strongly suggest that the increase of HIC1 SUMOylation occurs and plays a role in close proximity to chromatin where ATM is activated and stabilized consistent with the “on-site modification” model proposed for SUMOylation.

### The SIM (SUMO-interacting motif) of MTA1 is required for the interaction with HIC1

**Figure 5:**
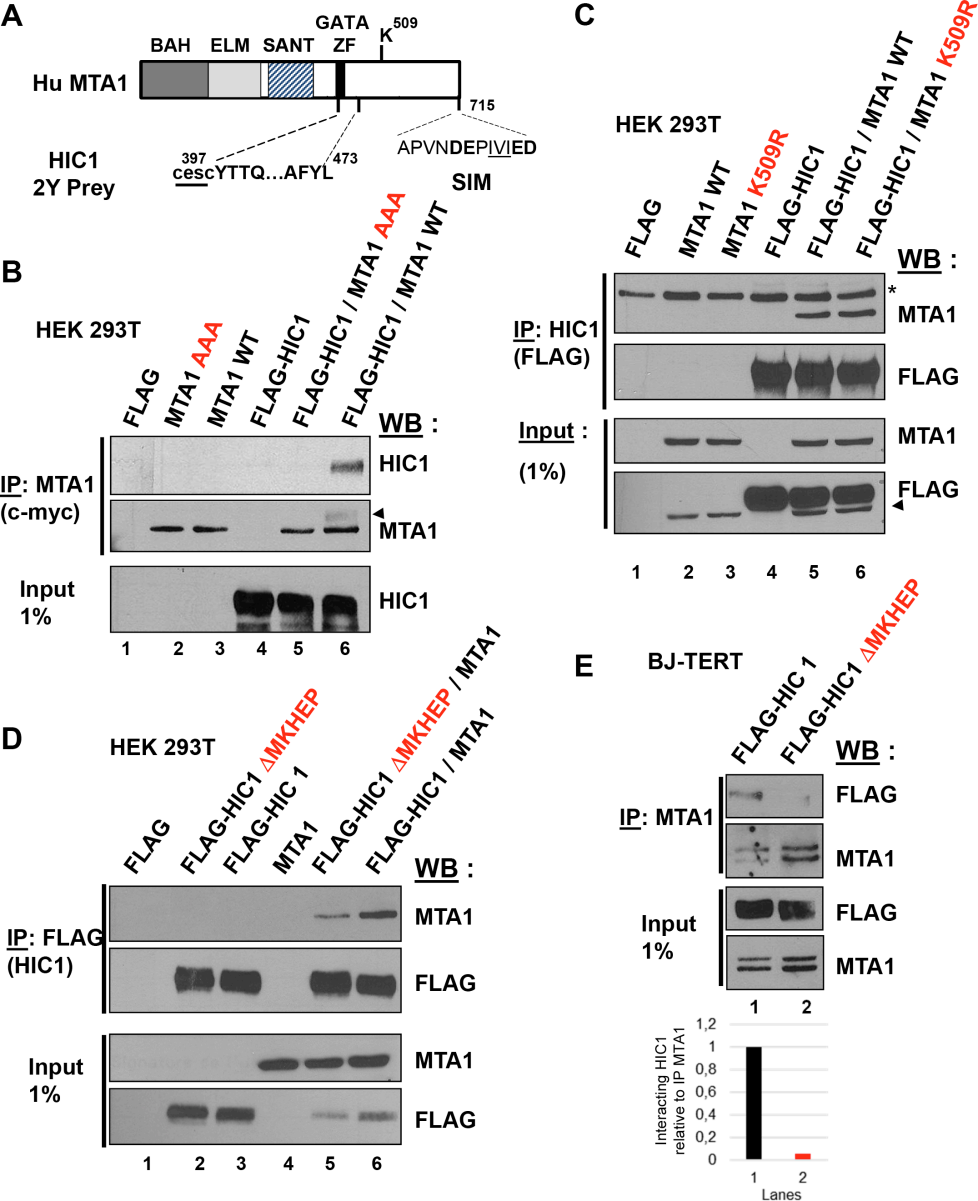
At least two domains in the C-terminal end of MTA1 are implicated in the interaction with HIC1 (**A**) Schematic drawing of the human MTA1 protein. The domains identified in MTA1 include the BAH (Bromo-associated homology), the ELM (Egl-27 and MTA1 homology), the SANT (SW13, ADA2, N-CoR and TF1118) and the GATA-like zinc finger. The region isolated in the two-hybrid screen with HIC1 is shown with the two first cysteines of the GATA zinc finger not present in the isolated prey, shown as lower-case letters underlined [11]. The Lysine 509, which is SUMOylated, and the C-terminal SIM motif are also shown with the hydrophobic core, IVI, underlined and the flanking acidic residues in bold [19]. (**B**) The SIM (SUMO-interacting motif) in the C-terminal end of MTA1 is required for its interaction with HIC1. After transfection with the indicated expression vectors, HEK293T cells lysates were immunoprecipitated with anti-c-myc antibodies. Immunoprecipitated samples [IP c-myc (MTA1)] and 1% of whole cell extracts (Input) were analyzed by immunoblotting with anti-HIC1 antibodies to detect co-immunoprecipitation. To control for IP efficiency, the membrane was stripped and probed with anti-MTA1 antibodies (the arrow head indicates a remnant of the HIC1 band). (**C**) SUMOylation of MTA1 on Lysine K509 is not required for the HIC1-MTA1 interaction. A similar experiment was performed in HEK293T with expression vectors for wt MTA1 or its non-SUMOylatable version (K509R) and HIC1. In the top panel, *refers to a non-specific band detected. In the bottom panel, the arrowhead indicates a remnant of the MTA1 band. (**D**) The HIC1-MTA1 interaction is also strongly reduced by deletion of the HIC1 SUMOylation motif, ΔMKHEP. A similar Co-IP experiment was realized in HEK293T but with expression vectors for the wt FLAG-HIC1 or the FLAG-HIC1 ΔMKHEP deletion mutant and wt MTA1. (E) Interaction of wt and ΔMKHEP HIC1 with endogenous MTA1 proteins in HEK293T cells. Total extracts of HEK293T transfected with the indicated plasmids were analysed by Co-IP with anti-MTA1 antibodies and immunoblotted with MTA1 and FLAG antibodies. Note that the endogenous MTA1 proteins in the immunoprecipitated materials or in the Inputs migrate as a doublet.

Since the induction of non-repairable DSBs and the resulting increase in SUMOylation of HIC1 favors its interaction with MTA1, we further investigated the molecular mechanisms underpinning the HIC1-MTA1 interaction with a focus on SUMOylation [8, 11]. A SUMOylated protein can interact non-covalently with another protein containing a SIM (SUMO-interacting motif) [14]. MTA1 contains in its C-terminal end a functional SIM motif DEPIVIED (Figure 5A) perfectly fitting with the most canonical class of SIM motifs, a hydrophobic core (V/I)X(V/I)(V/I) flanked by acidic amino acids [18, 19]. Interestingly, triple mutations in the hydrophobic core of the SIM motif in MTA1 (I711A/V712A/I713A, referred to hereafter as AAA) abolished the interaction of MTA1 with HIC1 (Figure 5B, lane 5). MTA1 is also SUMOylated on Lysine 509, which is located in its C-terminal region [19]. However, in contrast with the AAA SIM mutant, wt MTA1 and the K509R non-SUMOylatable mutant similarly interacted with HIC1 in co-immunoprecipitation experiments, demonstrating that MTA1 SUMOylation is not required for this interaction (Figure 5C, lane 5 and 6).

Thus, the SIM motif of MTA1 is essential for the interaction with HIC1, in agreement with the fact that HIC1 SUMOylation favors it, and thus highlights a SUMO-SIM non-covalent interaction between these two proteins [11]. However, the region of interaction between HIC1 and MTA1 previously defined by the prey isolated in the yeast two-hybrid screening as MTA1 amino-acids 397-473 excluded this MTA1 SIM motif (Figure 5A) and the HIC1 K314R non-SUMOylatable mutant still interacts with MTA1, albeit weakly [11]. To elucidate this interaction, we constructed another mutant of the full length HIC1 protein, hereafter referred to as HIC1 ΔMKHEP, by deleting amino acids 305-326 encompassing this SUMOylation motif. In co-immunoprecipitation experiments, the ΔMKHEP HIC1 mutant interacts very weakly with ectopically expressed MTA1 proteins as compared to wt HIC1 (Figure 5D, lanes 5 and 6). Furthermore, this deletion mutant upon overexpression in HEK293T cells is almost unable to co-immunoprecipitate with endogenous MTA1 proteins (Figure 5E, lane 2). Reciprocally, we fused the isolated SUMOylation motif of HIC1 (amino acids 305-326) in frame with a C-terminal DNA-binding domain, a nuclear localization signal and an HA epitope in the Gal4-NLS-HA vector to mimic its localization in the full-length protein. However, this 305-326-HIC1-Gal4 chimera despite being nuclear and displaying a strong repression potential in transient Luciferase reporter assays, is unable to significantly interact with MTA1 (data not shown).

All together these results suggest a complex, multi-domain interaction between HIC1 and MTA1 with major, but not exclusive, roles played by the HIC1 SUMOylation motif and the MTA1 SUMO-interacting motif.

### HIC1 interacts with MTA3 and this interaction increases upon induction of non-repairable DSBs

**Figure 6:**
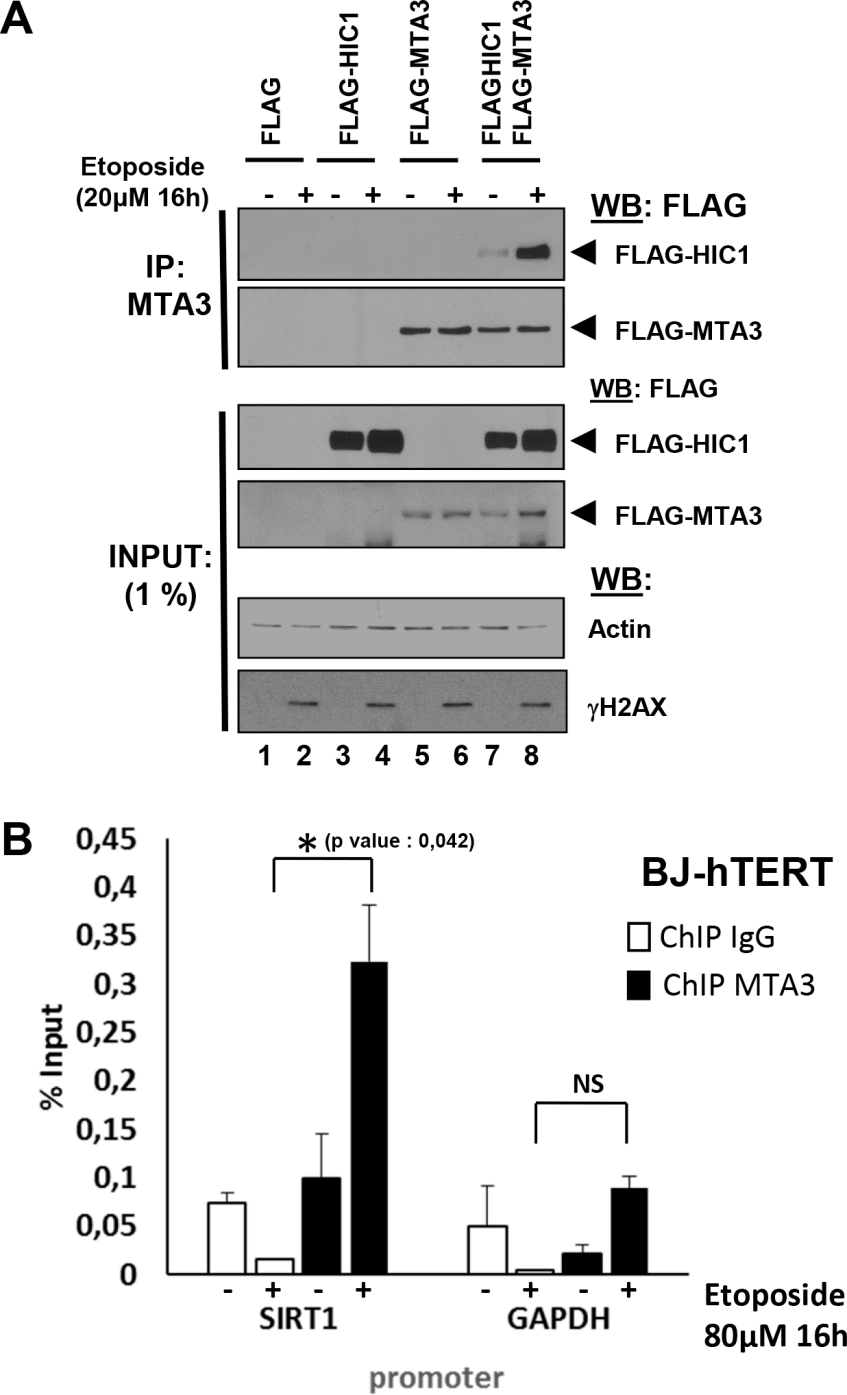
Irreparable DSBs induced by a 16 hour etoposide treatment lead to an increased interaction of MTA3 with HIC1 and favor its recruitment to the HIC1-response elements in the SIRT1 promoter (**A**) Etoposide-induced non-repairable DSBs lead to an increase of MTA3 interaction with HIC1. HEK293T cells were transfected with the indicated combination of empty FLAG, FLAG-HIC1, and FLAG-MTA3 expression vectors. 32 hours after transfection cells were incubated for 16 hours with 20 μM etoposide (+) or with DMSO (–) as control. After lysis in IPH buffer, cell extracts were co-immunoprecipitated with anti-MTA3 antibodies. The immunoprecipitates as well as 1% of the whole cell extracts were analyzed by SDS/PAGE and transferred to membranes. Relevant pieces of the membranes were cut and analyzed by Western blot with anti-FLAG antibodies to detect MTA3 and HIC1. ΔH2AX and actin levels were used as controls for DSB induction and equal loading, respectively. (**B**) Etoposide-induced irreparable DSB lead to an increase of MTA3 recruitment on the HiRE in the *SIRT1* promoter. Chromatin was prepared from BJ-hTERT fibroblasts mock-treated with DMSO or treated with 80 uM etoposide for 16 hours to induce irreparable DSB and ChIP experiments were performed with antibodies against MTA3 or rabbit IgG. The bound material was eluted and analysed by quantitative PCR using primers flanking the HIC1-responsive elements (HiRE) in the *SIRT1* promoter [6], as previously described [46]. *GAPDH* was used as a nonbinding control. Values that are statistically significantly different are indicated by bars and asterisks as follows: *P < 0.05. NS corresponds to values that are not statistically significantly different.

MTA1, the closely related MTA2 and the functionally distinct MTA3 proteins are found in a mutually exclusive manner in different specialized NuRD complexes [20, 21]. Given that HIC1 interacts with MTA1 and this interaction is favoured by HIC1 SUMOylation [8, 11], we thus investigated if HIC1 also interacts with MTA3 and if this interaction increased upon induction of non-repairable DSBs. Co-immunoprecipitation experiments (Co-IPs) in transiently transfected HEK293T cells demonstrated that HIC1 interacts with MTA3 and that this interaction strongly increased when cells were pre-treated with etoposide for 16 hours (Figure 6A, lanes 7 and 8).

To correlate these results with promoter occupancy and transcriptional regulation, we next performed ChIP experiments with chromatin prepared from BJ-hTERT fibroblasts treated or not with etopside for 16 hours. Using high quality ChIP-grade antibodies for MTA3 [22], these experiments demonstrated a strong enrichment of MTA3 binding onto the HIC1 responsive elements (HiRE) in the *SIRT1* promoter [6, 11] upon induction of irreparable damage (Figure 6B). Thus, the increase of HIC1 SUMOylation after a 16 hours etoposide treatment is nicely correlated with an increase in HIC1-MTA3 interaction, thus favoring the recruitment of MTA3 onto HIC1 direct target genes. Taken together, these results demonstrate that HIC1 can interact with the MTA1/MTA2 and MTA3 proteins and hence with a wide variety of NuRD complexes and that these repressive complexes are favoured to repress some HIC1 target genes and notably *SIRT1* during the apoptotic DNA damage response to non-repairable DSBs.

### Differential HIC1 recruitment on the SIRT1 promoter upon induction of repairable versus non-repairable DSBs

**Figure 7:**
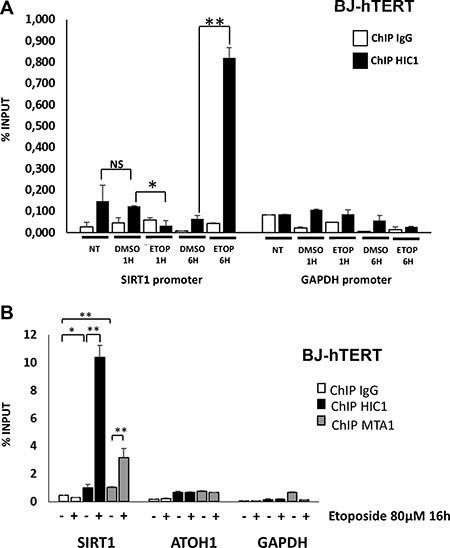
HIC1 and MTA1 recruitment to the HIC1-response elements in the SIRT1 promoter is increased upon induction of non-repairable DSBs (**A**) HIC1 recruitment to the HiRE in the *SIRT1* promoter in various conditions. Chromatin was prepared from BJ-hTERT fibroblasts not-treated, mock-treated with DMSO or treated with 80 μM etoposide for various times and ChIP experiments were performed with antibodies against HIC1 or rabbit IgG. The bound material was eluted and analysed by quantitative PCR using primers flanking the HIC1-responsive elements (HiRE) in the *SIRT1* promoter [6], as previously described [46]. *GAPDH* was used as a nonbinding control. (**B**) Etoposide-induced irreparable DSBs lead to an increase of HIC1 and MTA1 recruitment to the HiRE in the *SIRT1* promoter. Chromatin was prepared from BJ-TERT fibroblasts mock-treated with DMSO or treated with 20 mM etoposide for 16 hours to induce irreparable DSBs and ChIP experiments were performed with antibodies against HIC1, MTA1 or rabbit IgG as described in panel A). Values that are statistically significantly different are indicated by bars and asterisks as follows: *P < 0.05, **P < 0.01. NS corresponds to values that are not statistically significantly different.

We next compared HIC1 binding of the *SIRT1* promoter upon induction of repairable or non-repairable DSBs. We first performed pilot ChIP experiments with BJ-hTERT cells treated or not with DMSO (vehicle) or Etoposide for various times. Whereas HIC1 is bound on the *SIRT1* promoter in control (untreated) conditions, this binding is slightly decreased in the presence of DMSO, especially after the longest treatment times (6 hours) (Figure 7A). ChIP experiments conducted with BJ-hTERT cells treated for 1 h with Etoposide detected a clear decrease of HIC1 binding to the *SIRT1* promoter (Figure 7A). By contrast, a strong increase of HIC1 binding is observed upon induction of non-repairable damage. Notably, SUMOylation of the related BTB/POZ transcriptional repressor PLZF also increases its DNA binding properties [23, 24]. In an independent experiment, HIC1 binding and the recruitment of MTA1 to the *SIRT1* promoter were also increased after a 16 hours etoposide treatment (Figure 7B, left columns). The proneuronal HIC1 target gene, *ATOH1* and GAPDH were used as controls. Thus, these data demonstrate that the induction of non-repairable DSBs increased the binding of HIC1 and its SUMOylation-dependent partners, MTA1 and MTA3, to the *SIRT1* promoter.

### A short 6 hours etoposide treatment is sufficient to induce a P53-dependent apoptotic response

HIC1 directly represses *SIRT1* transcription to modulate the P53-dependent apoptotic response to non-repairable double-strands breaks [6]. Our previous work [8] [11] and the results reported in this study all emphasize an increased HIC1 SUMOylation which favors its interaction with MTA1 or MTA3 in NuRD repressive complexes and hence enhances its transcriptional repression activity during the cellular response to irreparable DSBs (Figures 1 and 5–7). However, a long, 16 hours etoposide treatment could induce a direct transcriptional effect mediated by HIC1 but also an indirect, “second-wave” effect mediated by P53 which is acetylated and hence activated through *SIRT1* inhibition by HIC1. Indeed, in these conditions the pro-apototic *Bax*, *Noxa* and *PUMA* genes and the anti-apoptotic *BCL-2* gene which are not known as direct target genes of HIC1 but as direct P53 target genes are activated or repressed, respectively by P53 [6]. To address this issue, we first performed a pilot time-course response of BJ-hTert fibroblasts to etoposide. BJ-hTERT cells transfected with Control siRNAs or HIC1 siRNAs were treated with etoposide for 1, 6 and 16 hours and analyzed by Western blot for expression of the cell cycle inhibitor P21^CIP1^ which is a key factor mediating the P53 response and also a direct target gene of HIC1 [25]. A similar induction of P21^CIP1^ expression is observed after either 6- or 16- hour etoposide treatments as well as a further increase in siHIC1-treated cells as expected for a HIC1 direct target gene (Supplementary Figure S5A). Thus, a shorter 6 hours etoposide treatment is sufficient to increase HIC1 recruitment to the *SIRT1* promoter (Figure 7) and to induce a P53-dependent apoptotic response while limiting the induction of indirect HIC1 target genes.

### Identification of new potential target genes regulated by HIC1 SUMOylation

**Figure 8:**
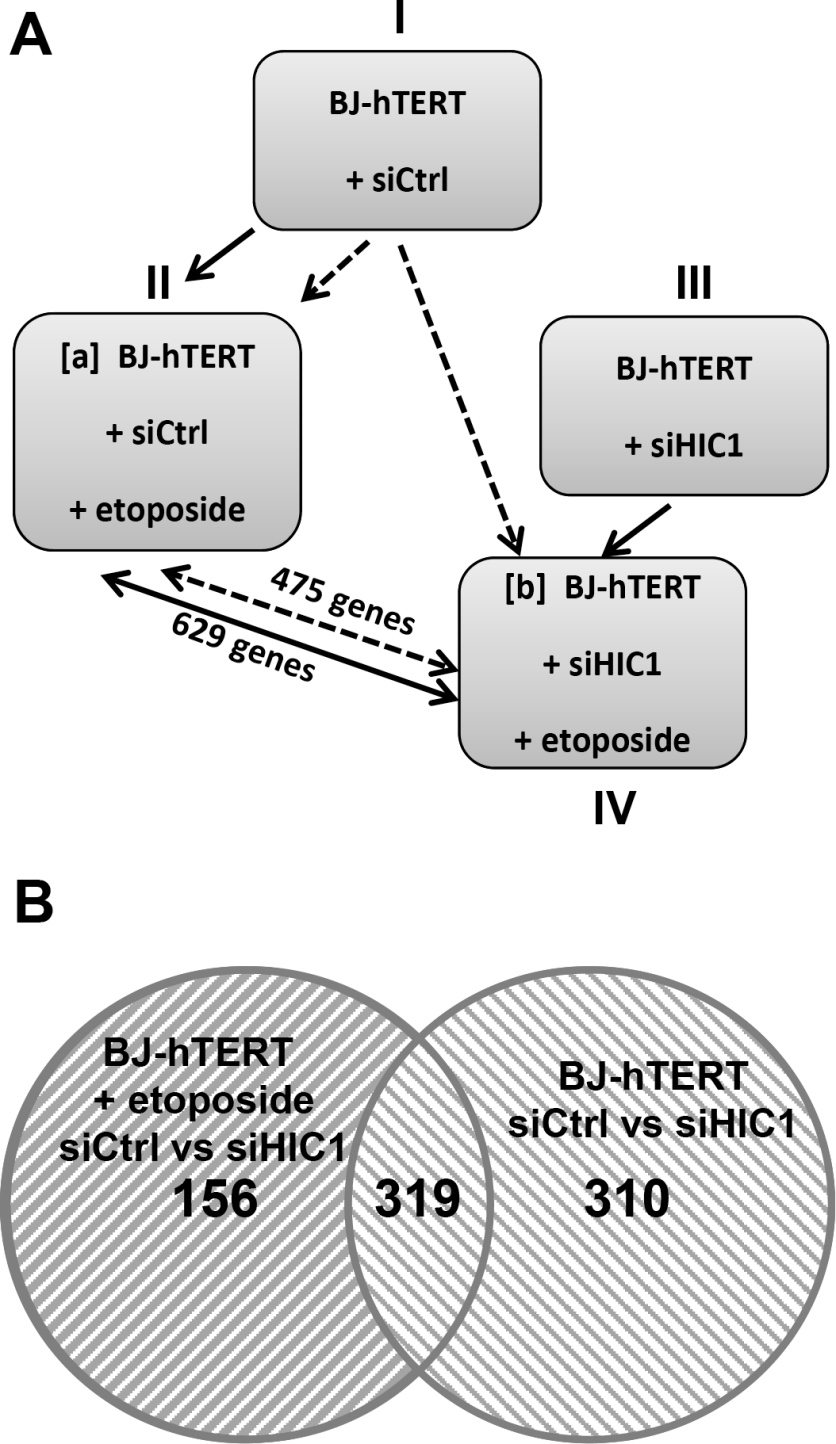
Identification of the genes regulated in BJ-hTERT human fibroblasts by HIC1 in the presence and absence of etoposide to induce irreparable DSB (**A**) Schematic drawing of the experimental design of the study and of the 2 normalization strategies used to identify the genes regulated by HIC1 in the presence and absence of a 6 hours etoposide treatment to induce irreparable DSBs. Four experimental conditions were used and compared using two different comparison strategies. In the first comparison strategy (Strategy #1 : normalization of IV to III, shown as black lines), BJ-hTERT siCtrl cells treated with etoposide were compared to control cells (siCtrl, no etoposide) to define genes repressed by etoposide and thus containing a subset of genes repressed by SUMOylated HIC1. Then, BJ-hTERT siHIC1 cells treated or not with etoposide were compared to define genes still repressed by etoposide in a HIC1-deficient context. Subtracting [IV] from [II] yields 629 target genes repressed in response to DSBs and dependent upon HIC1 SUMOylation. In the second strategy (Strategy #2: normalization of IV to I, shown as dotted lines), BJ-hTERT siCtrl and BJ-hTERT siHIC1 cells, both treated with etoposide, were each compared to control cells (siCtrl, no etoposide). In that case, subtracting [IV] from [II] yields 475 target genes. (**B**) Venn diagrams showing the overlap of the 629 and 475 target genes from the 2 normalization strategies used yields a strong overlap of 319 genes (see text for detail).

**Figure 9:**
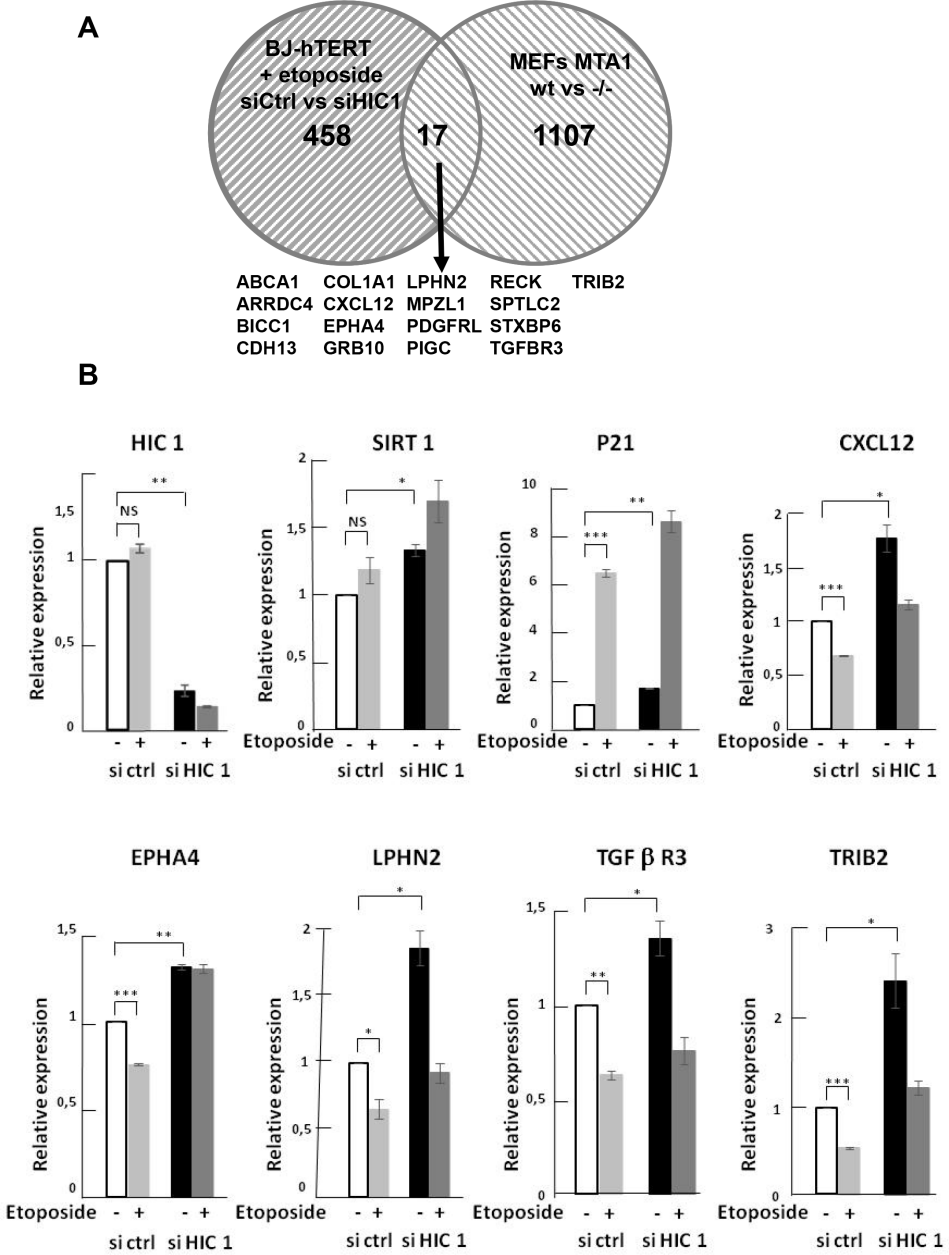
Identification of target genes potentially regulated in BJ-hTERT human fibroblasts by HIC1 SUMOylation and MTA1 (**A**) Venn diagrams showing the overlap of the 475 target genes from the normalization strategy #2 and the 1024 genes affected by MTA1 knock out in murine embryonic fibroblasts (MEFs) [26]. (**B**) Validation by qRT-PCR of the microarray data showing differential regulation of some selected genes in BJ-hTERT cells. RNAs were extracted from BJ-hTERT using the same four experimental conditions as in the microarrays analyses. Selected genes (*CXCl12*, *EPHA4*, *LPHN2*, TGFβR3 and *TRIB2)* were analysed by qRT-PCR analyses and showed the expected differential regulation (see text for detail). The expression of *HIC1* and of its direct target genes *SIRT1* and *P21* were also tested as positive controls.

Given our findings of the important role played by HIC1 SUMOylation in the transcriptional response to irreparable DSBs, we chose to employ a gene expression profiling approach to identify candidate genes for HIC1-mediated transcriptional repression after the induction of DNA damage. BJ-hTERT cells transfected with either siHIC1 (HIC1^-^) or sicontrol (HIC1^+^) oligomers were subjected to etoposide induced DNA damage. Western blot analyses confirmed the efficient inactivation of HIC1 by the siRNA as well as the induction of P53 and p21 after etoposide treatment (Supplementary Figure S5B). Total RNA was isolated from treated and untreated cells in triplicate and subjected to gene expression profiling using the ILLUMINA HumanHT-12 v4 Expression BeadChip kit. To identify potential target genes, two normalization strategies were employed. In the first, HIC1^+^ and HIC1^-^ etoposide treated cells (conditions II and IV) were first normalized to their untreated counterparts (conditions I and III) to identify genes that became repressed as a result of DNA damage. In order to determine which of those genes were candidates for SUMO-HIC1 mediated repression, the list of repressed genes in HIC1^-^ cells was subtracted from those repressed in HIC1^+^ cells yielding 629 genes repressed in a HIC1 replete context and not in the absence of HIC1 (Figure 8A, solid lines; Supplementary Table S1). In the second normalization strategy, mRNA expression from both the HIC1^+^ and HIC1^-^ BJ-hTERT cells that were treated with etoposide (conditions II and IV) were compared back to the HIC1 sufficient untreated cells (condition I). The transcripts that were significantly repressed in the HIC1^-^ context were subtracted from those repressed in the HIC1^+^ cells leaving 475 genes whose repression was potentially mediated by HIC1 (Figure 8A, dashed lines; Supplementary Table S2). The agreement between the resulting gene lists was substantial with 319 genes representing the intersection of these gene sets (Figure 8B, Supplementary Table S3). It is important to note that these genes likely represent both direct and indirect HIC1-mediated transcriptional repression. The union of these lists (785 genes) was used to identify interaction maps using Ingenuity Pathway Analysis software. The top canonical pathway mapped by the repressed gene set was “Role of BRCA1 in DNA Damage Response”. Measured by the number of molecules represented, “Cell Death” and “Cell Cycle” were the top cellular functions identified with 80 and 58 genes represented respectively (data not shown). Since HIC1 SUMOylation increases its interaction with MTA1, we next compared these HIC1 target genes with MTA1 target genes obtained through gene profiling experiments of wt and Mta1–/– murine embryos fibroblasts (MEFs) [26]. This comparison highlighted 17 genes (Figure 9A). Among them, *CXCl12*, *EPHA4*, *LPHN2*, TGFβR3 and *TRIB2* were validated by qRT-PCR as potential target genes regulated by HIC1 SUMOylation. Indeed, they are activated in siHIC1 BJ-hTERT fibroblasts as compared to siCtrl cells in control conditions (DMSO) and further repressed in siCtrl cells treated with etoposide (Figure 9B). Thus, we were able to place HIC1 and its interaction with NuRD complexes through its increased SUMOylation among the canonical pathways responsible for the transcriptional response to irreparable DSBs while yielding further potential targets of HIC1 for future validation.

## DISCUSSION

The cellular response to different types of DNA damages involves a complex interplay of various post-translational modifications including phosphorylation by PIKK kinases, Ubiquitinylation, PARylation and SUMOylation among many others [27–29]. SUMOylation is a very dynamic post-translational modification, involved essentially in regulation of protein-protein interaction and organization of macromolecular complexes through SIMs (SUMO-interacting motifs) that allow effector proteins to engage SUMO-modified substrates [12, 30]. Numerous proteins modified by SUMOylation have been identified and many of them are associated with transcriptional repression [30, 31]. SUMOylation is also a very labile PTM affecting only a small percentage of target proteins, the so-called “SUMO paradox” [12] and is thus difficult to detect. It is also becoming increasingly clear that SUMOylation plays a key role in the regulation of DNA damage repair and responses with, for example, an increased presence of SUMO at sites of DNA damage [27] and an orchestrated SUMOylation of subsets of chromatin remodelers to decrease global transcription upon DNA damage [32, 33]. SUMOylation is also implicated in another important aspect of the repair process, determining the kinetics and mechanisms for the repair of DSBs occurring either in open and transcriptionally active euchromatin or in highly compacted and transcriptionally inactive heterochromatin [34, 35]. The chromatin compaction found in heterochromatin on one hand protects it from DNA damages but on the other hand impairs the repair process once the damage occurs. Therefore, the majority of DSBs in heterochromatin are repaired with slow kinetics through ATM-dependent mechanisms of chromatin “relaxation”, as exemplified by the ATM-mediated phosphorylation of KAP1. KAP1, the obligate co-repressor for KRAB zinc fingers, the largest family of zinc-finger transcription factors, can undergo SUMOylation. KAP1 SUMOylation mediates its interaction with NuRD complexes owing to a SIM motif found in the C-terminal part of the ATP-dependent chromatin remodeling subunit CHD3 [36]. Upon DSB induction, phosphorylation of KAP1 Ser804 produces a SIM-like domain which interferes with the KAP1-SUMO/SIM-CHD3 interaction thereby causing dispersal of CHD3/NuRD from the DSBs and chromatin relaxation allowing repair to occur [37, 38].

**Figure 10:**
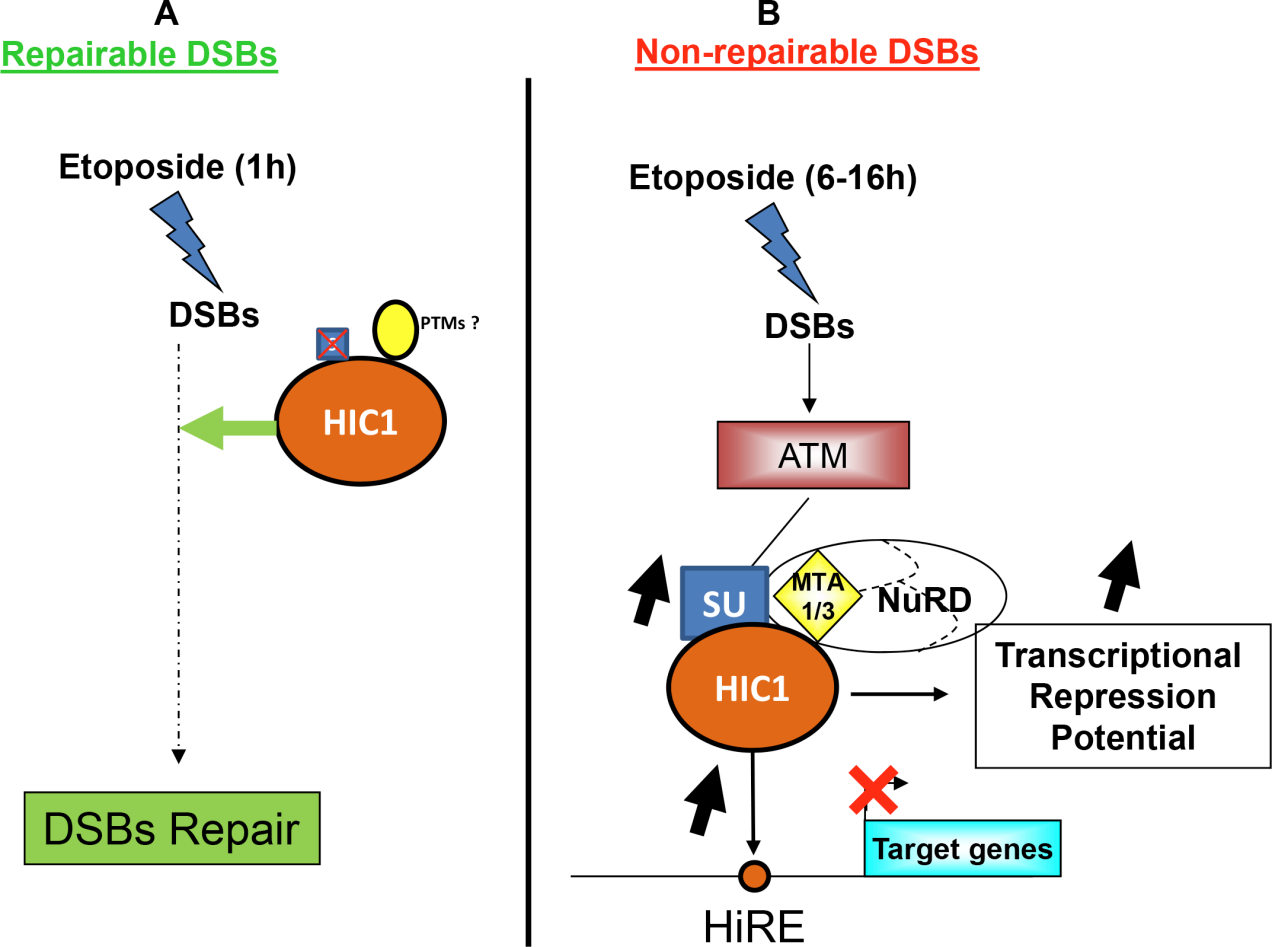
HIC1 SUMOylation is dispensable for DNA repair but essential for the transcriptional response to non-repairable DSBs (**A**) Upon induction of repairable DSBS, HIC1 participates in the early steps of the repair process [8] by a mechanism that still remains to be deciphered. However, this is independent of HIC1 SUMOylation but could be due to other post-translational modifications. (**B**) Upon induction of non-repairable DSBs, the activated ATM kinase increases HIC1 SUMOylation which in turn enhances the binding of HIC1 to its responsive elements (HiRE) in the promoters of target genes (e.g. *SIRT1*) as well as the interaction of HIC1 with the MTA1 or MTA3 co-repressors to increase the transcriptional repression of direct target genes.

In this manuscript, we have studied in-depth the SUMOylation of the transcriptional repressor HIC1 in various aspects of the DDR to DSBs. Indeed, HIC1 was originally characterized as a tumor suppressor gene encoding a transcriptional repressor facilitating the P53 dependent apoptotic response [4, 6, 5]. But HIC1 is also involved in the repair of DSBs since BJ-hTERT fibroblasts treated for 1 hour with etoposide and siRNAs targeting HIC1 repairs DSBs less efficiently than cells treated with control siRNAs [8]. Therefore, the current study nicely ties together HIC1 and the two major areas of SUMOylation research, the role of SUMOylation in transcriptional repression and its role in the DNA damage response [30, 33]. In that setting, we previously demonstrated an ATM-dependent increase in HIC1 SUMOylation favoring the interaction with MTA1, a core component of NuRD chromatin remodeling complexes in response to irreparable DSBs induced by a 16 hour etoposide treatment [8, 11]. On the contrary, HIC1 SUMOylation remains globally at basal levels when cells are exposed to etoposide for 1 hour to induce repairable damage, even in presence of the ATM inhibitor Ku-56933 (Figure 1). To date the only functions ascribed to HIC1 SUMOylation are to increase the transcriptional repression potential of a HIC1-Gal4 chimera in transient luciferase assays [10] and to enhance the interaction of HIC1 with the corepressor MTA1 [8, 11]. Even though its SUMOylation levels did not increase, HIC1 can still be SUMOylated when cells are exposed to etoposide for 1 hour and SUMOylation is one of the major post-transcriptional modifications involved in the DNA damage response [27]. However, results of the Comet assays performed with the non-SUMOylatable HIC1 E316A strongly argue against a positive role of HIC1 SUMOylation and hence of HIC1-NuRD interactions in the well documented functions for SUMOylation in DSBs repair: inhibition of local transcription at DSBs, repression of target genes during the repair process or heterochromatin relaxation (Figure 10).

In contrast to the obvious lack of necessity for the cellular response to repairable DSBs demonstrated in this study, the increase of HIC1 SUMOylation, important for the response to irreparable damage, has previously been shown to be dependent on ATM (Figure 3) [8]. We have further refined this finding by showing that this HIC1 SUMOylation increase is independent of the effector nucleoplasmic kinase, Chk2 (Figure 4). Since ATM is stabilized and activated at chromatin, these results are in strong agreement with the “on-site modification” model for SUMOylation [39]. Furthermore, we have shown that the functionally distinct MTA3 corepressor also interacts with HIC1 in a SUMOylation-dependant manner. In ChIP experiments, we also observed an increased binding of HIC1 to the *SIRT1* promoter. This could be explained at least in part by the increased SUMOylation of HIC1 since SUMOylation increased the DNA-binding properties of the BTB/POZ transcriptional repressor PLZF [23, 24] and the POU transcription factor Oct4 [40]. As a whole, these results firmly link the increase of HIC1 SUMOylation to a role in the transcriptional repression of target genes by NuRD repressive complexes to orchestrate the apoptotic DNA damage response (Figure 10). Two key genes in this process are *SIRT1* [6] and *p21* [25]. To have a more global vision of the HIC1 target genes involved in the transcriptional DNA damage response to non-repairable DSBs, we have conducted gene profiling analyses of BJ-hTERT fibroblasts with or without HIC1 inactivation by RNA interference and treated or not with etoposide for 6 hours. Through two thorough normalization strategies we were able to identify 475 genes regulated by HIC1 in an etoposide-dependent manner (Figure 9). In good agreement with the longstanding role of HIC1 in the P53-dependent apoptotic response [6] and the importance of HIC1 SUMOylation in this process as shown by our previous work [8, 10, 11] and extended in this study, many of these genes are implicated in cell death and cell cycle control. Further crossing these 475 genes with known MTA1 target genes highlighted 17 common genes, which could be HIC1 direct or indirect target genes. Among them, we validated by qRT-PCR analyses three receptors, the Ephrin A4 tyrosine kinase receptor, the GPCR receptor Latrophilin2 (LPHN2) and the TGF-beta type III receptor, a membrane proteoglycan that often functions as a co-receptor with other TGF-beta receptor superfamily members. Interestingly, we also defined as potential new HIC1 target genes TRIB2, a pro-apototic-molecule belonging to the atypical protein kinase family Tribbles [41] and SDF-1/CXCL12, a chemokine which is the ligand for the G-protein coupled chemokine (C-X-C motif) receptors, CXCR4 and CXCR7, the latter being a direct HIC1 target gene [42, 43].

In conclusion, our work demonstrates that HIC1 SUMOylation is dispensable for DNA DSB repair but essential as a transcriptional repressor for the apoptotic response in the case of irreparable DSBs. Further studies are currently in progress to decipher the functional role of HIC1 in DNA repair. In particular, we are trying to identify potential HIC1 PTMs specifically induced when cells are exposed to repairable DSBs. These analyses would help to better decipher and understand the contribution of HIC1 to the repair process.

## MATERIALS AND METHODS

### Cell lines and transfection

Cos-7, HEK293T and BJ-hTERT cells were maintained in Dulbecco modified Eagle medium (Invitrogen) supplemented with 10% fetal calf serum, non-essential amino acids and gentamycin. Cells were cultured at 37°C in humidified 5% CO^2^ atmosphere.

### Plasmids and chemicals

The expression vectors for full-length FLAG-HIC1, the non-SUMOylatable FLAG-HIC1 E316A, for His-SUMO2, Myc-MTA1, the non SUMOylatable Myc-MTA1 K509R and SIM-deficient Myc-MTA1 AAA mutants have been described previously [8, 10, 19]. The ΔMKHEP deletion mutant was generated by the two-round PCR mutagenesis strategy.

Etoposide and the Chk2 inhibitor C3742 (Chk2 inhibitor II hydrate) were purchased from Sigma-Aldrich. Wortmannin (Calbiochem), a PI3K inhibitor which also inhibits PIKKs and ATM (KU-55933; Santa Cruz Biotechnology) and DNA-PKcs (NU-7441, Selleckchem) inhibitors were dissolved in DMSO and used at a final concentration of 10 μM. Inhibitors were added to culture medium 1 hour before subsequent treatments.

### Transfection and co-immunoprecipitation

Cells were transfected in Opti-MEM (Invitrogen) by the PEI method using ExGen 500 (Euromedex) as previously described with 2.5 μg of DNA corresponding to the relevant expression vectors or the empty vector used as control. Cells were transfected for 6 h and then incubated in fresh complete medium.

For co-immunoprecipitation analyses (Co-IPs), 48 h after transfection, cells were rinsed with cold PBS and lysed in cold IPH buffer (50 mM Tris, 150 mM NaCl, 5 mM EDTA, 0.5% NP40, protease inhibitor cocktail [Roche]). Cell lysates were sonicated briefly and cleared by centrifugation (14,000 rpm, 4°C, 15 min). The supernatants were pre-cleared with 15 μl of protein A/G sepharose beads (Amersham Bioscience) incubating during 1 hour on a rotator at 4°C. Then, lysates were incubated with 2 μg of antibody on a rotator at 4°C overnight. Later, 20 μl of protein A/G beads were added and incubated 30 min at 4°C. Finally, the beads were washed three times with IPH buffer. Bound proteins were eluted by boiling in Laemmli buffer before analyses by SDS/PAGE followed by immunoblotting.

### Small interfering RNA

HEK293T cells were reverse-transfected with Lipofectamine RNAiMax (Invitrogen) according to manufacturer's instructions using 10 nM small interfering RNA targeting Chk2 (Human CHEK2 siGENOME- SMARTpool M-003256-06-0005, Dharmacon), ATM (Human ATM siGENOME siRNA-SMARTpool M-003201-04-0005, Dharmacon) or a scrambled control sequence (si Ctrl; siGENOME RISC free control siRNA, Dharmacon). For DNA-PKcs (referenced as PRKDC), we prepared a pool with four individual ON-TARGET plus PRKDC siRNAs (LQ-005030-00-0005, Dharmacon) or tested these four siRNAs individually [44]. 24 hours after siRNA transfection, cells were transfected with FLAG or FLAG-HIC1 vectors and treated with etoposide 24 hours later as previously described [8].

### Comet assays

Cos7 cells were transfected for 48 h with expression vectors either for wt FLAG-HIC1 and the empty FLAG expression vector or for the non-SUMOylatable point mutant FLAG-HIC1 E316A and analyzed by neutral Comet assays, as previously described [8, 44]. For each condition, 2,000 cells were suspended in 80μl of 0.5% low melting point agarose at 42°C. The suspension was immediately laid onto a comet slide (TREVIGEN Inc.). Agarose was allowed to solidify at 4°C for 20 min. The comet slides were then immersed in prechilled lysis solution (1.2 M NaCl, 100 mM EDTA, 10 mM Tris, 1% Triton, pH = 10) at 4°C, for 90 min in the dark. Comet slides were next placed in a horizontal electrophoresis unit, and let to equilibrate in electrophoresis buffer (Tris 89 mM, Boric acid 89 mM, EDTA 2 mM, pH8 to detect double strand breaks) for 10 min at 4°C, in the dark. After migration (40 V for 25 minutes), the slides were stained with SYBR green (Molecular Probes-1000X) according to manufacturer's recommendations.

### Quantitative RT-PCR

Total RNA was reverse transcribed using random primers and MultiScribeTM reverse transcriptase (Applied Biosystems). Real-time PCR analysis was performed by Power SYBR Green (Applied Biosystems) in a MX3005P fluorescence temperature cycler (Stratagene) according to the manufacturer's instructions. Results were normalized with respect to 18S RNA used as an internal control [45]. The primers used for the qRT-PCR analyses are summarized in Supplementary Table S4.

### Western blotting and antibodies

Proteins were separated by SDS-PAGE and transferred onto nitrocellulose membranes (GE healthcare). Western blot analyses were performed as previously described [8].

Commercial antibodies of the following specificities were used: FLAG M2 from Sigma; Anti MTA1 (sc-9445 for WB and sc-10813 for IP) from Santa Cruz and Anti MTA3 (ab87275) from Abcam; γH2AX, H2AX (total), pS1681ATM, ATM total, DNA-PKcs total, DNA-PKcs (pS2056) from Abcam, Chk2, pS516Chk2, pT68Chk2 pS20P53 and anti-actin antibodies (sc-1616-R) from Santa Cruz Biotechnology. The secondary antibodies were horseradish peroxidase-linked antibodies against rabbit, rat and mouse immunoglobulins (Amersham Biosciences); goat immunoglobulins (Southern Biotech).

To analyze the SUMOylation of HIC1 proteins by Western blotting analyses, transfected HEK293T cells pelleted by centrifugation were directly lysed in Laemmli loading buffer, boiled for 10 minutes and processed for Western blotting as described above.

### Chromatin immunoprecipitation

Human BJ-hTERT fibroblasts were treated with DMSO or Etoposide for 16 hours, washed with PBS and resuspended in 0.5 ml PBS for 5 × 10^6^ cells. Then, cells were fixed by adding formaldehyde to a final concentration of 1% for 8 min at room temperature. To stop fixation, glycine was added to a final concentration of 0.125 M. After 5 min at room temperature, cells were collected by centrifugation (1500 rpm, at 4°C, 5 min). The supernatants were removed and we lysed cells by resuspension in chilled cell lysis buffer for 10 min on a rotator at 4°C. Then, the samples were pelleted, resuspended in 200 μl nuclei lysis buffer and sonicated to chromatin with an average size of 250 bp using a cooling BioRuptor (Diagenode, Belgium). 20 μg of chromatin was immunoprecipitated with indicated antibodies and real-time PCR analyses were performed as described [22]. The primers used for *GAPDH*, *SIRT1* and *ATOH1* have been previously described [46].

### Microarrays analyses

Total RNA was prepared from BJ-hTERT human fibroblasts transfected with siCtrl or siHIC1 and mock-treated (DMSO) or treated with etoposide for 6 hours. Samples were prepared for gene expression microarrays using the Illumina^®^ TotalPrep™-96 RNA Amplification Kit (Ambion, Austin, TX). In brief, 200 ng of total RNA for each sample underwent 1^st^ and 2^nd^ strand cDNA synthesis followed by an *in vitro* transcription (IVT) amplification that generates biotinylated, antisense RNA copies of each mRNA, referred to as cRNA. For each sample, 750 ng of cRNA was hybridized to a HumanHT-12v4 BeadChip array (Illumina, Inc., San Diego, CA) for 16 hours, followed by washing, blocking, and streptavidin-Cy3 staining according to the Whole-Genome Gene Expression Direct Hybridization protocol (Illumina, Inc., San Diego, CA). The arrays were scanned using an Illumina HiScanSQ System and the resultant image files were analyzed by GenomeStudio™ Gene Expression Module (Illumina, Inc., San Diego, CA). Significance of repression was determined in a comparison context between etoposide treated and null in the HIC1 replete context using an FDR corrected *p-value* of 0.05 regardless of magnitude.

## SUPPLEMENTARY MATERIALS FIGURES AND TABLES









## References

[1] Ciccia A, Elledge SJ (2010). The DNA damage response: making it safe to play with knives. Molecular Cell.

[2] Polo SE, Jackson SP (2011). Dynamics of DNA damage response proteins at DNA breaks: a focus on protein modifications. Genes and development.

[3] Shiloh Y, Ziv Y The ATM protein kinase: regulating the cellular response to genotoxic stress, and more. Nature reviews.

[4] Wales MM, Biel MA, el Deiry W, Nelkin BD, Issa JP, Cavenee WK, Kuerbitz SJ, Baylin SB (1995). p53 activates expression of HIC-1, a new candidate tumour suppressor gene on 17p13. 3. Nature medicine.

[5] Rood BR, Leprince D (2013). Deciphering HIC1 control pathways to reveal new avenues in cancer therapeutics. Expert opinion on therapeutic targets.

[6] Chen WY, Wang DH, Yen RC, Luo J, Gu W, Baylin SB (2005). Tumor suppressor HIC1 directly regulates SIRT1 to modulate p53-dependent DNA damage responses. Cell.

[7] Dehennaut V, Leprince D (2009). Implication of HIC1 (Hypermethylated In Cancer 1) in the DNA damage response. Bulletin du cancer.

[8] Dehennaut V, Loison I, Dubuissez M, Nassour J, Abbadie C, Leprince D (2013). DNA double-strand breaks lead to activation of hypermethylated in cancer 1 (HIC1) by SUMOylation to regulate DNA repair. The Journal of biological chemistry.

[9] Fleuriel C, Touka M, Boulay G, Guerardel C, Rood BR, Leprince D (2009). HIC1 (Hypermethylated in Cancer 1) epigenetic silencing in tumors. The international journal of biochemistry and cell biology.

[10] Stankovic-Valentin N, Deltour S, Seeler J, Pinte S, Vergoten G, Guerardel C, Dejean A, Leprince D (2007). An acetylation/deacetylation-SUMOylation switch through a phylogenetically conserved psiKXEP motif in the tumor suppressor HIC1 regulates transcriptional repression activity. Molecular and cellular biology.

[11] Van Rechem C, Boulay G, Pinte S, Stankovic-Valentin N, Guerardel C, Leprince D, Differential regulation of HIC1 target genes by CtBP NuRD via an acetylation/SUMOylation switch in quiescent versus proliferating cells (2010). Molecular and cellular biology.

[12] Hay RT (2005). SUMO: a history of modification. Molecular cell.

[13] Sirbu BM, Cortez D, DNA damage response: three levels of DNA repair regulation (2013). Cold Spring Harbor perspectives in biology.

[14] Gareau JR, Lima CD (2010). The SUMO pathway: emerging mechanisms that shape specificity, conjugation and recognition. Nature reviews Mol Cell Biol.

[15] Caron P, Choudjaye J, Clouaire T, Bugler B, Daburon V, Aguirrebengoa M, Mangeat T, Iacovoni JS, Alvarez-Quilon A, Cortes-Ledesma F, Legube G (2015). Non-redundant Functions of ATM and DNA-PKcs in Response to DNA Double-Strand Breaks. Cell reports.

[16] Hill R, Lee PW (2010). The DNA-dependent protein kinase (DNA-PK): More than just a case of making ends meet?. Cell cycle.

[17] Lukas C, Falck J, Bartkova J, Bartek J, Lukas J (2003). Distinct spatiotemporal dynamics of mammalian checkpoint regulators induced by DNA damage. Nature cell biology.

[18] Hecker CM, Rabiller M, Haglund K, Bayer P, Dikic I (2006). Specification of SUMO1- and SUMO2-interacting motifs. The Journal of biological chemistry.

[19] Cong L, Pakala SB, Ohshiro K, Li DQ, Kumar R (2011). SUMOylation SUMO-interacting motif (SIM) of metastasis tumor antigen 1 (MTA1) synergistically regulate its transcriptional repressor function. The Journal of biological chemistry.

[20] Manavathi B, Singh K, Kumar R (2007). MTA family of coregulators in nuclear receptor biology and pathology. Nuclear receptor signaling.

[21] Dege C, Hagman J (2014). Mi-2/NuRD chromatin remodeling complexes regulate B and T-lymphocyte development and function. Immunological reviews.

[22] Dubuissez M, Loison I, Paget S, Vorng H, Ait-Yahia S, Rohr O, Tsicopoulos A, Leprince D (2016). “PKC-mediated phosphorylation of BCL11B at Serine 2 negatively regulates its interaction with NuRD complexes during CD4+ T cell activation”. Molecular and cellular biology.

[23] Kang SI, Chang WJ, Cho SG, Kim IY (2003). Modification of promyelocytic leukemia zinc finger protein (PLZF) by SUMO-1 conjugation regulates its transcriptional repressor activity. The Journal of biological chemistry.

[24] Chao TT, Chang CC, Shih HM (2007). SUMO modification modulates the transrepression activity of PLZF. Biochemical and biophysical research communications.

[25] Dehennaut V, Loison I, Boulay G, Van Rechem C, Leprince D (2013). Identification of p21 (CIP1/WAF1) as a direct target gene of HIC1 (Hypermethylated In Cancer 1). Biochemical and biophysical research communications.

[26] Ghanta KS, Li DQ, Eswaran J, Kumar R (2011). Gene profiling of MTA1 identifies novel gene targets and functions. PloS one.

[27] Jackson SP, Durocher D (2013). Regulation of DNA damage responses by ubiquitin and SUMO. Molecular cell.

[28] Goodarzi AA, Jeggo PA (2013). The repair and signaling responses to DNA double-strand breaks. Advances in genetics.

[29] Dantuma NP, van Attikum H (2016). Spatiotemporal regulation of posttranslational modifications in the DNA damage response. The EMBO journal.

[30] Hay RT (2013). Decoding the SUMO signal. Biochemical Society transactions.

[31] Yang SH, Jaffray E, Senthinathan B, Hay RT, Sharrocks AD (2003). SUMO and transcriptional repression: dynamic interactions between the MAP kinase and SUMO pathways. Cell cycle.

[32] Hendriks IA, Treffers LW, Verlaan-de Vries M, Olsen JV, Vertegaal AC (2015). SUMO-2 Orchestrates Chromatin Modifiers in Response to DNA Damage. Cell reports.

[33] Hendriks IA, Vertegaal AC (2015). SUMO in the DNA damage response. Oncotarget.

[34] Cann KL, Dellaire G (2011). Heterochromatin and the DNA damage response: the need to relax. Biochemistry and cell biology.

[35] Goodarzi AA, Jeggo PA, The heterochromatic barrier to DNA double strand break repair: how to get the entry visa (2012). International journal of molecular sciences.

[36] Goodarzi AA, Kurka T, Jeggo PA, Nature structural and molecular biology (2011). KAP-1 phosphorylation regulates CHD3 nucleosome remodeling during the DNA double-strand break response.

[37] Ziv Y, Bielopolski D, Galanty Y, Lukas C, Taya Y, Schultz DC, Lukas J, Bekker-Jensen S, Bartek J, Shiloh Y (2006). Chromatin relaxation in response to DNA double-strand breaks is modulated by a novel ATM- and KAP-1 dependent pathway. Nature cell biology.

[38] Goodarzi AA, Noon AT, Deckbar D, Ziv Y, Shiloh Y, Lobrich M, Jeggo PA (2008). ATM signaling facilitates repair of DNA double-strand breaks associated with heterochromatin. Molecular cell.

[39] Sarangi P, Zhao X (2015). SUMO-mediated regulation of DNA damage repair and responses. Trends in biochemical sciences.

[40] Wei F, Scholer HR, Atchison ML (2007). Sumoylation of Oct4 enhances its stability, DNA binding, and transactivation. The Journal of biological chemistry.

[41] Lin KR, Lee SF, Hung CM, Li CL, Yang-Yen HF, Yen JJ (2007). Survival factor withdrawal-induced apoptosis of TF-1 cells involves a TRB2-Mcl-1 axis-dependent pathway. The Journal of biological chemistry.

[42] Van Rechem C, Rood BR, Touka M, Pinte S, Jenal M, Guerardel C, Ramsey K, Monte D, Begue A, Tschan MP, Stephan DA, Leprince D (2009). Scavenger chemokine (CXC motif) receptor 7 (CXCR7) is a direct target gene of HIC1 (hypermethylated in cancer 1). The Journal of biological chemistry.

[43] Zheng J, Wang J, Sun X, Hao M, Ding T, Xiong D, Wang X, Zhu Y, Xiao G, Cheng G, Zhao M, Zhang J, Wang J (2013). HIC1 modulates prostate cancer progression by epigenetic modification. Clin Cancer Res.

[44] Nassour J, Martien S, Martin N, Deruy E, Tomellini E, Malaquin N, Bouali F, Sabatier L, Wernert N, Pinte S, Gilson E, Pourtier A, Pluquet O (2016). Defective DNA single-strand break repair is responsible for senescence and neoplastic escape of epithelial cells. Nature communications.

[45] Boulay G, Malaquin N, Loison I, Foveau B, Van Rechem C, Rood BR, Pourtier A, Leprince D (2012). Loss of Hypermethylated in Cancer 1 (HIC1) in breast cancer cells contributes to stress-induced migration and invasion through beta-2 adrenergic receptor (ADRB2) misregulation. The Journal of biological chemistry.

[46] Boulay G, Dubuissez M, Van Rechem C, Forget A, Helin K, Ayrault O, Leprince D (2012). Hypermethylated in cancer 1 (HIC1) recruits polycomb repressive complex 2 (PRC2) to a subset of its target genes through interaction with human polycomb-like (hPCL) proteins. The Journal of biological chemistry.

