# Supplementary material for: HIC1 (hypermethylated in cancer 1) SUMOylation is dispensable for DNA repair but is essential for the apoptotic DNA damage response (DDR) to irreparable DNA double-strand breaks (DSBs)
Source: Oncotarget. 2016 Dec 7;8(2):2916–35. doi: 10.18632/oncotarget.13807 (PMC5356852; doi:10.18632/oncotarget.13807)
Supplement: Supplementary file 1 [file oncotarget-08-2916-s001.pdf]

HIC1 (hypermethylated in cancer 1) SUMOylation is dispensable for DNA repair but is essential for the apoptotic DNA damage response (DDR) to irreparable DNA double-strand breaks (DSBs)

Supplementary Materials

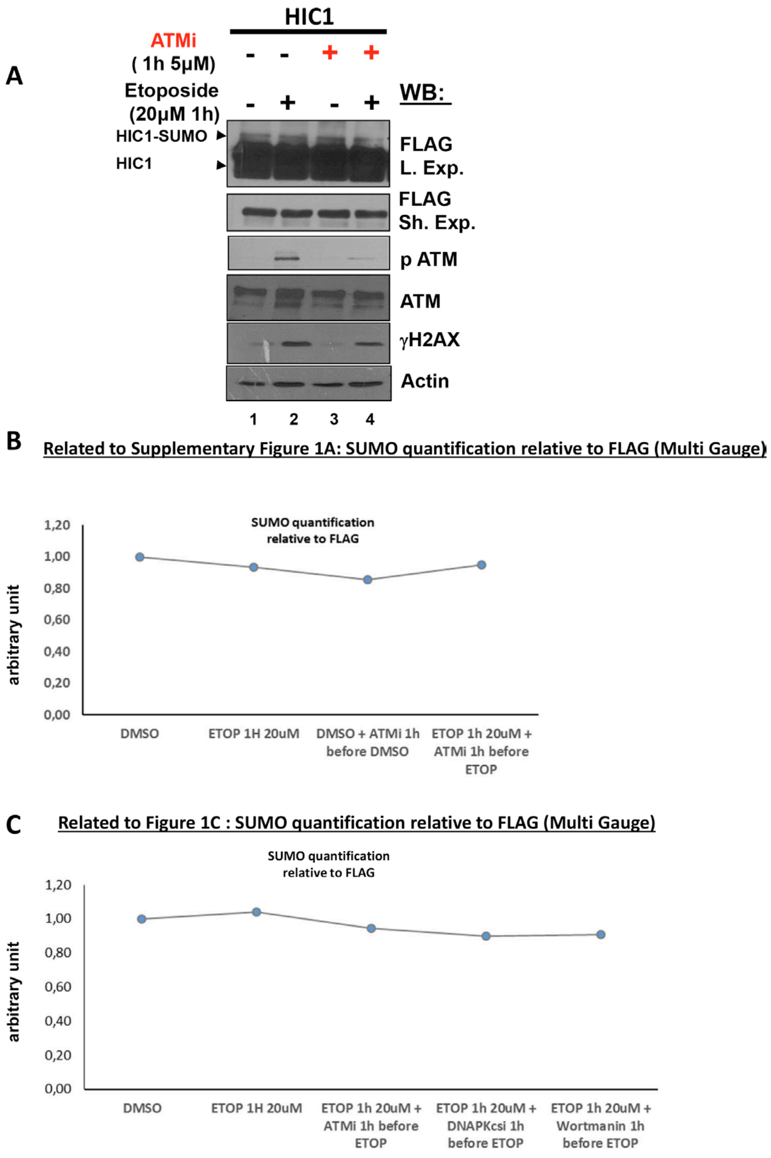

Supplementary Figure S1: Characterisation of HIC1 SUMOylation upon induction of repairable DSBs (1 hour etoposide treatment) in absence or in presence of an ATM inhibitor. (**A**) HEK 293T cells were transfected with FLAG-HIC1 and treated with etoposide or DMSO for 1 hour. Transfected cells were incubated with or without 5 µM of an ATM specific inhibitor (ATMi: KU-55933 also dissolved in DMSO) 1 hour before etoposide treatment, as indicated. Cell extracts were prepared by direct lysis in denaturing conditions and analyzed by immunoblotting using the indicated antibodies. The decrease of ATM autophosphorylation on Serine 1681 and of γH2Ax confirms the effectiveness of the ATM inhibitor treatment (Lane 4). (**B**) Quantification of SUMO-HIC1 to total HIC1 (FLAG) for the experiments presented in the above panel A) was performed with the Fujifilm MultiGauge software. (**C**) Quantification of SUMO-HIC1 to total HIC1 (FLAG) for the experiment presented in Figure 1, panel C) in the main manuscript was performed with the Fujifilm MultiGauge software.

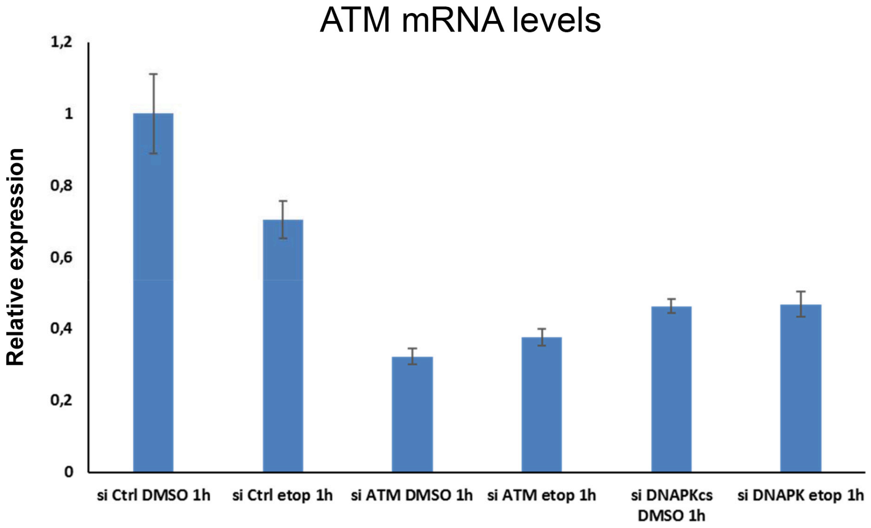

Supplementary Figure S2: RT-qPCR analyses of ATM expression levels in HEK293T cells treated with etoposide for 16 hours after transfection with HIC1 and the indicated siRNAs. HEK293T cells were transfected either with nontargeted control siRNA (siCrtl), either with a pool of four siRNAs targeting ATM (siATM) or with a pool of four siRNAs targeting DNAPKcs (siDNAPKcs). The next day, these cells were transfected with a FLAG-HIC1 expression vector for 24 hours and were then treated with 20 µM etpoposide (+) or mock-treated with DMSO (-) as control for 16 hours. Total RNAs were extracted and RT-qPCR experiments were performed to analyse the expression level of *ATM*.

A **Related to Revised Figure 4A : SUMO quantification relative to FLAG (Multi Gauge)**

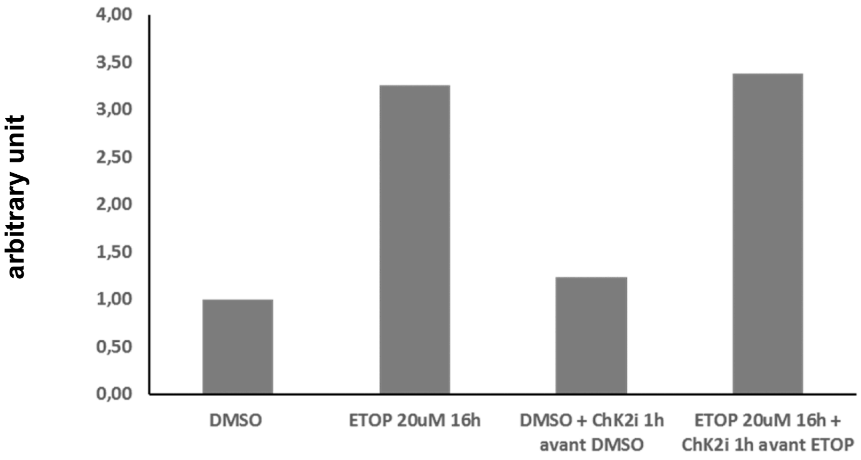

B **Related to Revised Figure 4C : SUMO quantification relative to FLAG (Multi Gauge)**

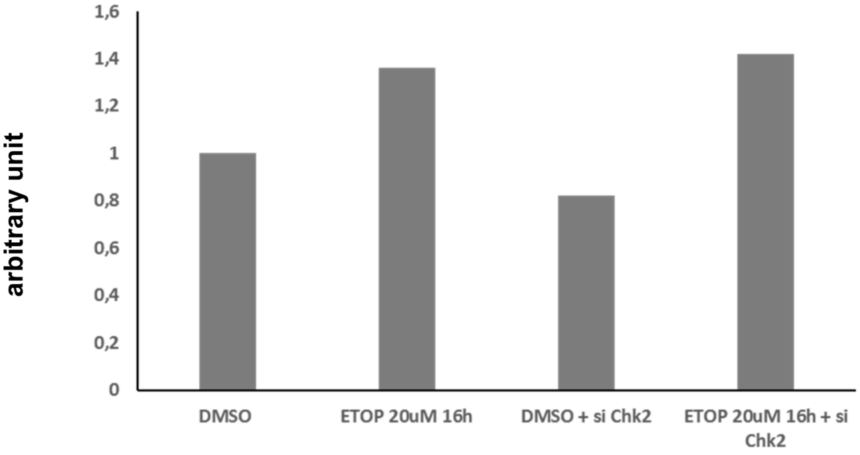

Supplementary Figure S3: Characterisation of HIC1 SUMOylation upon induction of irreparable DSBs (16 hours etoposide treatment) after inactivation of Chk2 by pharmacological inhibitors. Quantification of SUMO-HIC1 to total HIC1 (FLAG) for the experiments performed in presence of Chk2i inhibitor and presented in the manuscript as Figure 4A, lanes 5 to 8, was performed with the Fujifilm MultiGauge software.

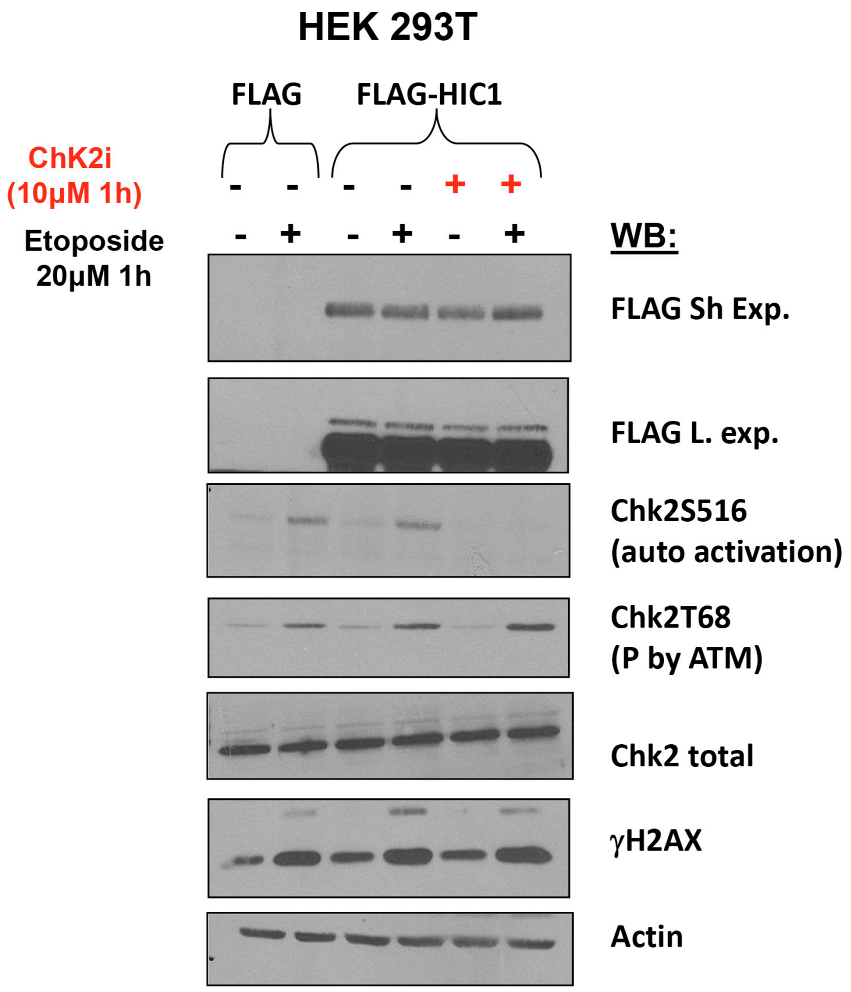

Supplementary Figure S4: The Chk2 inhibitor does not affect HIC1 SUMOylation levels in the presence of repairable DNA double-strand breaks (DSBs) induced by a 1 hour etoposide treatment. HEK293T cells were transfected with the FLAG and FLAG-HIC1 vectors. 48 hours after transfection, cells were pre-incubated or not with the Chk2 inhibitor (Chk2i) for 1 hour and then with etoposide for 1 hour as indicated. Cell extracts were prepared and Western blotting was performed with the indicated antibodies. γH2AX and actin levels were used as controls for DSB induction and equal loading, respectively.

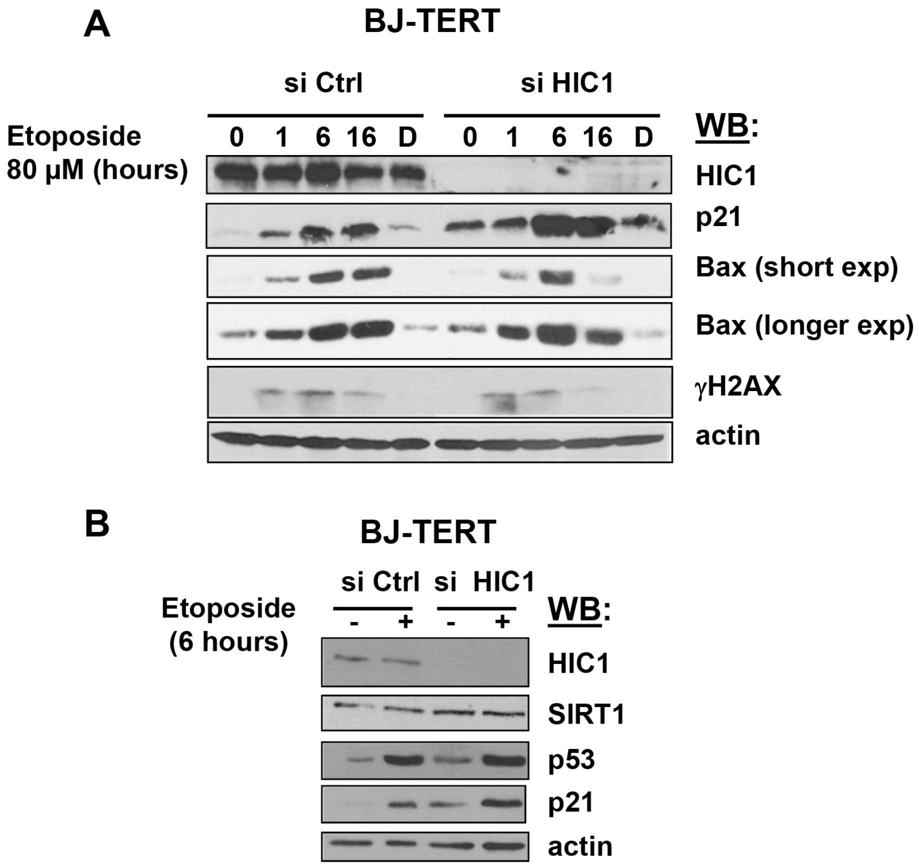

Supplementary Figure S5: A 6 hours etoposide treatment is sufficient to induce p21^{CIP1} expression in BJ-TERT fibroblasts. (**A**) Increased expression of P21^{CIP1} after various etoposide treatments. BJ-hTERT fibroblasts were transfected with control siRNAs (siCtrl) or siRNAs targeting HIC1 (siHIC1) for 48 hours before the induction of DSBs by 80 µM etoposide treatments of either 1 hour to induce repairable DSBs, of 16 hours to induce a full P53 apoptotic response (Chen et al., 2005) or of 6 hours, as an intermediate time to favor the induction of HIC1 direct target genes whilst limiting the induction of P53 direct target genes. D corresponds to cell treated for 16 hours with vehicle (DMSO) alone. Total cell extracts were analyzed by immunoblotting with the indicated antibodies. (**B**) Validation of the cell populations used in the microarrays analyses. BJ-hTERT fibroblasts (× 100 mm plates) were transfected with siCrtl or siHIC1 and treated for 6 hours with DMSO (-) or etoposide. Total RNAs were extracted for gene expression profiling experiments. As control, aliquots of cells in each condition were directly lysed in Laemmli buffer and analyzed by Western blotting with the indicated antibodies.

Supplementary Table S1: Normalization strategy #1 comparing etoposide vs si-cntrl decreased and si-HIC-etoposide vs si-HIC decreased genes-629 genes. See Supplementary_Table_S1

Supplementary Table S2: Normalization strategy #2 comparing Etop vs si-cntrl decreased and si-HIC-Etop vs si-cntrl decreased genes-475 genes. See Supplementary_Table_S2

Supplementary Table S3: Intersection of normalization strategies 1&2-319 genes.
See Supplementary_Table_S3

Supplementary Table S4: Sequences of Primers for RT-qPCR

**CHK2** forward	CCCCTGGGCTCGATTATG
**CHK2** reverse	TCCCTCCCAAACCAGTAGTTGT
**ATM** forward	AGATGATGGGAGGCCTAGGA
**ATM** reverse	ACAAAGTGTGATGGGGGTGA
**HIC1** forward	CGACGACTACAAGAGCAGCAGC
**HIC1** reverse	CAGGTTGTCACCGAAGCTCTC
**SIRT1** forward	GCGGGAATCCAAAGGATAAT
**SIRT1** reverse	CACCTAGGACATCGAGGAACTACC
**P21** forward	GACTCTCAGGGTCGAAAACG
**P21** reverse	GGCTTCCTCTTGGAGAAGATCA
**CXCL12** forward	AACGCCAAGGTCGTGGTC
**CXCL12** reverse	GCATGGGCATCTGTAGCTCA
**EPHA4** forward	ACACGAAAGGGGACCTGGCA
**EPHA4** reverse	CGCATTGCCTGGACACTGCTCA
**LPHN2** forward	CTCTGGGCATCAAAGGAGCA
**LPHN2** reverse	CTTTGCAAGCCCATTCCTGC
**TGFβR3** forward	ACCGTGATGGGCATTGCGTTTGCA
**TGFβR3** reverse	GTGCTCTGCGTGCTGCCGATGCTGT
**TRIB2** forward	AGGACGAAGAGAGGACTCGG
**TRIB2** reverse	CATGCTTGTCGGAGAGGGAA

Sequences of Primers used in the study are provided in the following table.
