# Supplementary material for: HIC1 (hypermethylated in cancer 1) SUMOylation is dispensable for DNA repair but is essential for the apoptotic DNA damage response (DDR) to irreparable DNA double-strand breaks (DSBs)
Source: Oncotarget. 2016 Dec 7;8(2):2916–35. doi: 10.18632/oncotarget.13807 (PMC5356852; doi:10.18632/oncotarget.13807)
Supplement: Supplementary file 2 [file oncotarget-08-2916-s002.doc]

| **Supplemental Table 1: Normalization strategy #1 comparing etoposide vs si-cntrl decreased and si-HIC-etoposide vs si-HIC decreased genes - 629 genes** | | | | |
| --- | --- | --- | --- | --- |
|  | | | | |
| PROBE_ID | p-value  (Etop vs. ctr-null) | Fold-Change  (Etop vs. ctr-null) | SYMBOL | |
| ILMN_1829845 | 0.001324 | -1.28 |  |  |
| ILMN_1704842 | 0.001165 | -1.61 | ARL4A |  |
| ILMN_1701643 | 0.001711 | -1.34 | GDPD5 |  |
| ILMN_1685339 | 1.24E-05 | -1.78 | TPM1 |  |
| ILMN_1721344 | 0.000422 | -1.21 | MOBKL2A |  |
| ILMN_2396697 | 0.002329 | -1.32 | GRIK2 |  |
| ILMN_1741477 | 0.001112 | -1.20 | SMAD4 |  |
| ILMN_1772522 | 0.00317 | -1.26 | ZFP161 |  |
| ILMN_1783815 | 0.000764 | -1.33 | COG7 |  |
| ILMN_1683158 | 0.001594 | -1.61 | LOC441440 |  |
| ILMN_1678075 | 0.00082 | -1.45 | CDYL |  |
| ILMN_2372200 | 0.000124 | -1.37 | ZNF586 |  |
| ILMN_1745223 | 0.000189 | -1.20 | CDC42EP4 |  |
| ILMN_1676728 | 0.000609 | -1.30 | DLK2 |  |
| ILMN_1697639 | 0.000733 | -1.57 | OGT |  |
| ILMN_1738491 | 0.002007 | -1.38 | SNX30 |  |
| ILMN_1804652 | 0.000629 | -1.35 | PLEKHH3 |  |
| ILMN_1662038 | 9.22E-06 | -1.65 | LARGE |  |
| ILMN_2078124 | 0.001482 | -1.50 | FMO6P |  |
| ILMN_3241665 | 0.002228 | -1.36 | C1orf133 |  |
| ILMN_1675387 | 0.000746 | -1.23 | LIMS1 |  |
| ILMN_1788237 | 0.001082 | -1.60 | LOC652755 |  |
| ILMN_1705114 | 0.002819 | -1.28 | NUMB |  |
| ILMN_1847822 | 0.001812 | -1.24 | KIAA0368 |  |
| ILMN_2232157 | 0.003253 | -1.22 | SLMO1 |  |
| ILMN_1778681 | 0.000588 | -1.79 | EBF1 |  |
| ILMN_2066124 | 0.001608 | -1.17 | AFG3L2 |  |
| ILMN_1695020 | 0.000279 | -1.40 | NEK3 |  |
| ILMN_1754234 | 0.000894 | -1.18 | ZMYND11 |  |
| ILMN_1655748 | 0.002852 | -1.68 | ZNF323 |  |
| ILMN_1700379 | 0.001405 | -1.60 | SLC26A1 |  |
| ILMN_3242586 | 0.000149 | -1.37 | RHOU |  |
| ILMN_1728218 | 2.73E-05 | -1.95 | RBPMS |  |
| ILMN_3224204 | 0.001047 | -1.34 | PSMG4 |  |
| ILMN_1741475 | 0.001574 | -1.43 | C7orf47 |  |
| ILMN_1808374 | 0.000603 | -1.53 | SNTB2 |  |
| ILMN_1811592 | 0.000315 | -1.51 | ARHGAP21 |  |
| ILMN_1800267 | 0.002704 | -1.46 | FAM13A |  |
| ILMN_1727574 | 0.000423 | -1.29 | ZNF827 |  |
| ILMN_1658560 | 0.00101 | -1.40 | LOC653210 |  |
| ILMN_1758658 | 0.002079 | -1.13 | FADD |  |
| ILMN_1887174 | 0.001102 | -1.26 | KIAA0146 |  |
| ILMN_1739805 | 0.002276 | -1.49 | NDE1 |  |
| ILMN_1771964 | 0.001494 | -1.24 | GSTA4 |  |
| ILMN_1654690 | 0.00307 | -1.27 | CECR5 |  |
| ILMN_1755023 | 0.001005 | -1.26 | RAD50 |  |
| ILMN_2177090 | 0.001213 | -1.20 | LOC200030 |  |
| ILMN_1760245 | 8.99E-05 | -1.27 | TMEM42 |  |
| ILMN_2364828 | 0.000295 | -1.48 | OGT |  |
| ILMN_2071809 | 2.02E-05 | -1.42 | MGP |  |
| ILMN_2410864 | 0.000911 | -1.31 | RAB28 |  |
| ILMN_1847494 | 0.001036 | -1.26 |  |  |
| ILMN_1724811 | 0.000752 | -1.56 | PARN |  |
| ILMN_1766200 | 0.000711 | -1.47 | CALHM2 |  |
| ILMN_1719661 | 1.27E-05 | -1.20 | SEPX1 |  |
| ILMN_2053415 | 0.00085 | -1.39 | LDLR |  |
| ILMN_2073184 | 0.001704 | -1.37 | S1PR5 |  |
| ILMN_1660199 | 0.002096 | -1.20 | ACAA2 |  |
| ILMN_1718866 | 4.06E-05 | -1.25 | C5orf46 |  |
| ILMN_1753426 | 0.00204 | -1.26 | KIAA0556 |  |
| ILMN_1794534 | 0.000971 | -1.63 | CCDC81 |  |
| ILMN_2339266 | 0.001044 | -1.29 | LAMA2 |  |
| ILMN_2295518 | 0.000678 | -1.30 | TRO |  |
| ILMN_2313821 | 0.000194 | -1.26 | AIFM1 |  |
| ILMN_2180519 | 0.002319 | -1.27 | LOC729603 |  |
| ILMN_1719039 | 0.002792 | -1.31 | UBE2G1 |  |
| ILMN_1665357 | 0.001325 | -1.41 | EPS15 |  |
| ILMN_2315964 | 0.001753 | -1.64 | PSRC1 |  |
| ILMN_1770758 | 0.001667 | -1.70 | AKAP6 |  |
| ILMN_2087702 | 0.00322 | -1.26 | MYH9 |  |
| ILMN_3273854 | 0.001999 | -1.24 | HNRNPA2B1 |  |
| ILMN_1683059 | 0.000715 | -1.30 | SIRT5 |  |
| ILMN_3238709 | 0.00238 | -1.61 | LOC100134068 |  |
| ILMN_2200636 | 0.000124 | -1.53 | KIAA1267 |  |
| ILMN_2374687 | 0.000172 | -1.46 | PTPN13 |  |
| ILMN_1741156 | 0.001934 | -1.38 | ARMCX5 |  |
| ILMN_2048811 | 5.86E-06 | -1.21 | NUBPL |  |
| ILMN_2338038 | 3.70E-05 | -1.41 | AK3L1 |  |
| ILMN_1810423 | 0.003234 | -1.26 | RPP40 |  |
| ILMN_1658494 | 0.000302 | -1.31 | C13orf15 |  |
| ILMN_3249006 | 0.002237 | -1.47 | LOC100133888 |  |
| ILMN_1754562 | 8.51E-06 | -1.31 | ARHGEF17 |  |
| ILMN_2382990 | 0.00105 | -1.29 | HK1 |  |
| ILMN_1689318 | 4.42E-05 | -1.87 | NUAK1 |  |
| ILMN_1671911 | 0.002033 | -1.15 | MTA1 |  |
| ILMN_1808404 | 0.001151 | -1.16 | RHBDF1 |  |
| ILMN_1817234 | 0.002684 | -1.53 |  |  |
| ILMN_2389347 | 0.000786 | -1.43 | NR3C1 |  |
| ILMN_1713178 | 0.000727 | -1.29 | FAM116A |  |
| ILMN_1904054 | 2.99E-05 | -1.67 |  |  |
| ILMN_1811551 | 0.001482 | -1.21 | DERA |  |
| ILMN_1651343 | 0.000264 | -1.45 | ITGA11 |  |
| ILMN_1652929 | 0.000799 | -1.28 | POGZ |  |
| ILMN_1780598 | 0.001917 | -1.26 | PIAS1 |  |
| ILMN_1678235 | 1.66E-05 | -1.53 | KIAA1267 |  |
| ILMN_1687375 | 3.49E-05 | -1.61 | ATP2A2 |  |
| ILMN_1719570 | 0.001707 | -1.67 | BICC1 |  |
| ILMN_1775677 | 0.002916 | -1.31 | TYSND1 |  |
| ILMN_1756408 | 0.000435 | -1.19 | PARVA |  |
| ILMN_1781174 | 0.002387 | -1.38 | KIAA1009 |  |
| ILMN_2159290 | 0.001221 | -1.55 | LOC441376 |  |
| ILMN_3256325 | 0.003256 | -1.14 | CYB561D1 |  |
| ILMN_1733851 | 0.002382 | -1.16 | DACT3 |  |
| ILMN_1822775 | 0.003158 | -1.35 |  |  |
| ILMN_1690282 | 0.001623 | -1.16 | CRADD |  |
| ILMN_1768311 | 0.000266 | -1.19 | LOC728888 |  |
| ILMN_2361575 | 0.001634 | -1.41 | SNX14 |  |
| ILMN_1789558 | 0.001496 | -1.32 | FAM164A |  |
| ILMN_1787815 | 8.15E-06 | -1.50 | TRIB3 |  |
| ILMN_3298167 | 0.002074 | -1.25 | ZSWIM7 |  |
| ILMN_1736112 | 0.000175 | -1.47 | ARHGAP10 |  |
| ILMN_2203463 | 0.00097 | -1.19 | SPATS2 |  |
| ILMN_1686392 | 0.001022 | -1.31 | NBAS |  |
| ILMN_3307892 | 0.002433 | -1.19 | PARVA |  |
| ILMN_2227968 | 0.001074 | -1.39 | NTHL1 |  |
| ILMN_1736327 | 0.00036 | -1.67 | CDC42EP3 |  |
| ILMN_2350183 | 0.00077 | -1.56 | ST5 |  |
| ILMN_2285112 | 0.000928 | -1.36 | FBXO11 |  |
| ILMN_1665049 | 0.000288 | -1.31 | SPG11 |  |
| ILMN_1771019 | 0.000946 | -1.36 | MTMR4 |  |
| ILMN_2374293 | 9.22E-05 | -1.46 | DYRK1A |  |
| ILMN_1689908 | 0.002342 | -1.19 | ANKRD13A |  |
| ILMN_1729596 | 0.000151 | -1.41 | INF2 |  |
| ILMN_2278152 | 0.000369 | -1.88 | TPM1 |  |
| ILMN_1816244 | 0.000106 | -1.54 |  |  |
| ILMN_1773940 | 0.000353 | -1.34 | GPR161 |  |
| ILMN_1768117 | 0.002743 | -1.32 | RBM25 |  |
| ILMN_1880446 | 0.000857 | -1.46 |  |  |
| ILMN_1745946 | 0.000577 | -1.39 | CCDC5 |  |
| ILMN_1695233 | 7.04E-05 | -1.99 | SHOX |  |
| ILMN_1679929 | 0.001653 | -1.39 | KLF13 |  |
| ILMN_1764177 | 7.33E-05 | -1.56 | JARID2 |  |
| ILMN_1680856 | 0.000288 | -1.69 | MAMLD1 |  |
| ILMN_1766157 | 0.001572 | -1.76 | MRVI1 |  |
| ILMN_2094061 | 0.000645 | -1.41 | IMPA2 |  |
| ILMN_2375830 | 0.001883 | -1.30 | DIXDC1 |  |
| ILMN_1667932 | 0.001043 | -1.46 | LOC652726 |  |
| ILMN_1742827 | 0.001231 | -1.29 | EXOC4 |  |
| ILMN_1798581 | 0.001274 | -1.17 | MCM8 |  |
| ILMN_1856315 | 0.000126 | -1.63 |  |  |
| ILMN_1745607 | 0.000369 | -1.34 | A2M |  |
| ILMN_3282285 | 0.002178 | -1.35 | LOC151457 |  |
| ILMN_1744647 | 0.002457 | -1.28 | CAND1 |  |
| ILMN_2075603 | 0.000452 | -1.45 | MRGPRF |  |
| ILMN_2055310 | 0.001046 | -1.21 | MBD4 |  |
| ILMN_1726459 | 0.000847 | -1.41 | NPAS4 |  |
| ILMN_1804150 | 0.001127 | -1.31 | HIBADH |  |
| ILMN_2136455 | 0.001887 | -1.24 | C3orf64 |  |
| ILMN_2340259 | 0.00218 | -1.29 | PDE4B |  |
| ILMN_3282829 | 0.00315 | -1.36 | LOC727913 |  |
| ILMN_2093343 | 6.60E-05 | -1.56 | PLAC8 |  |
| ILMN_1706677 | 0.000879 | -1.32 | MAPK1 |  |
| ILMN_1708025 | 0.002745 | -1.72 | RBPMS |  |
| ILMN_1733415 | 1.72E-05 | -1.29 | MFAP5 |  |
| ILMN_1752669 | 0.000994 | -1.83 | ALPI |  |
| ILMN_2396272 | 0.000328 | -1.33 | PDCD4 |  |
| ILMN_1753639 | 0.003204 | -1.26 | MTAP |  |
| ILMN_1808245 | 2.02E-05 | -1.45 | RPESP |  |
| ILMN_1793290 | 0.001431 | -1.39 | WDR60 |  |
| ILMN_3255389 | 0.003052 | -1.27 | LOC100049716 |  |
| ILMN_2401927 | 0.002036 | -1.23 | TTC8 |  |
| ILMN_1844692 | 0.001522 | -1.21 | FOXO3 |  |
| ILMN_2318811 | 0.002595 | -1.14 | RANBP3 |  |
| ILMN_1660663 | 0.000433 | -1.69 | DYRK1A |  |
| ILMN_1658639 | 0.000111 | -1.42 | SLC46A3 |  |
| ILMN_3240222 | 0.000106 | -1.27 | PRAGMIN |  |
| ILMN_2410421 | 0.002046 | -1.23 | NBPF1 |  |
| ILMN_1669696 | 0.000326 | -1.62 | ZNF792 |  |
| ILMN_2391750 | 0.003044 | -1.48 | SFMBT1 |  |
| ILMN_2326675 | 5.88E-05 | -1.42 | NR2C1 |  |
| ILMN_1819783 | 0.001097 | -1.47 |  |  |
| ILMN_1728521 | 0.000311 | -1.68 | HDAC7A |  |
| ILMN_1746029 | 0.000784 | -1.29 | SPATS2 |  |
| ILMN_1806667 | 0.001395 | -1.14 | FRAS1 |  |
| ILMN_1725534 | 0.002203 | -1.22 | ACTN4 |  |
| ILMN_2186369 | 5.56E-05 | -1.44 | NCOR1 |  |
| ILMN_1664177 | 9.28E-05 | -1.72 | ATXN7L2 |  |
| ILMN_1703980 | 0.000766 | -1.50 | MAB21L1 |  |
| ILMN_1768751 | 0.0008 | -1.44 | MTA3 |  |
| ILMN_1654488 | 0.000373 | -1.39 | UTX |  |
| ILMN_3225534 | 0.001408 | -1.38 | RNF216L |  |
| ILMN_1699703 | 0.000172 | -1.20 | ARCN1 |  |
| ILMN_1664912 | 0.002542 | -1.15 | IL11RA |  |
| ILMN_1708805 | 0.000264 | -1.48 | NCOA3 |  |
| ILMN_1776334 | 0.000546 | -1.36 | UXS1 |  |
| ILMN_1720476 | 0.001057 | -1.22 | PHF2 |  |
| ILMN_3176746 | 0.002146 | -1.49 | LOC100128191 |  |
| ILMN_2315979 | 2.22E-05 | -1.53 | LBH |  |
| ILMN_1739582 | 0.000412 | -1.36 | HOXA9 |  |
| ILMN_1770673 | 0.000175 | -1.41 | AKNA |  |
| ILMN_1711627 | 0.000149 | -1.26 | SIAH1 |  |
| ILMN_3266964 | 0.003262 | -1.53 | LOC100128191 |  |
| ILMN_1784554 | 0.001742 | -1.32 | LOC647389 |  |
| ILMN_2288070 | 0.000768 | -1.40 | FTO |  |
| ILMN_3300797 | 0.001534 | -1.33 | LOC729090 |  |
| ILMN_2097793 | 5.43E-05 | -1.34 | ZBTB4 |  |
| ILMN_1767556 | 0.000305 | -1.91 | C10orf10 |  |
| ILMN_1760338 | 0.001247 | -1.10 | LOC643357 |  |
| ILMN_1684045 | 0.001055 | -1.39 | CDCA4 |  |
| ILMN_1728605 | 0.001101 | -1.25 | TTC3 |  |
| ILMN_1685856 | 0.000822 | -1.34 | FAM92A1 |  |
| ILMN_1733756 | 0.000106 | -1.27 | COL12A1 |  |
| ILMN_1764770 | 0.002911 | -1.39 | MGC15763 |  |
| ILMN_1732575 | 0.002848 | -1.20 | SEC14L1 |  |
| ILMN_3184894 | 0.00115 | -1.41 | LOC100127971 |  |
| ILMN_1766798 | 0.000305 | -1.35 | CENTB2 |  |
| ILMN_2401641 | 0.001093 | -1.40 | ALDH3A2 |  |
| ILMN_1739942 | 0.00023 | -1.46 | FAM117B |  |
| ILMN_3251728 | 0.002625 | -1.14 | MTMR10 |  |
| ILMN_1673798 | 0.002484 | -1.26 | PPOX |  |
| ILMN_1706990 | 5.11E-05 | -1.32 | ZNF271 |  |
| ILMN_2113490 | 0.000279 | -1.29 | NTN4 |  |
| ILMN_1661599 | 4.62E-06 | -1.68 | DDIT4 |  |
| ILMN_1784287 | 3.85E-05 | -1.39 | TGFBR3 |  |
| ILMN_1680867 | 0.001459 | -1.26 | C6orf61 |  |
| ILMN_1666122 | 4.16E-05 | -1.74 | HEG1 |  |
| ILMN_1771688 | 0.000141 | -1.35 | RAB7B |  |
| ILMN_1746720 | 0.000428 | -1.45 | TTC39C |  |
| ILMN_1681304 | 0.000465 | -1.36 | PAN3 |  |
| ILMN_1775486 | 0.002749 | -1.22 | SSPN |  |
| ILMN_1672547 | 0.001846 | -1.47 | MYO9B |  |
| ILMN_2313856 | 0.000837 | -1.42 | OSBPL9 |  |
| ILMN_1761058 | 1.96E-05 | -1.21 | ACAD11 |  |
| ILMN_1801387 | 0.001232 | -1.52 | YEATS4 |  |
| ILMN_1670172 | 0.000468 | -1.20 | WDR33 |  |
| ILMN_1656927 | 0.000112 | -1.47 | SEMA5A |  |
| ILMN_1671661 | 0.000203 | -1.18 | HSD17B7 |  |
| ILMN_1748719 | 0.000586 | -1.68 | SEC16B |  |
| ILMN_1793410 | 0.000477 | -1.48 | SNTB1 |  |
| ILMN_2311278 | 0.000383 | -1.41 | ADD3 |  |
| ILMN_1660451 | 0.00028 | -1.95 | STARD13 |  |
| ILMN_1689456 | 9.48E-05 | -1.39 | ZBTB20 |  |
| ILMN_1678215 | 0.000944 | -1.41 | RHOJ |  |
| ILMN_1678494 | 0.001434 | -1.35 | ZNF438 |  |
| ILMN_3239343 | 6.79E-05 | -1.64 | STAG3L3 |  |
| ILMN_3289685 | 0.001893 | -1.26 | LOC645452 |  |
| ILMN_1798841 | 0.000991 | -1.60 | PLCXD3 |  |
| ILMN_1775926 | 0.002851 | -1.26 | SPATA6 |  |
| ILMN_2047599 | 0.001515 | -1.15 | TMEM50B |  |
| ILMN_2384513 | 0.001581 | -1.30 | C2CD2 |  |
| ILMN_2394571 | 0.000449 | -1.31 | FBXW11 |  |
| ILMN_3227321 | 0.000319 | -1.29 | LOC731542 |  |
| ILMN_1755937 | 5.91E-05 | -1.40 | ANXA2 |  |
| ILMN_1759766 | 0.000253 | -1.23 | CTXN1 |  |
| ILMN_1657993 | 0.001232 | -1.27 | ADNP |  |
| ILMN_1765044 | 0.0005 | -1.38 | CUTC |  |
| ILMN_2215545 | 0.001984 | -1.49 | C3orf26 |  |
| ILMN_1803005 | 0.002736 | -1.29 | MMACHC |  |
| ILMN_1702933 | 0.00291 | -1.26 | ADM2 |  |
| ILMN_1752159 | 0.001863 | -1.33 | AHNAK |  |
| ILMN_1791375 | 0.000514 | -1.54 | STAG3L2 |  |
| ILMN_1739454 | 0.000225 | -1.37 | USP34 |  |
| ILMN_2179778 | 0.000828 | -1.44 | PHLDB2 |  |
| ILMN_1669657 | 0.000757 | -1.48 | LOC440345 |  |
| ILMN_1698001 | 0.001359 | -1.26 | NCK1 |  |
| ILMN_1771728 | 0.001355 | -1.33 | PXMP4 |  |
| ILMN_2412281 | 0.001029 | -1.55 | PDLIM5 |  |
| ILMN_1900110 | 1.03E-05 | -1.44 |  |  |
| ILMN_1764704 | 0.000536 | -1.46 | FAM169A |  |
| ILMN_1692026 | 0.00191 | -1.34 | SUV420H1 |  |
| ILMN_1676804 | 0.001688 | -1.50 | LOC653145 |  |
| ILMN_1790881 | 0.000253 | -1.30 | HNMT |  |
| ILMN_3240698 | 0.002221 | -1.93 | LOC388279 |  |
| ILMN_1804854 | 0.00155 | -1.15 | CTNNA1 |  |
| ILMN_1813834 | 0.001068 | -1.21 | PRMT6 |  |
| ILMN_1754220 | 0.003181 | -1.26 | SF3A2 |  |
| ILMN_1655307 | 0.002303 | -1.29 | FAM136A |  |
| ILMN_2095840 | 0.002043 | -1.26 | MYST3 |  |
| ILMN_3226663 | 0.000436 | -1.28 | MGC26356 |  |
| ILMN_1694923 | 5.54E-05 | -1.43 | PTPN9 |  |
| ILMN_3245194 | 0.000274 | -1.50 | LOC100132323 |  |
| ILMN_2335669 | 0.00084 | -1.33 | ZC3H14 |  |
| ILMN_3307221 | 0.0003 | -1.63 | NAV2 |  |
| ILMN_1762308 | 0.00037 | -1.72 | LOC654191 |  |
| ILMN_1688780 | 0.002548 | -1.20 | S100A4 |  |
| ILMN_1695972 | 0.001268 | -1.65 | CCDC89 |  |
| ILMN_1758915 | 0.00024 | -1.40 | PDCD2 |  |
| ILMN_3261226 | 0.000865 | -1.39 | C6orf186 |  |
| ILMN_1681590 | 0.001986 | -1.24 | LARP1 |  |
| ILMN_1779639 | 0.000975 | -1.39 | IRAK1BP1 |  |
| ILMN_1730931 | 0.001181 | -1.35 | RUFY3 |  |
| ILMN_2251375 | 0.001392 | -1.31 | ZFP64 |  |
| ILMN_1691570 | 0.00012 | -1.33 | METTL5 |  |
| ILMN_1808587 | 2.80E-05 | -1.68 | ZFHX3 |  |
| ILMN_1766054 | 0.000495 | -1.34 | ABCA1 |  |
| ILMN_1688630 | 2.68E-05 | -1.31 | RECK |  |
| ILMN_2398039 | 0.001688 | -1.45 | TCERG1 |  |
| ILMN_1785795 | 0.00215 | -1.23 | METAP1 |  |
| ILMN_1784985 | 0.001557 | -1.34 | PRRT3 |  |
| ILMN_2319424 | 0.00022 | -1.73 | GYG2 |  |
| ILMN_1723124 | 0.00093 | -1.33 | GALK2 |  |
| ILMN_1742935 | 0.001799 | -1.38 | ZNF33B |  |
| ILMN_1691428 | 0.000655 | -1.22 | PSMD12 |  |
| ILMN_1738099 | 0.000762 | -1.18 | C2orf34 |  |
| ILMN_1656840 | 0.001682 | -1.40 | VPS13D |  |
| ILMN_1715526 | 0.001242 | -1.33 | ZDHHC21 |  |
| ILMN_2150294 | 0.000891 | -1.21 | FKBP14 |  |
| ILMN_1790689 | 0.001937 | -1.28 | CRISPLD2 |  |
| ILMN_1664772 | 0.002435 | -1.46 | ATP2B4 |  |
| ILMN_1709257 | 0.002764 | -1.43 | DSCR6 |  |
| ILMN_1738749 | 0.000381 | -1.26 | MAST3 |  |
| ILMN_1712431 | 8.63E-05 | -1.37 | FAM113B |  |
| ILMN_2359014 | 0.000429 | -1.44 | TBCE |  |
| ILMN_1744693 | 0.002738 | -1.33 | FGF2 |  |
| ILMN_1751789 | 0.001096 | -1.31 | HNMT |  |
| ILMN_2214098 | 0.000549 | -1.28 | BIVM |  |
| ILMN_2203891 | 0.000133 | -1.43 | SMAD7 |  |
| ILMN_2134555 | 0.000308 | -1.33 | KCTD3 |  |
| ILMN_2375003 | 0.000219 | -1.37 | MAP4K4 |  |
| ILMN_2088410 | 0.001995 | -1.24 | PSMG2 |  |
| ILMN_2065606 | 0.000995 | -1.29 | TOMM40L |  |
| ILMN_1806266 | 0.000664 | -1.30 | RAP1GDS1 |  |
| ILMN_1754795 | 0.000336 | -1.45 | FAT1 |  |
| ILMN_3187612 | 0.002884 | -1.28 | LOC100128084 |  |
| ILMN_1756999 | 0.000909 | -1.34 | RBL2 |  |
| ILMN_3260345 | 0.001706 | -1.48 | AGFG1 |  |
| ILMN_1713290 | 0.000499 | -1.25 | GLT8D1 |  |
| ILMN_2339705 | 0.002316 | -1.28 | MED8 |  |
| ILMN_1807767 | 6.42E-05 | -1.58 | KIAA0182 |  |
| ILMN_1798952 | 0.002091 | -1.28 | KDELR3 |  |
| ILMN_2377240 | 0.000232 | -1.26 | AKTIP |  |
| ILMN_1673820 | 0.00021 | -1.37 | HLTF |  |
| ILMN_2407669 | 0.003154 | -1.35 | PEAR1 |  |
| ILMN_1810327 | 0.002029 | -1.32 | HNRPUL2 |  |
| ILMN_1703754 | 0.002697 | -1.34 | CEP192 |  |
| ILMN_3176828 | 0.000595 | -1.52 | LOC100129837 |  |
| ILMN_2343278 | 0.000308 | -1.10 | PPAP2A |  |
| ILMN_2297069 | 0.000391 | -1.19 | FAM115A |  |
| ILMN_1739274 | 0.000114 | -1.28 | PDHB |  |
| ILMN_1661172 | 0.002755 | -1.48 | LOC650034 |  |
| ILMN_1736568 | 0.000442 | -1.44 | CASP2 |  |
| ILMN_2408430 | 0.00032 | -1.38 | LARGE |  |
| ILMN_1730799 | 0.001176 | -1.38 | PHF12 |  |
| ILMN_1764596 | 0.00034 | -1.42 | MPST |  |
| ILMN_2158242 | 0.001009 | -1.23 | SHOC2 |  |
| ILMN_3236530 | 0.002926 | -1.38 | LOC100130679 |  |
| ILMN_3178406 | 2.08E-05 | -1.65 | KLHL29 |  |
| ILMN_1784785 | 0.001455 | -1.20 | COPS7B |  |
| ILMN_1788053 | 0.00011 | -1.43 | SLC25A12 |  |
| ILMN_1773935 | 0.000805 | -1.28 | TMEM165 |  |
| ILMN_2070896 | 0.00188 | -1.43 | BMPR2 |  |
| ILMN_1795325 | 0.000516 | -1.30 | ACTG2 |  |
| ILMN_1707137 | 5.32E-05 | -1.53 | C17orf97 |  |
| ILMN_1666746 | 0.00062 | -1.80 | LOC153561 |  |
| ILMN_1718770 | 0.00197 | -1.81 | FLJ36070 |  |
| ILMN_2117809 | 0.000557 | -1.24 | DUXAP3 |  |
| ILMN_1670801 | 0.000868 | -1.37 | MTR |  |
| ILMN_2142117 | 0.000456 | -1.35 | LYPLAL1 |  |
| ILMN_1756793 | 0.001495 | -1.38 | POLS |  |
| ILMN_1731412 | 0.000438 | -1.33 | UBTD2 |  |
| ILMN_1660277 | 0.001251 | -1.29 | LOC731999 |  |
| ILMN_1764321 | 0.000233 | -1.39 | ACOT4 |  |
| ILMN_2330495 | 0.001807 | -1.24 | OCIAD1 |  |
| ILMN_1697503 | 0.001605 | -1.23 | DHX29 |  |
| ILMN_1670079 | 0.000198 | -1.31 | OMA1 |  |
| ILMN_1675898 | 0.000283 | -1.49 | SH3BP5 |  |
| ILMN_2041222 | 0.002212 | -1.41 | FLJ40504 |  |
| ILMN_1691188 | 0.001878 | -1.25 | UIMC1 |  |
| ILMN_3255061 | 0.000368 | -1.32 | CYTSB |  |
| ILMN_2049672 | 0.000318 | -1.81 | TMEM16C |  |
| ILMN_1656415 | 3.45E-05 | -2.24 | CDKN2C |  |
| ILMN_1808251 | 0.001649 | -1.47 | C9orf38 |  |
| ILMN_1652735 | 0.001838 | -1.48 | RFXAP |  |
| ILMN_1784630 | 0.002808 | -1.78 | KBTBD11 |  |
| ILMN_1755114 | 0.001552 | -1.26 | EIF2AK4 |  |
| ILMN_1673518 | 0.001467 | -1.19 | BRWD1 |  |
| ILMN_2153332 | 0.000592 | -1.86 | ATXN1 |  |
| ILMN_1802292 | 0.002628 | -1.35 | WDFY2 |  |
| ILMN_3206343 | 0.001174 | -1.14 | LOC644914 |  |
| ILMN_1728163 | 2.80E-06 | -1.41 | CTDSP1 |  |
| ILMN_1731287 | 0.000308 | -1.28 | ARFGAP3 |  |
| ILMN_2079098 | 0.00096 | -1.25 | C9orf80 |  |
| ILMN_1755589 | 0.000726 | -1.37 | DIP2B |  |
| ILMN_1669377 | 2.56E-05 | -1.28 | AP4B1 |  |
| ILMN_2230566 | 0.00021 | -1.52 | RAB40B |  |
| ILMN_1802109 | 0.001885 | -1.56 | KBTBD9 |  |
| ILMN_1664608 | 0.000441 | -1.40 | INPP5A |  |
| ILMN_3248781 | 0.000958 | -1.16 | SDHAP2 |  |
| ILMN_1674160 | 0.000638 | -1.15 | BIN1 |  |
| ILMN_2329958 | 0.000295 | -1.33 | ABI1 |  |
| ILMN_2406084 | 0.000272 | -1.46 | ITGA11 |  |
| ILMN_1915076 | 1.60E-05 | -1.47 |  |  |
| ILMN_1788481 | 0.002992 | -1.71 | ADAM19 |  |
| ILMN_1768480 | 0.000551 | -1.32 | VGLL4 |  |
| ILMN_1752927 | 0.002776 | -1.27 | KIAA1600 |  |
| ILMN_1708296 | 0.001546 | -1.39 | DEAF1 |  |
| ILMN_3294106 | 0.002004 | -1.25 | LOC100190938 |  |
| ILMN_1676899 | 0.000134 | -1.36 | YEATS2 |  |
| ILMN_1704139 | 0.00107 | -1.41 | DHRSX |  |
| ILMN_3235472 | 0.000609 | -1.28 | WDYHV1 |  |
| ILMN_1727577 | 0.000922 | -1.95 | GLI2 |  |
| ILMN_1824362 | 0.000298 | -1.40 |  |  |
| ILMN_2190266 | 0.00093 | -1.55 | C1orf91 |  |
| ILMN_1791702 | 0.001175 | -1.44 | SMARCA2 |  |
| ILMN_1660986 | 0.002509 | -1.35 | PER3 |  |
| ILMN_1720513 | 0.000784 | -1.64 | SETBP1 |  |
| ILMN_2390338 | 0.001251 | -1.25 | UBE2E3 |  |
| ILMN_1676333 | 0.001038 | -1.73 | LOC645465 |  |
| ILMN_1759872 | 0.0028 | -1.15 | LOC643509 |  |
| ILMN_1749612 | 0.001867 | -1.40 | LOC729101 |  |
| ILMN_1680130 | 1.32E-05 | -1.60 | DYM |  |
| ILMN_2091084 | 0.000135 | -1.33 | C8orf37 |  |
| ILMN_3247578 | 0.000258 | -1.54 | FAT1 |  |
| ILMN_1655497 | 0.00104 | -1.16 | EIF4B |  |
| ILMN_1719986 | 0.000271 | -1.38 | PIK3IP1 |  |
| ILMN_1815734 | 0.001382 | -1.72 | FCHSD2 |  |
| ILMN_1779530 | 0.002996 | -1.27 | COG6 |  |
| ILMN_2057981 | 0.002924 | -1.26 | FAM164A |  |
| ILMN_1805916 | 0.001754 | -1.21 | NIPSNAP1 |  |
| ILMN_1753500 | 0.002249 | -1.29 | ARHGAP12 |  |
| ILMN_1709026 | 0.000595 | -1.63 | C6orf145 |  |
| ILMN_2360710 | 0.00286 | -1.19 | TPM1 |  |
| ILMN_1717877 | 0.001684 | -1.39 | IVNS1ABP |  |
| ILMN_2352023 | 0.001088 | -1.22 | RIPK5 |  |
| ILMN_2342068 | 0.001218 | -1.60 | ERC1 |  |
| ILMN_2153466 | 0.003188 | -1.72 | FAM50B |  |
| ILMN_3249281 | 0.001711 | -1.31 | HOXA11AS |  |
| ILMN_1766814 | 0.001252 | -1.28 | TK2 |  |
| ILMN_2246548 | 0.000614 | -1.33 | GSTTP2 |  |
| ILMN_1893555 | 0.000789 | -1.31 |  |  |
| ILMN_1771026 | 0.002149 | -1.24 | GARS |  |
| ILMN_1668417 | 0.001203 | -1.54 | WASPIP |  |
| ILMN_2043452 | 0.003155 | -1.33 | FANCE |  |
| ILMN_1781762 | 0.000861 | -1.56 | LOC388080 |  |
| ILMN_1812312 | 0.001349 | -1.14 | NDUFS4 |  |
| ILMN_1723632 | 0.002946 | -1.26 | PIGC |  |
| ILMN_1673113 | 0.003038 | -1.48 | F2RL1 |  |
| ILMN_1796772 | 0.000637 | -1.72 | ARHGAP28 |  |
| ILMN_1766925 | 0.001632 | -1.27 | CDH13 |  |
| ILMN_1744937 | 0.001086 | -1.19 | PTPRM |  |
| ILMN_2207865 | 0.001169 | -1.53 | HIST1H3I |  |
| ILMN_1869243 | 0.000145 | -1.51 |  |  |
| ILMN_1732612 | 0.000535 | -1.41 | SHB |  |
| ILMN_1783583 | 0.001351 | -1.17 | TMEM17 |  |
| ILMN_1723815 | 0.003242 | -1.50 | NPEPPS |  |
| ILMN_1703487 | 0.000894 | -1.28 | LMO4 |  |
| ILMN_1763326 | 0.001391 | -1.33 | C5orf25 |  |
| ILMN_2340919 | 0.001081 | -1.38 | GRB10 |  |
| ILMN_1711359 | 0.001807 | -1.51 | NRN1L |  |
| ILMN_1738132 | 5.56E-05 | -2.02 | HOXA11 |  |
| ILMN_1783709 | 0.000674 | -1.13 | RRAGA |  |
| ILMN_3237656 | 7.17E-05 | -1.45 | LOC730313 |  |
| ILMN_1710523 | 0.001723 | -1.49 | ATP8B1 |  |
| ILMN_3202483 | 0.00269 | -1.17 | LOC100133876 |  |
| ILMN_1795063 | 0.000168 | -1.56 | ZADH2 |  |
| ILMN_1784113 | 0.002194 | -1.32 | NAT14 |  |
| ILMN_1762115 | 0.000854 | -1.20 | CRYZL1 |  |
| ILMN_1678605 | 0.002886 | -1.16 | CDC123 |  |
| ILMN_2199947 | 0.003191 | -1.36 | REV3L |  |
| ILMN_1680579 | 9.37E-05 | -1.37 | ATP2B4 |  |
| ILMN_1766222 | 0.000184 | -1.35 | LARP4B |  |
| ILMN_3177271 | 0.001951 | -1.25 | LOC100129585 |  |
| ILMN_1764764 | 8.97E-06 | -1.35 | MUM1 |  |
| ILMN_1813489 | 0.001499 | -1.25 | RAF1 |  |
| ILMN_1651504 | 0.000565 | -1.42 | FAM193A |  |
| ILMN_1719792 | 0.001273 | -1.71 | PHLDB2 |  |
| ILMN_1664440 | 0.00087 | -1.11 | TP53BP1 |  |
| ILMN_1716687 | 0.000278 | -1.23 | TPM1 |  |
| ILMN_1652749 | 0.002318 | -1.20 | ERF |  |
| ILMN_3263974 | 0.002934 | -1.39 | KRT18P13 |  |
| ILMN_1729216 | 0.003133 | -1.19 | CRYAB |  |
| ILMN_1662256 | 0.00173 | -1.40 | PARP6 |  |
| ILMN_1659923 | 0.000389 | -1.44 | GNAQ |  |
| ILMN_2083469 | 0.002748 | -1.16 | IRS2 |  |
| ILMN_1704195 | 0.002026 | -1.23 | FUK |  |
| ILMN_2112638 | 0.001477 | -1.24 | SVEP1 |  |
| ILMN_2089616 | 0.00196 | -1.18 | FBXO10 |  |
| ILMN_1768197 | 0.001117 | -1.26 | ROD1 |  |
| ILMN_3249262 | 0.000623 | -1.53 | LOC100132255 |  |
| ILMN_1681703 | 0.000188 | -1.28 | FOXO3 |  |
| ILMN_1760011 | 0.001987 | -1.31 | GTF2IRD2B |  |
| ILMN_1796962 | 0.003098 | -1.24 | PPP3R1 |  |
| ILMN_1665909 | 0.000209 | -1.55 | LASP1 |  |
| ILMN_3224235 | 0.001067 | -1.94 | LOC729090 |  |
| ILMN_1839051 | 0.000642 | -1.52 |  |  |
| ILMN_2408683 | 0.000748 | -1.37 | PPAP2B |  |
| ILMN_1741392 | 0.001408 | -1.28 | SLC25A20 |  |
| ILMN_2148290 | 0.002534 | -1.16 | PDCD7 |  |
| ILMN_1664560 | 0.001039 | -1.43 | DYRK1A |  |
| ILMN_1784447 | 0.000422 | -1.58 | PLCE1 |  |
| ILMN_1807919 | 1.23E-05 | -1.72 | TNS1 |  |
| ILMN_1723407 | 0.001405 | -1.43 | LOC648271 |  |
| ILMN_1726986 | 0.00256 | -1.26 | AADAT |  |
| ILMN_2062701 | 1.18E-05 | -1.92 | GAS1 |  |
| ILMN_1789702 | 0.002088 | -1.17 | GBE1 |  |
| ILMN_1696591 | 0.001825 | -1.41 | RB1 |  |
| ILMN_1692754 | 0.000836 | -1.30 | TMEM49 |  |
| ILMN_1690695 | 0.001232 | -1.25 | PEX11A |  |
| ILMN_1748783 | 0.001518 | -1.49 | LOC643240 |  |
| ILMN_2217935 | 0.002187 | -1.36 | RFC1 |  |
| ILMN_1656574 | 0.000304 | -1.35 | PCGF6 |  |
| ILMN_1673023 | 0.00313 | -1.30 | EP400 |  |
| ILMN_1846807 | 0.002955 | -1.49 |  |  |
| ILMN_1763091 | 0.000167 | -1.49 | C14orf43 |  |
| ILMN_1722089 | 0.001398 | -1.28 | RNF217 |  |
| ILMN_1796177 | 0.000265 | -1.18 | GIPC1 |  |
| ILMN_1772486 | 0.000691 | -1.42 | ELF2 |  |
| ILMN_1711810 | 0.000842 | -1.18 | PNKD |  |
| ILMN_1744023 | 1.50E-05 | -1.68 | MGC18216 |  |
| ILMN_1720083 | 0.001788 | -1.21 | EHD4 |  |
| ILMN_3273069 | 0.00152 | -1.53 | LOC100129773 |  |
| ILMN_1736730 | 0.000382 | -1.60 | LRRC16 |  |
| ILMN_1770800 | 0.002022 | -1.22 | PODN |  |
| ILMN_1671703 | 9.24E-05 | -1.29 | ACTA2 |  |
| ILMN_1782761 | 0.003166 | -1.63 | ARHGAP20 |  |
| ILMN_1747223 | 0.000969 | -1.34 | FRYL |  |
| ILMN_1851547 | 0.000529 | -1.33 |  |  |
| ILMN_2157544 | 0.000722 | -1.29 | GBF1 |  |
| ILMN_1710207 | 0.000525 | -1.35 | C10orf6 |  |
| ILMN_1763694 | 0.002385 | -1.12 | RSPRY1 |  |
| ILMN_2399310 | 0.00161 | -1.44 | MLLT10 |  |
| ILMN_1709348 | 5.41E-06 | -1.33 | ALDH1A1 |  |
| ILMN_1900520 | 0.000656 | -1.48 |  |  |
| ILMN_1774127 | 0.000189 | -1.51 | STAC |  |
| ILMN_1795275 | 4.15E-05 | -2.12 | USP53 |  |
| ILMN_1688452 | 0.002546 | -1.27 | LCMT1 |  |
| ILMN_1701244 | 0.002186 | -1.22 | ITFG2 |  |
| ILMN_1657395 | 0.002496 | -1.09 | HMGCR |  |
| ILMN_1730622 | 0.00172 | -1.15 | EVL |  |
| ILMN_1726245 | 0.000508 | -1.25 | TGFBR2 |  |
| ILMN_3236377 | 0.000326 | -1.31 | C2orf69 |  |
| ILMN_1701512 | 0.000212 | -1.54 | KIAA0391 |  |
| ILMN_1660544 | 0.000428 | -1.44 | ARRDC4 |  |
| ILMN_1758548 | 0.002648 | -1.81 | NEK7 |  |
| ILMN_2225548 | 0.003054 | -1.35 | ZNF521 |  |
| ILMN_3241441 | 0.001254 | -1.32 | MEGF6 |  |
| ILMN_1654421 | 0.000638 | -1.53 | MPHOSPH9 |  |
| ILMN_2348268 | 0.000167 | -1.26 | IFFO1 |  |
| ILMN_1891067 | 0.000236 | -1.73 |  |  |
| ILMN_2061452 | 0.002022 | -1.32 | ORC2L |  |
| ILMN_1716275 | 0.003128 | -1.52 | LOC440361 |  |
| ILMN_1795228 | 0.003209 | -1.15 | ZFAND5 |  |
| ILMN_1672022 | 0.000418 | -1.28 | EPHA4 |  |
| ILMN_3265895 | 0.003155 | -1.50 | HNRNPR |  |
| ILMN_1795383 | 0.001497 | -1.21 | RPUSD3 |  |
| ILMN_1738229 | 0.000261 | -1.28 | NDRG3 |  |
| ILMN_2374692 | 0.000196 | -1.27 | WAC |  |
| ILMN_1761684 | 0.002396 | -1.86 | WNK2 |  |
| ILMN_2116127 | 0.000584 | -1.29 | NPEPPS |  |
| ILMN_1752935 | 0.001271 | -1.37 | TMEM30B |  |
| ILMN_2336609 | 0.000193 | -1.25 | SYTL2 |  |
| ILMN_1680948 | 5.51E-05 | -1.89 | LMOD1 |  |
| ILMN_3237452 | 0.002578 | -1.15 | C17orf100 |  |
| ILMN_2329914 | 0.000111 | -1.46 | SPRY1 |  |
| ILMN_2131861 | 0.001766 | -1.23 | SOCS2 |  |
| ILMN_1719199 | 0.002815 | -1.31 | TULP3 |  |
| ILMN_1711227 | 0.002559 | -1.22 | GMDS |  |
| ILMN_1704290 | 0.002814 | -1.38 | SPTLC2 |  |
| ILMN_1868805 | 0.002691 | -1.60 |  |  |
| ILMN_1719975 | 5.06E-05 | -1.72 | HOXC4 |  |
| ILMN_2131493 | 0.003051 | -1.34 | VISA |  |
| ILMN_1673566 | 3.70E-06 | -2.13 | ADAMTS1 |  |
| ILMN_1713751 | 2.83E-05 | -1.31 | ADAM19 |  |
| ILMN_2367753 | 0.000249 | -1.36 | ATP2B4 |  |
| ILMN_2132809 | 0.001736 | -1.18 | ARHGEF10 |  |
| ILMN_3306168 | 0.002869 | -1.22 | MOBKL3 |  |
| ILMN_1713918 | 9.61E-05 | -1.56 | CYTH3 |  |
| ILMN_1701308 | 0.000786 | -1.24 | COL1A1 |  |
| ILMN_3239694 | 0.000247 | -1.37 | LOC100134412 |  |
| ILMN_1798533 | 0.002281 | -1.28 | ZNF22 |  |
| ILMN_1705035 | 0.00015 | -1.90 | FBXL7 |  |
| ILMN_2235785 | 1.12E-05 | -1.34 | KCNH6 |  |
| ILMN_1803476 | 2.14E-05 | -1.54 | KCTD20 |  |
| ILMN_2386008 | 0.000288 | -1.15 | MPZL1 |  |
| ILMN_1713682 | 0.000266 | -1.51 | FBXO11 |  |
| ILMN_1711270 | 0.000221 | -1.23 | SFRS14 |  |
| ILMN_1847870 | 0.00079 | -1.45 |  |  |
| ILMN_2372398 | 0.002494 | -1.29 | ALDH5A1 |  |
| ILMN_1752478 | 0.001892 | -1.40 | DHRS3 |  |
| ILMN_1743753 | 0.000951 | -1.46 | IFT81 |  |
| ILMN_1702447 | 0.002922 | -1.46 | IGF2BP2 |  |
| ILMN_1855746 | 0.001198 | -1.45 |  |  |
| ILMN_1740010 | 0.002259 | -1.11 | PCNX |  |
| ILMN_1664175 | 0.00312 | -1.13 | VAMP4 |  |
| ILMN_1742163 | 0.000609 | -1.58 | LOC441087 |  |
| ILMN_2141455 | 0.000895 | -1.31 | ZNF781 |  |
| ILMN_1737298 | 0.00091 | -1.20 | MAT2A |  |
| ILMN_2337923 | 0.002143 | -1.49 | TPD52L1 |  |
| ILMN_1780444 | 0.001755 | -1.33 | ARL3 |  |
| ILMN_1667692 | 4.43E-05 | -1.82 | PTGIS |  |
| ILMN_2123665 | 0.000192 | -1.56 | SBF2 |  |
| ILMN_1752086 | 0.000479 | -1.27 | C4orf41 |  |
| ILMN_1680339 | 0.000699 | -1.29 | PDGFRL |  |
| ILMN_1663836 | 0.002403 | -1.30 | LOC648374 |  |
| ILMN_1659553 | 0.000856 | -1.39 | ANAPC1 |  |
| ILMN_2079786 | 5.16E-05 | -1.61 | NUAK1 |  |
| ILMN_1651767 | 0.002181 | -1.75 | MKL1 |  |
| ILMN_1687724 | 0.000289 | -1.30 | RAP1GDS1 |  |
| ILMN_1758214 | 0.000123 | -1.27 | RARS2 |  |
| ILMN_1676986 | 0.000455 | -1.37 | NPIP |  |
| ILMN_1802699 | 0.001272 | -1.25 | PCK2 |  |
| ILMN_3233135 | 0.002135 | -1.30 | FAM178A |  |
| ILMN_2219618 | 0.002178 | -1.24 | LOC90586 |  |
| ILMN_1673960 | 0.002237 | -1.66 | MAT2B |  |
| ILMN_1809490 | 1.96E-05 | -2.19 | NCKAP5 |  |
| ILMN_2289093 | 0.003182 | -1.33 | KIAA1618 |  |
| ILMN_2180352 | 0.002887 | -1.27 | DIP2B |  |
| ILMN_1752639 | 0.000303 | -1.28 | SLC25A24 |  |
| ILMN_1684402 | 0.000925 | -1.40 | STXBP5 |  |
| ILMN_1679279 | 0.001833 | -1.55 | SYPL2 |  |
| ILMN_1769720 | 0.002125 | -1.35 | STAU2 |  |
| ILMN_1801020 | 0.002293 | -1.41 | ADK |  |
| ILMN_2412521 | 0.001572 | -1.10 | KIAA0101 |  |
| ILMN_1800096 | 0.000261 | -1.47 | MPST |  |
| ILMN_1915188 | 0.000206 | -1.39 |  |  |
| ILMN_3307877 | 2.27E-05 | -1.23 | C21orf58 |  |
| ILMN_1659845 | 7.74E-05 | -1.45 | KIAA0355 |  |
| ILMN_3180420 | 0.001401 | -1.28 | LOC100129269 |  |
| ILMN_1759154 | 0.001789 | -1.22 | PABPN1 |  |
| ILMN_1747460 | 0.002297 | -1.20 | TMEM184B |  |
| ILMN_3226211 | 0.003058 | -1.41 | MUC3A |  |
| ILMN_1763129 | 0.00083 | -1.22 | DCTPP1 |  |
| ILMN_1727495 | 0.000441 | -1.56 | L3MBTL3 |  |
| ILMN_2395652 | 0.000777 | -1.39 | PTGFR |  |
| ILMN_1751276 | 0.002324 | -1.17 | BDNF |  |
| ILMN_1736555 | 0.001551 | -1.36 | ZNF280D |  |
| ILMN_1803423 | 0.002633 | -1.32 | ARHGEF6 |  |
| ILMN_3310491 | 0.002145 | -1.10 | MIR1978 |  |
| ILMN_1653133 | 0.000799 | -1.42 | SH3D19 |  |
| ILMN_3255792 | 0.000312 | -1.28 | LOC100128505 |  |
| ILMN_1884723 | 0.000348 | -1.47 |  |  |
| ILMN_2152581 | 5.47E-05 | -1.39 | STK38 |  |
| ILMN_1685097 | 0.001922 | -1.27 | ASCC1 |  |
| ILMN_1684271 | 0.002626 | -1.36 | ACBD6 |  |
| ILMN_1765770 | 0.002913 | -1.29 | SYCP2 |  |
| ILMN_1666545 | 0.000751 | -1.59 | GCNT1 |  |
| ILMN_1678546 | 0.002287 | -1.17 | PEX11B |  |
| ILMN_1651958 | 0.000283 | -1.53 | MGP |  |
| ILMN_1740900 | 0.0019 | -1.35 | BMP4 |  |
