# Supplementary material for: HIC1 (hypermethylated in cancer 1) SUMOylation is dispensable for DNA repair but is essential for the apoptotic DNA damage response (DDR) to irreparable DNA double-strand breaks (DSBs)
Source: Oncotarget. 2016 Dec 7;8(2):2916–35. doi: 10.18632/oncotarget.13807 (PMC5356852; doi:10.18632/oncotarget.13807)
Supplement: Supplementary file 3 [file oncotarget-08-2916-s003.docx]

| **Supplemental Table 2: Normalization strategy #2 comparing Etop vs si-cntrl decreased and si-HIC-Etop vs si-cntrl decreased genes - 475 genes** | | | |
| --- | --- | --- | --- |
|  | | | |
| PROBE_ID | p-value  (Etop vs. ctr-null) | Fold-Change  (Etop vs. ctr-null) | SYMBOL |
| ILMN_1745256 | 1.79E-06 | -1.89219 | CXXC5 |
| ILMN_1829845 | 0.00132376 | -1.27948 |  |
| ILMN_1659285 | 0.000245166 | -1.19875 | PSMG1 |
| ILMN_1672080 | 0.000469422 | -1.3099 | NR2F6 |
| ILMN_1721344 | 0.000422093 | -1.20812 | MOBKL2A |
| ILMN_1741477 | 0.00111261 | -1.20017 | SMAD4 |
| ILMN_1772522 | 0.00317019 | -1.2596 | ZFP161 |
| ILMN_1783815 | 0.000764086 | -1.32787 | COG7 |
| ILMN_1683158 | 0.00159433 | -1.60534 | LOC441440 |
| ILMN_1678075 | 0.000820187 | -1.44532 | CDYL |
| ILMN_2372200 | 0.000123781 | -1.37431 | ZNF586 |
| ILMN_1790062 | 0.00225224 | -1.23083 | FAM105B |
| ILMN_1676728 | 0.00060909 | -1.29596 | DLK2 |
| ILMN_1738491 | 0.00200747 | -1.3838 | SNX30 |
| ILMN_2078124 | 0.00148231 | -1.49748 | FMO6P |
| ILMN_1743806 | 8.37E-05 | -1.31916 | MIF4GD |
| ILMN_1763634 | 0.000909741 | -1.23134 | PEX14 |
| ILMN_2066124 | 0.00160748 | -1.16579 | AFG3L2 |
| ILMN_1779228 | 1.65E-05 | -1.34412 | CDH2 |
| ILMN_1794260 | 0.00137846 | -1.23046 | FBXL10 |
| ILMN_1698020 | 0.00268291 | -1.24652 | DLC1 |
| ILMN_1695020 | 0.000278552 | -1.40115 | NEK3 |
| ILMN_1754234 | 0.00089347 | -1.17905 | ZMYND11 |
| ILMN_1655748 | 0.00285181 | -1.6788 | ZNF323 |
| ILMN_1700379 | 0.00140461 | -1.59507 | SLC26A1 |
| ILMN_3242586 | 0.000148815 | -1.36636 | RHOU |
| ILMN_1806533 | 0.00152437 | -1.59843 | PDE7B |
| ILMN_1758827 | 6.76E-05 | -1.57225 | RTN4IP1 |
| ILMN_3224204 | 0.00104743 | -1.34421 | PSMG4 |
| ILMN_1697548 | 3.04E-06 | -1.70305 | LPHN2 |
| ILMN_1760201 | 0.000396286 | -1.23222 | DNMT1 |
| ILMN_1800267 | 0.00270425 | -1.45869 | FAM13A |
| ILMN_1727574 | 0.000423263 | -1.28655 | ZNF827 |
| ILMN_1758658 | 0.00207884 | -1.12709 | FADD |
| ILMN_1771964 | 0.00149459 | -1.24066 | GSTA4 |
| ILMN_1661002 | 0.000158997 | -1.35885 | RFWD2 |
| ILMN_1654690 | 0.00307015 | -1.26859 | CECR5 |
| ILMN_3231638 | 1.80E-05 | -1.23981 | FAM160B1 |
| ILMN_1666727 | 4.93E-06 | -1.38164 | ZNF586 |
| ILMN_2177090 | 0.00121294 | -1.19512 | LOC200030 |
| ILMN_1760245 | 8.99E-05 | -1.27115 | TMEM42 |
| ILMN_1847494 | 0.00103565 | -1.26475 |  |
| ILMN_2053415 | 0.000850366 | -1.3925 | LDLR |
| ILMN_2073184 | 0.00170427 | -1.36893 | S1PR5 |
| ILMN_1660199 | 0.00209561 | -1.20149 | ACAA2 |
| ILMN_1751338 | 0.000460835 | -1.28585 | NUP133 |
| ILMN_1708936 | 0.00142911 | -1.19895 | EXOSC3 |
| ILMN_1692056 | 2.34E-05 | -1.43182 | HS3ST3A1 |
| ILMN_2313821 | 0.000193701 | -1.26142 | AIFM1 |
| ILMN_2180519 | 0.00232055 | -1.26842 | LOC729603 |
| ILMN_1719039 | 0.00279146 | -1.30876 | UBE2G1 |
| ILMN_2315964 | 0.00175337 | -1.6354 | PSRC1 |
| ILMN_1770758 | 0.00166702 | -1.69824 | AKAP6 |
| ILMN_1761069 | 0.00156473 | -1.2747 | UVRAG |
| ILMN_3273854 | 0.00199909 | -1.2399 | HNRNPA2B1 |
| ILMN_1683059 | 0.000715278 | -1.30253 | SIRT5 |
| ILMN_3238709 | 0.00237983 | -1.60654 | LOC100134068 |
| ILMN_1741156 | 0.00193375 | -1.37572 | ARMCX5 |
| ILMN_2048811 | 5.86E-06 | -1.20758 | NUBPL |
| ILMN_1810423 | 0.00323433 | -1.25659 | RPP40 |
| ILMN_1671911 | 0.00203258 | -1.14836 | MTA1 |
| ILMN_1808404 | 0.00115139 | -1.15839 | RHBDF1 |
| ILMN_1817234 | 0.00268383 | -1.53098 |  |
| ILMN_2389347 | 0.000786335 | -1.43448 | NR3C1 |
| ILMN_1713178 | 0.00072681 | -1.28855 | FAM116A |
| ILMN_1700042 | 0.000761973 | -1.42003 | TLN2 |
| ILMN_1811551 | 0.00148162 | -1.20959 | DERA |
| ILMN_1652929 | 0.000798944 | -1.28175 | POGZ |
| ILMN_1780598 | 0.00191741 | -1.26159 | PIAS1 |
| ILMN_2064898 | 0.000645398 | -1.16221 | CCDC56 |
| ILMN_1669703 | 0.000364536 | -1.25804 | TNK2 |
| ILMN_1757995 | 5.27E-05 | -1.38342 | PARP2 |
| ILMN_1719570 | 0.001707 | -1.66555 | BICC1 |
| ILMN_1775677 | 0.00291566 | -1.31343 | TYSND1 |
| ILMN_1756408 | 0.000434627 | -1.19468 | PARVA |
| ILMN_1843198 | 3.64E-07 | -1.96591 |  |
| ILMN_1781174 | 0.00238651 | -1.38217 | KIAA1009 |
| ILMN_3256325 | 0.00325548 | -1.14417 | CYB561D1 |
| ILMN_1822775 | 0.00315754 | -1.34832 |  |
| ILMN_1690282 | 0.00162278 | -1.16368 | CRADD |
| ILMN_1764549 | 0.00284866 | -1.25932 | UBE3A |
| ILMN_2392352 | 2.57E-07 | -1.81305 | CTPS2 |
| ILMN_3298167 | 0.00207353 | -1.25231 | ZSWIM7 |
| ILMN_1682264 | 0.000554245 | -1.29493 | DCAF7 |
| ILMN_1757237 | 3.63E-06 | -2.0127 | DDEF2 |
| ILMN_1724544 | 8.89E-05 | -1.31918 | PPP4R1 |
| ILMN_2227968 | 0.00107409 | -1.39416 | NTHL1 |
| ILMN_1729596 | 0.000150928 | -1.41277 | INF2 |
| ILMN_1767475 | 0.000126465 | -1.19238 | CERK |
| ILMN_1768117 | 0.00274294 | -1.3247 | RBM25 |
| ILMN_1880446 | 0.000856962 | -1.45552 |  |
| ILMN_1745946 | 0.000576516 | -1.39061 | CCDC5 |
| ILMN_1764177 | 7.33E-05 | -1.56491 | JARID2 |
| ILMN_1667932 | 0.00104257 | -1.45907 | LOC652726 |
| ILMN_1779374 | 5.79E-06 | -1.44157 | AMMECR1 |
| ILMN_1742827 | 0.00123062 | -1.29257 | EXOC4 |
| ILMN_1798581 | 0.00127328 | -1.16578 | MCM8 |
| ILMN_1782403 | 0.00195707 | -1.33161 | PRR11 |
| ILMN_2302716 | 0.00170276 | -1.17498 | ALDH18A1 |
| ILMN_3282285 | 0.00217813 | -1.35228 | LOC151457 |
| ILMN_1744647 | 0.00245705 | -1.28492 | CAND1 |
| ILMN_2055310 | 0.00104657 | -1.20947 | MBD4 |
| ILMN_1722533 | 0.000231806 | -1.37768 | KATNAL1 |
| ILMN_2136455 | 0.00188721 | -1.23763 | C3orf64 |
| ILMN_2340259 | 0.00217964 | -1.28572 | PDE4B |
| ILMN_1693210 | 5.70E-06 | -1.33624 | NSMCE2 |
| ILMN_1742798 | 0.000839589 | -1.3562 | SFRS10 |
| ILMN_3282829 | 0.00315005 | -1.35881 | LOC727913 |
| ILMN_1708025 | 0.00274488 | -1.72496 | RBPMS |
| ILMN_1752669 | 0.00099446 | -1.83339 | ALPI |
| ILMN_1714700 | 9.53E-05 | -1.64179 | TRIB2 |
| ILMN_1730572 | 0.000108462 | -1.37221 | HNRPDL |
| ILMN_1753639 | 0.00320471 | -1.26397 | MTAP |
| ILMN_3255389 | 0.00305239 | -1.26718 | LOC100049716 |
| ILMN_2318811 | 0.00259524 | -1.14399 | RANBP3 |
| ILMN_1658639 | 0.000111184 | -1.41994 | SLC46A3 |
| ILMN_3240222 | 0.000105639 | -1.27193 | PRAGMIN |
| ILMN_2410421 | 0.00204605 | -1.23129 | NBPF1 |
| ILMN_1669696 | 0.00032566 | -1.61512 | ZNF792 |
| ILMN_2391750 | 0.00304358 | -1.47776 | SFMBT1 |
| ILMN_2326675 | 5.88E-05 | -1.42332 | NR2C1 |
| ILMN_2119297 | 1.64E-05 | -1.45217 | SAMD4A |
| ILMN_1819783 | 0.00109661 | -1.47088 |  |
| ILMN_1806667 | 0.00139512 | -1.14231 | FRAS1 |
| ILMN_1730491 | 0.00208658 | -1.523 | FMNL2 |
| ILMN_3225534 | 0.00140775 | -1.38139 | RNF216L |
| ILMN_1699703 | 0.0001723 | -1.2046 | ARCN1 |
| ILMN_3270972 | 1.93E-05 | -1.70506 | ASAP2 |
| ILMN_1731612 | 0.00223114 | -1.26507 | UCHL5 |
| ILMN_1664912 | 0.00254192 | -1.1468 | IL11RA |
| ILMN_1701855 | 0.00043856 | -1.26395 | PPP1CC |
| ILMN_1795856 | 0.000180777 | -1.47578 | LOC644935 |
| ILMN_1720476 | 0.00105762 | -1.22438 | PHF2 |
| ILMN_1726786 | 0.0004952 | -1.34338 | TNRC6B |
| ILMN_1755364 | 6.58E-05 | -1.33482 | RALA |
| ILMN_1672834 | 0.000192394 | -1.40496 | SSH2 |
| ILMN_1739582 | 0.00041201 | -1.35544 | HOXA9 |
| ILMN_1784554 | 0.00174135 | -1.31774 | LOC647389 |
| ILMN_2288070 | 0.000767679 | -1.39701 | FTO |
| ILMN_1753413 | 2.61E-05 | -1.56803 | TRIOBP |
| ILMN_3300797 | 0.00153283 | -1.32887 | LOC729090 |
| ILMN_1752281 | 0.000624605 | -1.20328 | DNAJC13 |
| ILMN_2168449 | 0.00140958 | -1.28206 | DHX15 |
| ILMN_1760338 | 0.00124812 | -1.09558 | LOC643357 |
| ILMN_1684045 | 0.00105464 | -1.39149 | CDCA4 |
| ILMN_1657873 | 5.64E-06 | -1.3165 | XPO4 |
| ILMN_1764770 | 0.00291117 | -1.39029 | MGC15763 |
| ILMN_1732575 | 0.00284777 | -1.19989 | SEC14L1 |
| ILMN_1667016 | 6.48E-05 | -1.48354 | FAF1 |
| ILMN_2401641 | 0.00109336 | -1.40293 | ALDH3A2 |
| ILMN_1673798 | 0.00248356 | -1.26459 | PPOX |
| ILMN_2315780 | 0.00219468 | -1.35204 | TACC2 |
| ILMN_1706990 | 5.11E-05 | -1.31566 | ZNF271 |
| ILMN_1784287 | 3.85E-05 | -1.38678 | TGFBR3 |
| ILMN_1791232 | 9.52E-05 | -1.30102 | SPRED2 |
| ILMN_2410540 | 8.46E-05 | -1.63206 | CASP2 |
| ILMN_1791328 | 4.51E-07 | -1.72435 | STK39 |
| ILMN_1679995 | 0.00312737 | -1.36868 | MPP6 |
| ILMN_1815745 | 0.000988212 | -1.24983 | SOX4 |
| ILMN_1801387 | 0.00123191 | -1.52394 | YEATS4 |
| ILMN_1670172 | 0.000467581 | -1.19937 | WDR33 |
| ILMN_1807211 | 9.24E-06 | -1.40064 | NICN1 |
| ILMN_2172969 | 0.000745173 | -1.49793 | STXBP6 |
| ILMN_1789171 | 3.07E-06 | -1.86308 | EEF2K |
| ILMN_1671661 | 0.000203267 | -1.18228 | HSD17B7 |
| ILMN_1748719 | 0.000585687 | -1.67812 | SEC16B |
| ILMN_3239343 | 6.79E-05 | -1.63847 | STAG3L3 |
| ILMN_3289685 | 0.00189281 | -1.2629 | LOC645452 |
| ILMN_1775926 | 0.00285094 | -1.26103 | SPATA6 |
| ILMN_1732923 | 0.000444307 | -1.37916 | SIPA1L2 |
| ILMN_1727671 | 0.00325262 | -1.25311 | SSH1 |
| ILMN_2047599 | 0.00151525 | -1.15141 | TMEM50B |
| ILMN_2384513 | 0.00158126 | -1.29787 | C2CD2 |
| ILMN_2394571 | 0.000449076 | -1.30566 | FBXW11 |
| ILMN_1657993 | 0.00123231 | -1.27496 | ADNP |
| ILMN_1665655 | 0.00174717 | -1.27548 | CTDSPL2 |
| ILMN_1765044 | 0.000499696 | -1.37943 | CUTC |
| ILMN_2215545 | 0.00198403 | -1.48704 | C3orf26 |
| ILMN_1803005 | 0.00273568 | -1.28662 | MMACHC |
| ILMN_1683120 | 8.60E-05 | -1.46306 | UNG |
| ILMN_2366972 | 0.000465185 | -1.47919 | NUDT6 |
| ILMN_1791375 | 0.000514419 | -1.53965 | STAG3L2 |
| ILMN_1771728 | 0.00135481 | -1.331 | PXMP4 |
| ILMN_2075927 | 0.000116199 | -1.41002 | STK40 |
| ILMN_1764704 | 0.000536016 | -1.45649 | FAM169A |
| ILMN_2101375 | 0.00040752 | -1.40277 | CCDC77 |
| ILMN_1676804 | 0.00168825 | -1.49931 | LOC653145 |
| ILMN_1803825 | 1.46E-09 | -3.59309 | CXCL12 |
| ILMN_3240698 | 0.00222055 | -1.93403 | LOC388279 |
| ILMN_1813834 | 0.00106797 | -1.2069 | PRMT6 |
| ILMN_1754220 | 0.00318106 | -1.25621 | SF3A2 |
| ILMN_2388701 | 0.000527628 | -1.6307 | ST3GAL5 |
| ILMN_1655307 | 0.00230268 | -1.28926 | FAM136A |
| ILMN_3226663 | 0.000435629 | -1.2758 | MGC26356 |
| ILMN_1694923 | 5.54E-05 | -1.42901 | PTPN9 |
| ILMN_1753249 | 0.000245223 | -1.34165 | DDX10 |
| ILMN_2335669 | 0.000840038 | -1.32558 | ZC3H14 |
| ILMN_1695972 | 0.00126848 | -1.65392 | CCDC89 |
| ILMN_1772651 | 0.000159401 | -1.26829 | CNOT2 |
| ILMN_1758915 | 0.00024003 | -1.40427 | PDCD2 |
| ILMN_3261226 | 0.000864663 | -1.38658 | C6orf186 |
| ILMN_1681590 | 0.00198553 | -1.24343 | LARP1 |
| ILMN_1779639 | 0.000974845 | -1.38952 | IRAK1BP1 |
| ILMN_1691570 | 0.0001197 | -1.33165 | METTL5 |
| ILMN_1766054 | 0.000494696 | -1.33999 | ABCA1 |
| ILMN_1688630 | 2.68E-05 | -1.31194 | RECK |
| ILMN_2398039 | 0.00168781 | -1.44832 | TCERG1 |
| ILMN_1785795 | 0.00215026 | -1.23069 | METAP1 |
| ILMN_1726520 | 0.000272593 | -1.44207 | TDP1 |
| ILMN_1742935 | 0.00179907 | -1.38022 | ZNF33B |
| ILMN_1691428 | 0.000654655 | -1.22439 | PSMD12 |
| ILMN_1738099 | 0.000761822 | -1.18465 | C2orf34 |
| ILMN_1656840 | 0.001682 | -1.40093 | VPS13D |
| ILMN_2179083 | 0.000109405 | -1.3314 | LOXL4 |
| ILMN_2150294 | 0.000890654 | -1.21317 | FKBP14 |
| ILMN_1664772 | 0.00243536 | -1.46219 | ATP2B4 |
| ILMN_1709257 | 0.00276363 | -1.42917 | DSCR6 |
| ILMN_1738749 | 0.000380639 | -1.2642 | MAST3 |
| ILMN_3242459 | 0.000679393 | -1.18293 | DCTPP1 |
| ILMN_2359014 | 0.000429031 | -1.43556 | TBCE |
| ILMN_1744693 | 0.00273796 | -1.33146 | FGF2 |
| ILMN_1740819 | 1.58E-05 | -1.27128 | STARD7 |
| ILMN_2203891 | 0.000132916 | -1.43165 | SMAD7 |
| ILMN_1742238 | 0.000807095 | -1.11713 | SET |
| ILMN_1723020 | 0.0014826 | -1.45076 | MAP3K1 |
| ILMN_1730940 | 7.18E-05 | -1.28282 | KLHDC3 |
| ILMN_2088410 | 0.00199447 | -1.24054 | PSMG2 |
| ILMN_2065606 | 0.000994824 | -1.28984 | TOMM40L |
| ILMN_2133534 | 0.000282243 | -1.37954 | SMA4 |
| ILMN_1806266 | 0.000663431 | -1.30007 | RAP1GDS1 |
| ILMN_3187612 | 0.00288369 | -1.28085 | LOC100128084 |
| ILMN_1756999 | 0.000909318 | -1.33815 | RBL2 |
| ILMN_3260345 | 0.00170607 | -1.47623 | AGFG1 |
| ILMN_2403247 | 0.00167614 | -1.30856 | CMTM7 |
| ILMN_2339705 | 0.00231521 | -1.28157 | MED8 |
| ILMN_1798952 | 0.00209126 | -1.27736 | KDELR3 |
| ILMN_1787275 | 0.00171566 | -1.25327 | STAG3 |
| ILMN_2377240 | 0.000231627 | -1.25537 | AKTIP |
| ILMN_1673820 | 0.000209846 | -1.37417 | HLTF |
| ILMN_3176828 | 0.00059541 | -1.51927 | LOC100129837 |
| ILMN_1732809 | 0.00040277 | -1.22921 | ALG9 |
| ILMN_1713846 | 6.31E-06 | -1.92567 | PPM1H |
| ILMN_1813277 | 0.000203023 | -1.46081 | SUPT3H |
| ILMN_1736568 | 0.000442429 | -1.44437 | CASP2 |
| ILMN_2408430 | 0.000320403 | -1.38383 | LARGE |
| ILMN_1764596 | 0.00034039 | -1.42386 | MPST |
| ILMN_3236530 | 0.00292624 | -1.38094 | LOC100130679 |
| ILMN_1659027 | 0.000325053 | -1.26177 | SLC2A1 |
| ILMN_1765606 | 0.000577778 | -1.50206 | YAF2 |
| ILMN_1784785 | 0.00145462 | -1.19982 | COPS7B |
| ILMN_1788053 | 0.000110047 | -1.42819 | SLC25A12 |
| ILMN_1753164 | 0.000423811 | -1.33558 | IPO8 |
| ILMN_1773935 | 0.000805189 | -1.27816 | TMEM165 |
| ILMN_1666746 | 0.000619991 | -1.79823 | LOC153561 |
| ILMN_2117809 | 0.000556964 | -1.24139 | DUXAP3 |
| ILMN_1729319 | 0.00305028 | -1.15755 | USP7 |
| ILMN_2234343 | 5.01E-05 | -1.35468 | ACP6 |
| ILMN_1670801 | 0.000868165 | -1.37222 | MTR |
| ILMN_2142117 | 0.000456173 | -1.35349 | LYPLAL1 |
| ILMN_1756793 | 0.00149446 | -1.38294 | POLS |
| ILMN_1714384 | 1.92E-06 | -1.48253 | PCCA |
| ILMN_1791002 | 0.000526842 | -1.53295 | SKP2 |
| ILMN_1660277 | 0.00125101 | -1.28687 | LOC731999 |
| ILMN_1764321 | 0.000232968 | -1.38817 | ACOT4 |
| ILMN_2330495 | 0.00180669 | -1.23977 | OCIAD1 |
| ILMN_1697503 | 0.00160538 | -1.22907 | DHX29 |
| ILMN_1670079 | 0.000197857 | -1.30973 | OMA1 |
| ILMN_1789436 | 0.000211186 | -1.31858 | C1orf218 |
| ILMN_1675898 | 0.000282531 | -1.48637 | SH3BP5 |
| ILMN_1670796 | 5.33E-05 | -1.29577 | EXOSC10 |
| ILMN_1691188 | 0.00187802 | -1.24794 | UIMC1 |
| ILMN_2049672 | 0.000318358 | -1.80697 | TMEM16C |
| ILMN_1716006 | 0.00191453 | -1.25539 | C18orf54 |
| ILMN_1651776 | 0.00152688 | -1.25593 | FHOD1 |
| ILMN_1792305 | 0.000161114 | -1.37186 | ZNF318 |
| ILMN_1808251 | 0.00164933 | -1.4651 | C9orf38 |
| ILMN_1652735 | 0.00183816 | -1.4776 | RFXAP |
| ILMN_1784630 | 0.00280796 | -1.78201 | KBTBD11 |
| ILMN_1755114 | 0.00155179 | -1.26225 | EIF2AK4 |
| ILMN_1713759 | 0.000252924 | -1.26328 | UBE2J1 |
| ILMN_1802292 | 0.00262741 | -1.35198 | WDFY2 |
| ILMN_1712389 | 0.00198111 | -1.15642 | CKLF |
| ILMN_1759952 | 0.00173132 | -1.16988 | PSMA5 |
| ILMN_1761260 | 7.49E-05 | -1.36763 | COBLL1 |
| ILMN_1731287 | 0.000307784 | -1.27675 | ARFGAP3 |
| ILMN_2079098 | 0.000959615 | -1.25198 | C9orf80 |
| ILMN_1755589 | 0.00072616 | -1.3658 | DIP2B |
| ILMN_1676361 | 0.000735107 | -1.39056 | ARHGAP22 |
| ILMN_1689585 | 4.17E-05 | -1.3415 | C20orf194 |
| ILMN_3248781 | 0.000957991 | -1.15695 | SDHAP2 |
| ILMN_1674160 | 0.000637644 | -1.14813 | BIN1 |
| ILMN_2329958 | 0.000294783 | -1.32521 | ABI1 |
| ILMN_1813236 | 7.64E-05 | -1.44196 | C6orf136 |
| ILMN_1695357 | 2.67E-07 | -1.56461 | CCDC99 |
| ILMN_1705774 | 0.000316968 | -1.46423 | TIGD5 |
| ILMN_1788481 | 0.00299189 | -1.71373 | ADAM19 |
| ILMN_1768480 | 0.000550906 | -1.31901 | VGLL4 |
| ILMN_1675695 | 0.000741428 | -1.37231 | PDS5B |
| ILMN_1752927 | 0.00277598 | -1.27187 | KIAA1600 |
| ILMN_1708296 | 0.00154612 | -1.38549 | DEAF1 |
| ILMN_3294106 | 0.00200432 | -1.24705 | LOC100190938 |
| ILMN_1713496 | 3.77E-05 | -1.71593 | ST3GAL5 |
| ILMN_3191227 | 0.000132523 | -1.62873 | LOC100129267 |
| ILMN_2126055 | 0.00151379 | -1.26735 | ASB5 |
| ILMN_1704619 | 0.00318756 | -1.12021 | VPS29 |
| ILMN_1669982 | 2.56E-05 | -2.08397 | CCDC85A |
| ILMN_1684158 | 1.59E-07 | -1.7539 | GPT2 |
| ILMN_3235472 | 0.000609089 | -1.27988 | WDYHV1 |
| ILMN_1727577 | 0.0009224 | -1.95095 | GLI2 |
| ILMN_1824362 | 0.00029769 | -1.40064 |  |
| ILMN_2190266 | 0.000929587 | -1.54697 | C1orf91 |
| ILMN_1660986 | 0.00250873 | -1.34863 | PER3 |
| ILMN_1720513 | 0.000783916 | -1.63501 | SETBP1 |
| ILMN_2390338 | 0.00125134 | -1.24529 | UBE2E3 |
| ILMN_1676333 | 0.00103799 | -1.73395 | LOC645465 |
| ILMN_1759872 | 0.00279974 | -1.14862 | LOC643509 |
| ILMN_3194508 | 8.10E-06 | -1.78582 | ASAP2 |
| ILMN_2344002 | 0.000150479 | -1.42942 | SIP1 |
| ILMN_1685636 | 0.00130989 | -1.60969 | KCNN2 |
| ILMN_1813669 | 1.00E-05 | -1.47288 | ANKS1A |
| ILMN_2361862 | 0.000850046 | -1.38004 | VLDLR |
| ILMN_1815734 | 0.00138176 | -1.71725 | FCHSD2 |
| ILMN_1779530 | 0.00299588 | -1.26578 | COG6 |
| ILMN_1805916 | 0.00175378 | -1.21271 | NIPSNAP1 |
| ILMN_1753500 | 0.00224933 | -1.29064 | ARHGAP12 |
| ILMN_1694311 | 0.000951483 | -1.29926 | NUDT6 |
| ILMN_1717477 | 0.00172942 | -1.29418 | PSD3 |
| ILMN_2342068 | 0.00121803 | -1.59628 | ERC1 |
| ILMN_2153466 | 0.00318814 | -1.7187 | FAM50B |
| ILMN_3249281 | 0.00171126 | -1.30906 | HOXA11AS |
| ILMN_2111187 | 0.000308143 | -1.28517 | ELOVL6 |
| ILMN_1766814 | 0.00125239 | -1.28143 | TK2 |
| ILMN_1793732 | 4.22E-05 | -1.38269 | FARS2 |
| ILMN_2246548 | 0.000613992 | -1.33461 | GSTTP2 |
| ILMN_1742147 | 0.000456762 | -1.13728 | UBL4A |
| ILMN_2043452 | 0.00315513 | -1.33205 | FANCE |
| ILMN_1781762 | 0.00086091 | -1.55926 | LOC388080 |
| ILMN_1812312 | 0.00135106 | -1.14343 | NDUFS4 |
| ILMN_1723632 | 0.00294639 | -1.26001 | PIGC |
| ILMN_3240765 | 0.000603804 | -1.46205 | ANO3 |
| ILMN_1766925 | 0.00163237 | -1.27064 | CDH13 |
| ILMN_1660871 | 9.29E-06 | -1.37153 | NEK6 |
| ILMN_2117904 | 0.000571316 | -1.24258 | ZNF22 |
| ILMN_1658678 | 0.000253341 | -1.26148 | SAAL1 |
| ILMN_2207865 | 0.00116936 | -1.53088 | HIST1H3I |
| ILMN_1869243 | 0.000144534 | -1.50732 |  |
| ILMN_1732612 | 0.000535156 | -1.41404 | SHB |
| ILMN_1759252 | 9.83E-05 | -1.19549 | ADD1 |
| ILMN_1783583 | 0.00135133 | -1.17314 | TMEM17 |
| ILMN_1723815 | 0.00324222 | -1.49684 | NPEPPS |
| ILMN_1763326 | 0.001391 | -1.33448 | C5orf25 |
| ILMN_2340919 | 0.00108146 | -1.38263 | GRB10 |
| ILMN_1711359 | 0.00180713 | -1.50612 | NRN1L |
| ILMN_1738132 | 5.56E-05 | -2.02045 | HOXA11 |
| ILMN_1710523 | 0.00172281 | -1.4905 | ATP8B1 |
| ILMN_3202483 | 0.00268998 | -1.17156 | LOC100133876 |
| ILMN_1795063 | 0.000167946 | -1.56366 | ZADH2 |
| ILMN_1784113 | 0.00219349 | -1.32008 | NAT14 |
| ILMN_1762115 | 0.000854067 | -1.19895 | CRYZL1 |
| ILMN_1678605 | 0.00288598 | -1.15592 | CDC123 |
| ILMN_2199947 | 0.00319152 | -1.35597 | REV3L |
| ILMN_3177271 | 0.00195099 | -1.25151 | LOC100129585 |
| ILMN_1664440 | 0.000869962 | -1.11226 | TP53BP1 |
| ILMN_1716687 | 0.000277781 | -1.23308 | TPM1 |
| ILMN_1652749 | 0.0023177 | -1.1953 | ERF |
| ILMN_1742578 | 0.000263978 | -1.24175 | MKLN1 |
| ILMN_1704195 | 0.00202615 | -1.23037 | FUK |
| ILMN_2089616 | 0.00196075 | -1.17518 | FBXO10 |
| ILMN_1768197 | 0.00111714 | -1.25759 | ROD1 |
| ILMN_3249262 | 0.000623136 | -1.53446 | LOC100132255 |
| ILMN_1672878 | 0.000719748 | -1.27563 | ABR |
| ILMN_3224235 | 0.00106699 | -1.93983 | LOC729090 |
| ILMN_1741392 | 0.00140811 | -1.28297 | SLC25A20 |
| ILMN_2148290 | 0.00253757 | -1.16266 | PDCD7 |
| ILMN_1723407 | 0.00140463 | -1.42865 | LOC648271 |
| ILMN_1726986 | 0.00255949 | -1.25717 | AADAT |
| ILMN_1696591 | 0.00182508 | -1.41391 | RB1 |
| ILMN_1692754 | 0.000836294 | -1.30355 | TMEM49 |
| ILMN_1798712 | 0.000107821 | -1.24297 | USP4 |
| ILMN_1656574 | 0.000303889 | -1.35411 | PCGF6 |
| ILMN_1846807 | 0.00295522 | -1.48826 |  |
| ILMN_2086238 | 3.68E-05 | -1.25521 | SMYD4 |
| ILMN_2356672 | 0.000326791 | -1.18339 | EIF2B4 |
| ILMN_1722089 | 0.00139812 | -1.27869 | RNF217 |
| ILMN_1796177 | 0.000265418 | -1.18003 | GIPC1 |
| ILMN_1772486 | 0.000690929 | -1.41984 | ELF2 |
| ILMN_1711810 | 0.000841932 | -1.1769 | PNKD |
| ILMN_3273069 | 0.00152005 | -1.52623 | LOC100129773 |
| ILMN_1851547 | 0.000528817 | -1.33272 |  |
| ILMN_2157544 | 0.000722033 | -1.29145 | GBF1 |
| ILMN_1710207 | 0.000524886 | -1.34669 | C10orf6 |
| ILMN_1763694 | 0.00238398 | -1.12189 | RSPRY1 |
| ILMN_2399310 | 0.00160956 | -1.43822 | MLLT10 |
| ILMN_1688452 | 0.00254578 | -1.26605 | LCMT1 |
| ILMN_1657395 | 0.00249633 | -1.08991 | HMGCR |
| ILMN_1726245 | 0.000508128 | -1.24855 | TGFBR2 |
| ILMN_3236377 | 0.000325629 | -1.31098 | C2orf69 |
| ILMN_1701512 | 0.000212079 | -1.53546 | KIAA0391 |
| ILMN_1748819 | 0.000735223 | -1.18126 | MRPL22 |
| ILMN_1660544 | 0.000428336 | -1.43541 | ARRDC4 |
| ILMN_1690844 | 0.00249692 | -1.25502 | LOC387820 |
| ILMN_1758548 | 0.00264797 | -1.814 | NEK7 |
| ILMN_2101885 | 9.69E-05 | -1.27786 | TUBB |
| ILMN_3241441 | 0.00125461 | -1.32483 | MEGF6 |
| ILMN_1654421 | 0.000638022 | -1.53387 | MPHOSPH9 |
| ILMN_1690523 | 5.03E-06 | -1.65865 | LRRC20 |
| ILMN_1891067 | 0.000235788 | -1.72644 |  |
| ILMN_1782551 | 0.000192604 | -1.55765 | E2F5 |
| ILMN_1672022 | 0.000417916 | -1.27712 | EPHA4 |
| ILMN_3265895 | 0.00315517 | -1.50158 | HNRNPR |
| ILMN_3244506 | 0.000110682 | -1.3337 | LOC441089 |
| ILMN_1795383 | 0.00149734 | -1.21313 | RPUSD3 |
| ILMN_1738229 | 0.000260664 | -1.27995 | NDRG3 |
| ILMN_1761684 | 0.0023966 | -1.86213 | WNK2 |
| ILMN_2116127 | 0.000584091 | -1.28503 | NPEPPS |
| ILMN_1752935 | 0.00127076 | -1.37259 | TMEM30B |
| ILMN_3237452 | 0.00257825 | -1.15211 | C17orf100 |
| ILMN_1684746 | 0.000749963 | -1.23566 | IPO11 |
| ILMN_1652512 | 2.61E-05 | -1.33767 | C2CD2 |
| ILMN_1711227 | 0.00255948 | -1.22272 | GMDS |
| ILMN_1704290 | 0.00281405 | -1.37828 | SPTLC2 |
| ILMN_2131493 | 0.0030506 | -1.3374 | VISA |
| ILMN_1746393 | 0.00296963 | -1.42147 | TSEN2 |
| ILMN_1713751 | 2.83E-05 | -1.30795 | ADAM19 |
| ILMN_3306168 | 0.00286879 | -1.21559 | MOBKL3 |
| ILMN_1701308 | 0.000785788 | -1.24479 | COL1A1 |
| ILMN_1798533 | 0.00228105 | -1.28146 | ZNF22 |
| ILMN_1808713 | 0.000715582 | -1.6207 | HSD17B2 |
| ILMN_2235785 | 1.13E-05 | -1.33632 | KCNH6 |
| ILMN_2386008 | 0.000288137 | -1.15231 | MPZL1 |
| ILMN_1711270 | 0.000221337 | -1.23389 | SFRS14 |
| ILMN_1686968 | 2.41E-05 | -1.42013 | ZNF362 |
| ILMN_2372398 | 0.00249439 | -1.28814 | ALDH5A1 |
| ILMN_1702447 | 0.00292232 | -1.45971 | IGF2BP2 |
| ILMN_1740010 | 0.00225919 | -1.11084 | PCNX |
| ILMN_2141455 | 0.000895392 | -1.31094 | ZNF781 |
| ILMN_1737298 | 0.000910173 | -1.20308 | MAT2A |
| ILMN_1700955 | 0.000182589 | -1.26807 | TCTEX1D2 |
| ILMN_1780444 | 0.00175468 | -1.33166 | ARL3 |
| ILMN_1752086 | 0.000479198 | -1.27485 | C4orf41 |
| ILMN_1657204 | 0.000856844 | -1.18474 | SAE1 |
| ILMN_1680339 | 0.000698592 | -1.28883 | PDGFRL |
| ILMN_1756086 | 0.000205162 | -1.21701 | INTS3 |
| ILMN_1663836 | 0.00240297 | -1.29753 | LOC648374 |
| ILMN_1726306 | 0.000229764 | -1.42652 | HMBS |
| ILMN_1678087 | 0.000601082 | -1.45303 | MAP3K4 |
| ILMN_3233135 | 0.00213562 | -1.29605 | FAM178A |
| ILMN_2219618 | 0.00217745 | -1.23684 | LOC90586 |
| ILMN_1673960 | 0.00223674 | -1.65867 | MAT2B |
| ILMN_2289093 | 0.00318213 | -1.32559 | KIAA1618 |
| ILMN_2180352 | 0.00288649 | -1.27014 | DIP2B |
| ILMN_1752639 | 0.000303335 | -1.27559 | SLC25A24 |
| ILMN_1769720 | 0.00212457 | -1.35476 | STAU2 |
| ILMN_1801020 | 0.00229316 | -1.40544 | ADK |
| ILMN_2412521 | 0.00156849 | -1.09863 | KIAA0101 |
| ILMN_1800096 | 0.000260693 | -1.47044 | MPST |
| ILMN_2217809 | 0.00192673 | -1.13066 | TMEM126A |
| ILMN_1698209 | 0.000621302 | -1.27169 | AGPS |
| ILMN_3307877 | 2.27E-05 | -1.2254 | C21orf58 |
| ILMN_2408001 | 0.00176257 | -1.23562 | RFWD2 |
| ILMN_1759154 | 0.00178924 | -1.21705 | PABPN1 |
| ILMN_1747460 | 0.00229712 | -1.20181 | TMEM184B |
| ILMN_3226211 | 0.00305845 | -1.40978 | MUC3A |
| ILMN_1763129 | 0.000829851 | -1.22291 | DCTPP1 |
| ILMN_1741300 | 0.000140462 | -1.23138 | ZNF407 |
| ILMN_2395652 | 0.00077678 | -1.38969 | PTGFR |
| ILMN_1803423 | 0.00263321 | -1.32315 | ARHGEF6 |
| ILMN_1653133 | 0.000799355 | -1.42152 | SH3D19 |
| ILMN_1884723 | 0.0003475 | -1.47063 |  |
| ILMN_1685097 | 0.00192232 | -1.2714 | ASCC1 |
| ILMN_3232219 | 0.00230274 | -1.25144 | LOC100134189 |
| ILMN_1776213 | 9.21E-07 | -1.51392 | RGMB |
| ILMN_1684271 | 0.002626 | -1.35509 | ACBD6 |
| ILMN_1765770 | 0.00291288 | -1.28574 | SYCP2 |
| ILMN_1678546 | 0.00228714 | -1.16506 | PEX11B |
