# Supplementary material for: HIC1 (hypermethylated in cancer 1) SUMOylation is dispensable for DNA repair but is essential for the apoptotic DNA damage response (DDR) to irreparable DNA double-strand breaks (DSBs)
Source: Oncotarget. 2016 Dec 7;8(2):2916–35. doi: 10.18632/oncotarget.13807 (PMC5356852; doi:10.18632/oncotarget.13807)
Supplement: Supplementary file 4 [file oncotarget-08-2916-s004.docx]

| **Supplemental Table 3: Intersection of normalization strategies 1&2 - 319 genes** | | | |
| --- | --- | --- | --- |
|  | | | |
| PROBE_ID | p-value  (Etop vs. ctr-null) | Fold-Change  (Etop vs. ctr-null) | SYMBOL |
| ILMN_1726245 | 0.000508 | -1.24855 | TGFBR2 |
| ILMN_1739582 | 0.000412 | -1.35544 | HOXA9 |
| ILMN_1764321 | 0.000233 | -1.38817 | ACOT4 |
| ILMN_2049672 | 0.000318 | -1.80696 | TMEM16C |
| ILMN_2401641 | 0.001093 | -1.40293 | ALDH3A2 |
| ILMN_1658639 | 0.000111 | -1.41994 | SLC46A3 |
| ILMN_3225534 | 0.001408 | -1.38139 | RNF216L |
| ILMN_1761684 | 0.002396 | -1.86214 | WNK2 |
| ILMN_1713751 | 2.83E-05 | -1.30795 | ADAM19 |
| ILMN_1676728 | 0.000609 | -1.29596 | DLK2 |
| ILMN_1669696 | 0.000326 | -1.61512 | ZNF792 |
| ILMN_2078124 | 0.001482 | -1.49748 | FMO6P |
| ILMN_2089616 | 0.00196 | -1.17518 | FBXO10 |
| ILMN_2227968 | 0.001074 | -1.39416 | NTHL1 |
| ILMN_3233135 | 0.002135 | -1.29605 | FAM178A |
| ILMN_1752669 | 0.000994 | -1.8334 | ALPI |
| ILMN_1710207 | 0.000525 | -1.34669 | C10orf6 |
| ILMN_1711810 | 0.000842 | -1.1769 | PNKD |
| ILMN_2136455 | 0.001887 | -1.23763 | C3orf64 |
| ILMN_1655307 | 0.002303 | -1.28926 | FAM136A |
| ILMN_3224204 | 0.001047 | -1.34421 | PSMG4 |
| ILMN_1713178 | 0.000727 | -1.28855 | FAM116A |
| ILMN_1696591 | 0.001825 | -1.41392 | RB1 |
| ILMN_2384513 | 0.001581 | -1.29787 | C2CD2 |
| ILMN_1738229 | 0.000261 | -1.27995 | NDRG3 |
| ILMN_1729596 | 0.000151 | -1.41277 | INF2 |
| ILMN_1660544 | 0.000428 | -1.43541 | ARRDC4 |
| ILMN_2203891 | 0.000133 | -1.43165 | SMAD7 |
| ILMN_1772486 | 0.000691 | -1.41985 | ELF2 |
| ILMN_2313821 | 0.000194 | -1.26143 | AIFM1 |
| ILMN_2372200 | 0.000124 | -1.37431 | ZNF586 |
| ILMN_3273069 | 0.00152 | -1.52624 | LOC100129773 |
| ILMN_1742935 | 0.001799 | -1.38021 | ZNF33B |
| ILMN_1670801 | 0.000868 | -1.37221 | MTR |
| ILMN_1736568 | 0.000442 | -1.44437 | CASP2 |
| ILMN_1700379 | 0.001405 | -1.59507 | SLC26A1 |
| ILMN_3273854 | 0.001999 | -1.2399 | HNRNPA2B1 |
| ILMN_1779639 | 0.000975 | -1.38953 | IRAK1BP1 |
| ILMN_3249262 | 0.000623 | -1.53446 | LOC100132255 |
| ILMN_1880446 | 0.000857 | -1.45551 |  |
| ILMN_1673960 | 0.002237 | -1.65867 | MAT2B |
| ILMN_1656574 | 0.000304 | -1.35412 | PCGF6 |
| ILMN_1688630 | 2.68E-05 | -1.31194 | RECK |
| ILMN_3224235 | 0.001067 | -1.93983 | LOC729090 |
| ILMN_3239343 | 6.79E-05 | -1.63847 | STAG3L3 |
| ILMN_1798952 | 0.002091 | -1.27736 | KDELR3 |
| ILMN_1762115 | 0.000854 | -1.19895 | CRYZL1 |
| ILMN_1738132 | 5.56E-05 | -2.02044 | HOXA11 |
| ILMN_1771964 | 0.001494 | -1.24066 | GSTA4 |
| ILMN_1810423 | 0.003234 | -1.25659 | RPP40 |
| ILMN_2073184 | 0.001704 | -1.36893 | S1PR5 |
| ILMN_1752927 | 0.002776 | -1.27186 | KIAA1600 |
| ILMN_2326675 | 5.88E-05 | -1.42332 | NR2C1 |
| ILMN_1701308 | 0.000786 | -1.2448 | COL1A1 |
| ILMN_1803423 | 0.002633 | -1.32315 | ARHGEF6 |
| ILMN_1846807 | 0.002955 | -1.48826 |  |
| ILMN_1748719 | 0.000586 | -1.67812 | SEC16B |
| ILMN_1800096 | 0.000261 | -1.47044 | MPST |
| ILMN_2142117 | 0.000456 | -1.35349 | LYPLAL1 |
| ILMN_1755589 | 0.000726 | -1.3658 | DIP2B |
| ILMN_1851547 | 0.000529 | -1.33272 |  |
| ILMN_1702447 | 0.002922 | -1.45971 | IGF2BP2 |
| ILMN_2391750 | 0.003044 | -1.47775 | SFMBT1 |
| ILMN_1775677 | 0.002916 | -1.31343 | TYSND1 |
| ILMN_1768197 | 0.001117 | -1.25759 | ROD1 |
| ILMN_1791375 | 0.000514 | -1.53965 | STAG3L2 |
| ILMN_1822775 | 0.003158 | -1.34832 |  |
| ILMN_1796177 | 0.000265 | -1.18003 | GIPC1 |
| ILMN_3238709 | 0.00238 | -1.60654 | LOC100134068 |
| ILMN_2190266 | 0.00093 | -1.54697 | C1orf91 |
| ILMN_1765044 | 0.0005 | -1.37944 | CUTC |
| ILMN_3256325 | 0.003256 | -1.14416 | CYB561D1 |
| ILMN_1764596 | 0.00034 | -1.42386 | MPST |
| ILMN_1737298 | 0.00091 | -1.20308 | MAT2A |
| ILMN_1788053 | 0.00011 | -1.42818 | SLC25A12 |
| ILMN_1754220 | 0.003181 | -1.25622 | SF3A2 |
| ILMN_2141455 | 0.000895 | -1.31094 | ZNF781 |
| ILMN_1806667 | 0.001395 | -1.14231 | FRAS1 |
| ILMN_1784287 | 3.85E-05 | -1.38678 | TGFBR3 |
| ILMN_1654690 | 0.00307 | -1.26859 | CECR5 |
| ILMN_1720513 | 0.000784 | -1.635 | SETBP1 |
| ILMN_1775926 | 0.002851 | -1.26103 | SPATA6 |
| ILMN_3176828 | 0.000595 | -1.51927 | LOC100129837 |
| ILMN_1708296 | 0.001546 | -1.38549 | DEAF1 |
| ILMN_1758548 | 0.002648 | -1.814 | NEK7 |
| ILMN_1744647 | 0.002457 | -1.28492 | CAND1 |
| ILMN_1752086 | 0.000479 | -1.27484 | C4orf41 |
| ILMN_2199947 | 0.003191 | -1.35597 | REV3L |
| ILMN_1678605 | 0.002886 | -1.15592 | CDC123 |
| ILMN_2066124 | 0.001608 | -1.16579 | AFG3L2 |
| ILMN_1683158 | 0.001594 | -1.60534 | LOC441440 |
| ILMN_1795063 | 0.000168 | -1.56366 | ZADH2 |
| ILMN_1672022 | 0.000418 | -1.27712 | EPHA4 |
| ILMN_1678546 | 0.002287 | -1.16506 | PEX11B |
| ILMN_1764177 | 7.33E-05 | -1.56491 | JARID2 |
| ILMN_1758915 | 0.00024 | -1.40427 | PDCD2 |
| ILMN_1732612 | 0.000535 | -1.41404 | SHB |
| ILMN_1676333 | 0.001038 | -1.73395 | LOC645465 |
| ILMN_1741156 | 0.001934 | -1.37572 | ARMCX5 |
| ILMN_1673820 | 0.00021 | -1.37417 | HLTF |
| ILMN_1745946 | 0.000577 | -1.3906 | CCDC5 |
| ILMN_3242586 | 0.000149 | -1.36636 | RHOU |
| ILMN_2340259 | 0.00218 | -1.28572 | PDE4B |
| ILMN_1781762 | 0.000861 | -1.55926 | LOC388080 |
| ILMN_1699703 | 0.000172 | -1.2046 | ARCN1 |
| ILMN_1711359 | 0.001807 | -1.50612 | NRN1L |
| ILMN_1798533 | 0.002281 | -1.28146 | ZNF22 |
| ILMN_2180352 | 0.002887 | -1.27014 | DIP2B |
| ILMN_3236377 | 0.000326 | -1.31099 | C2orf69 |
| ILMN_2339705 | 0.002316 | -1.28157 | MED8 |
| ILMN_1723632 | 0.002946 | -1.26 | PIGC |
| ILMN_1764704 | 0.000536 | -1.4565 | FAM169A |
| ILMN_1678075 | 0.00082 | -1.44533 | CDYL |
| ILMN_1795383 | 0.001497 | -1.21313 | RPUSD3 |
| ILMN_2288070 | 0.000768 | -1.39701 | FTO |
| ILMN_1666746 | 0.00062 | -1.79823 | LOC153561 |
| ILMN_1684045 | 0.001055 | -1.39149 | CDCA4 |
| ILMN_1802292 | 0.002628 | -1.35198 | WDFY2 |
| ILMN_1805916 | 0.001754 | -1.21271 | NIPSNAP1 |
| ILMN_1664912 | 0.002542 | -1.1468 | IL11RA |
| ILMN_1664772 | 0.002435 | -1.46219 | ATP2B4 |
| ILMN_1891067 | 0.000236 | -1.72644 |  |
| ILMN_1784785 | 0.001455 | -1.19983 | COPS7B |
| ILMN_1753639 | 0.003204 | -1.26398 | MTAP |
| ILMN_1813834 | 0.001068 | -1.2069 | PRMT6 |
| ILMN_1684271 | 0.002626 | -1.35509 | ACBD6 |
| ILMN_1817234 | 0.002684 | -1.53098 |  |
| ILMN_3306168 | 0.002869 | -1.21559 | MOBKL3 |
| ILMN_2153466 | 0.003188 | -1.7187 | FAM50B |
| ILMN_1784630 | 0.002808 | -1.78202 | KBTBD11 |
| ILMN_2342068 | 0.001218 | -1.59628 | ERC1 |
| ILMN_1869243 | 0.000145 | -1.50732 |  |
| ILMN_1719570 | 0.001707 | -1.66555 | BICC1 |
| ILMN_1764770 | 0.002911 | -1.39029 | MGC15763 |
| ILMN_1788481 | 0.002992 | -1.71373 | ADAM19 |
| ILMN_1819783 | 0.001097 | -1.47089 |  |
| ILMN_1758658 | 0.002079 | -1.12709 | FADD |
| ILMN_1701512 | 0.000212 | -1.53545 | KIAA0391 |
| ILMN_1800267 | 0.002704 | -1.45869 | FAM13A |
| ILMN_3249281 | 0.001711 | -1.30906 | HOXA11AS |
| ILMN_1763129 | 0.00083 | -1.22291 | DCTPP1 |
| ILMN_1690282 | 0.001623 | -1.16368 | CRADD |
| ILMN_1652735 | 0.001838 | -1.4776 | RFXAP |
| ILMN_2207865 | 0.001169 | -1.53088 | HIST1H3I |
| ILMN_1655748 | 0.002852 | -1.6788 | ZNF323 |
| ILMN_1720476 | 0.001057 | -1.22438 | PHF2 |
| ILMN_1671661 | 0.000203 | -1.18228 | HSD17B7 |
| ILMN_1727577 | 0.000922 | -1.95095 | GLI2 |
| ILMN_1773935 | 0.000805 | -1.27817 | TMEM165 |
| ILMN_1695972 | 0.001268 | -1.65392 | CCDC89 |
| ILMN_2047599 | 0.001515 | -1.15141 | TMEM50B |
| ILMN_1670079 | 0.000198 | -1.30973 | OMA1 |
| ILMN_1681590 | 0.001986 | -1.24343 | LARP1 |
| ILMN_1884723 | 0.000348 | -1.47062 |  |
| ILMN_2390338 | 0.001251 | -1.24528 | UBE2E3 |
| ILMN_1695020 | 0.000279 | -1.40115 | NEK3 |
| ILMN_2318811 | 0.002595 | -1.14399 | RANBP3 |
| ILMN_1726986 | 0.00256 | -1.25717 | AADAT |
| ILMN_2157544 | 0.000722 | -1.29146 | GBF1 |
| ILMN_1815734 | 0.001382 | -1.71725 | FCHSD2 |
| ILMN_1719039 | 0.002792 | -1.30875 | UBE2G1 |
| ILMN_1654421 | 0.000638 | -1.53388 | MPHOSPH9 |
| ILMN_1760245 | 8.99E-05 | -1.27115 | TMEM42 |
| ILMN_1667932 | 0.001043 | -1.45907 | LOC652726 |
| ILMN_3177271 | 0.001951 | -1.25151 | LOC100129585 |
| ILMN_3300797 | 0.001534 | -1.32889 | LOC729090 |
| ILMN_1694923 | 5.54E-05 | -1.42901 | PTPN9 |
| ILMN_1847494 | 0.001036 | -1.26475 |  |
| ILMN_1704195 | 0.002026 | -1.23037 | FUK |
| ILMN_1781174 | 0.002387 | -1.38217 | KIAA1009 |
| ILMN_1785795 | 0.00215 | -1.23069 | METAP1 |
| ILMN_1829845 | 0.001324 | -1.27948 |  |
| ILMN_1691570 | 0.00012 | -1.33166 | METTL5 |
| ILMN_1801387 | 0.001232 | -1.52395 | YEATS4 |
| ILMN_2398039 | 0.001688 | -1.44832 | TCERG1 |
| ILMN_1691188 | 0.001878 | -1.24794 | UIMC1 |
| ILMN_3307877 | 2.27E-05 | -1.22539 | C21orf58 |
| ILMN_1680339 | 0.000699 | -1.28884 | PDGFRL |
| ILMN_1738099 | 0.000762 | -1.18465 | C2orf34 |
| ILMN_3282285 | 0.002178 | -1.35228 | LOC151457 |
| ILMN_2065606 | 0.000995 | -1.28984 | TOMM40L |
| ILMN_1766925 | 0.001632 | -1.27065 | CDH13 |
| ILMN_2289093 | 0.003182 | -1.32559 | KIAA1618 |
| ILMN_1675898 | 0.000283 | -1.48637 | SH3BP5 |
| ILMN_2395652 | 0.000777 | -1.38969 | PTGFR |
| ILMN_1741392 | 0.001408 | -1.28297 | SLC25A20 |
| ILMN_3237452 | 0.002578 | -1.15211 | C17orf100 |
| ILMN_1783815 | 0.000764 | -1.32787 | COG7 |
| ILMN_3236530 | 0.002926 | -1.38094 | LOC100130679 |
| ILMN_1674160 | 0.000638 | -1.14812 | BIN1 |
| ILMN_1660986 | 0.002509 | -1.34862 | PER3 |
| ILMN_1784113 | 0.002194 | -1.32007 | NAT14 |
| ILMN_1783583 | 0.001351 | -1.17314 | TMEM17 |
| ILMN_1673798 | 0.002484 | -1.26459 | PPOX |
| ILMN_1723407 | 0.001405 | -1.42865 | LOC648271 |
| ILMN_1706990 | 5.11E-05 | -1.31566 | ZNF271 |
| ILMN_1738749 | 0.000381 | -1.2642 | MAST3 |
| ILMN_3226211 | 0.003058 | -1.40978 | MUC3A |
| ILMN_1697503 | 0.001605 | -1.22907 | DHX29 |
| ILMN_1770758 | 0.001667 | -1.69825 | AKAP6 |
| ILMN_3240698 | 0.002221 | -1.93402 | LOC388279 |
| ILMN_2235785 | 1.12E-05 | -1.3363 | KCNH6 |
| ILMN_1763694 | 0.002385 | -1.12188 | RSPRY1 |
| ILMN_1652929 | 0.000799 | -1.28175 | POGZ |
| ILMN_2117809 | 0.000557 | -1.24139 | DUXAP3 |
| ILMN_2215545 | 0.001984 | -1.48704 | C3orf26 |
| ILMN_1756999 | 0.000909 | -1.33815 | RBL2 |
| ILMN_2088410 | 0.001995 | -1.24054 | PSMG2 |
| ILMN_1660199 | 0.002096 | -1.20149 | ACAA2 |
| ILMN_2053415 | 0.00085 | -1.3925 | LDLR |
| ILMN_1652749 | 0.002318 | -1.1953 | ERF |
| ILMN_1731287 | 0.000308 | -1.27675 | ARFGAP3 |
| ILMN_1676804 | 0.001688 | -1.49931 | LOC653145 |
| ILMN_3255389 | 0.003052 | -1.26718 | LOC100049716 |
| ILMN_2394571 | 0.000449 | -1.30566 | FBXW11 |
| ILMN_1771728 | 0.001355 | -1.331 | PXMP4 |
| ILMN_1709257 | 0.002764 | -1.42916 | DSCR6 |
| ILMN_2329958 | 0.000295 | -1.32521 | ABI1 |
| ILMN_1806266 | 0.000664 | -1.30007 | RAP1GDS1 |
| ILMN_1768480 | 0.000551 | -1.319 | VGLL4 |
| ILMN_3282829 | 0.00315 | -1.35882 | LOC727913 |
| ILMN_1765770 | 0.002913 | -1.28574 | SYCP2 |
| ILMN_2359014 | 0.000429 | -1.43556 | TBCE |
| ILMN_1760338 | 0.001247 | -1.0956 | LOC643357 |
| ILMN_1759154 | 0.001789 | -1.21705 | PABPN1 |
| ILMN_3298167 | 0.002074 | -1.25232 | ZSWIM7 |
| ILMN_1732575 | 0.002848 | -1.19988 | SEC14L1 |
| ILMN_2246548 | 0.000614 | -1.3346 | GSTTP2 |
| ILMN_2389347 | 0.000786 | -1.43448 | NR3C1 |
| ILMN_1780598 | 0.001917 | -1.26159 | PIAS1 |
| ILMN_1824362 | 0.000298 | -1.40064 |  |
| ILMN_1766054 | 0.000495 | -1.33999 | ABCA1 |
| ILMN_3240222 | 0.000106 | -1.27193 | PRAGMIN |
| ILMN_2180519 | 0.002319 | -1.26844 | LOC729603 |
| ILMN_3226663 | 0.000436 | -1.27575 | MGC26356 |
| ILMN_1708025 | 0.002745 | -1.72496 | RBPMS |
| ILMN_1766814 | 0.001252 | -1.28143 | TK2 |
| ILMN_1711270 | 0.000221 | -1.23389 | SFRS14 |
| ILMN_1710523 | 0.001723 | -1.49051 | ATP8B1 |
| ILMN_2399310 | 0.00161 | -1.43822 | MLLT10 |
| ILMN_1657993 | 0.001232 | -1.27496 | ADNP |
| ILMN_1808404 | 0.001151 | -1.15839 | RHBDF1 |
| ILMN_1752639 | 0.000303 | -1.27559 | SLC25A24 |
| ILMN_1657395 | 0.002496 | -1.08991 | HMGCR |
| ILMN_2219618 | 0.002178 | -1.23683 | LOC90586 |
| ILMN_1692754 | 0.000836 | -1.30355 | TMEM49 |
| ILMN_2150294 | 0.000891 | -1.21317 | FKBP14 |
| ILMN_1753500 | 0.002249 | -1.29063 | ARHGAP12 |
| ILMN_1759872 | 0.0028 | -1.14862 | LOC643509 |
| ILMN_1727574 | 0.000423 | -1.28655 | ZNF827 |
| ILMN_1723815 | 0.003242 | -1.49684 | NPEPPS |
| ILMN_2377240 | 0.000232 | -1.25537 | AKTIP |
| ILMN_1803005 | 0.002736 | -1.28662 | MMACHC |
| ILMN_2408430 | 0.00032 | -1.38384 | LARGE |
| ILMN_1755114 | 0.001552 | -1.26225 | EIF2AK4 |
| ILMN_1721344 | 0.000422 | -1.20812 | MOBKL2A |
| ILMN_3265895 | 0.003155 | -1.50158 | HNRNPR |
| ILMN_1808251 | 0.001649 | -1.4651 | C9orf38 |
| ILMN_1812312 | 0.001349 | -1.14341 | NDUFS4 |
| ILMN_1663836 | 0.002403 | -1.29753 | LOC648374 |
| ILMN_2412521 | 0.001572 | -1.09862 | KIAA0101 |
| ILMN_3260345 | 0.001706 | -1.47623 | AGFG1 |
| ILMN_1740010 | 0.002259 | -1.11084 | PCNX |
| ILMN_3187612 | 0.002884 | -1.28085 | LOC100128084 |
| ILMN_2372398 | 0.002494 | -1.28815 | ALDH5A1 |
| ILMN_2048811 | 5.86E-06 | -1.20758 | NUBPL |
| ILMN_1685097 | 0.001922 | -1.2714 | ASCC1 |
| ILMN_2131493 | 0.003051 | -1.3374 | VISA |
| ILMN_1811551 | 0.001482 | -1.20959 | DERA |
| ILMN_1768117 | 0.002743 | -1.3247 | RBM25 |
| ILMN_3261226 | 0.000865 | -1.38658 | C6orf186 |
| ILMN_1779530 | 0.002996 | -1.26578 | COG6 |
| ILMN_1722089 | 0.001398 | -1.2787 | RNF217 |
| ILMN_2043452 | 0.003155 | -1.33205 | FANCE |
| ILMN_2116127 | 0.000584 | -1.28503 | NPEPPS |
| ILMN_2315964 | 0.001753 | -1.6354 | PSRC1 |
| ILMN_1670172 | 0.000468 | -1.19937 | WDR33 |
| ILMN_1660277 | 0.001251 | -1.28687 | LOC731999 |
| ILMN_1691428 | 0.000655 | -1.22438 | PSMD12 |
| ILMN_1653133 | 0.000799 | -1.42152 | SH3D19 |
| ILMN_1716687 | 0.000278 | -1.23309 | TPM1 |
| ILMN_1784554 | 0.001742 | -1.31774 | LOC647389 |
| ILMN_2148290 | 0.002534 | -1.16267 | PDCD7 |
| ILMN_1801020 | 0.002293 | -1.40544 | ADK |
| ILMN_2335669 | 0.00084 | -1.32557 | ZC3H14 |
| ILMN_3202483 | 0.00269 | -1.17156 | LOC100133876 |
| ILMN_1704290 | 0.002814 | -1.37828 | SPTLC2 |
| ILMN_3248781 | 0.000958 | -1.15695 | SDHAP2 |
| ILMN_1683059 | 0.000715 | -1.30253 | SIRT5 |
| ILMN_1752935 | 0.001271 | -1.37259 | TMEM30B |
| ILMN_2410421 | 0.002046 | -1.23129 | NBPF1 |
| ILMN_1738491 | 0.002007 | -1.38381 | SNX30 |
| ILMN_2177090 | 0.001213 | -1.19512 | LOC200030 |
| ILMN_2340919 | 0.001081 | -1.38263 | GRB10 |
| ILMN_3235472 | 0.000609 | -1.27988 | WDYHV1 |
| ILMN_2079098 | 0.00096 | -1.25198 | C9orf80 |
| ILMN_1763326 | 0.001391 | -1.33447 | C5orf25 |
| ILMN_2055310 | 0.001046 | -1.20947 | MBD4 |
| ILMN_3294106 | 0.002004 | -1.24705 | LOC100190938 |
| ILMN_1756793 | 0.001495 | -1.38293 | POLS |
| ILMN_3241441 | 0.001254 | -1.32483 | MEGF6 |
| ILMN_1744693 | 0.002738 | -1.33146 | FGF2 |
| ILMN_1656840 | 0.001682 | -1.40093 | VPS13D |
| ILMN_2386008 | 0.000288 | -1.1523 | MPZL1 |
| ILMN_1769720 | 0.002125 | -1.35475 | STAU2 |
| ILMN_3289685 | 0.001893 | -1.2629 | LOC645452 |
| ILMN_1780444 | 0.001755 | -1.33166 | ARL3 |
| ILMN_1742827 | 0.001231 | -1.29257 | EXOC4 |
| ILMN_1756408 | 0.000435 | -1.19468 | PARVA |
| ILMN_1798581 | 0.001274 | -1.16578 | MCM8 |
| ILMN_1664440 | 0.00087 | -1.11226 | TP53BP1 |
| ILMN_1747460 | 0.002297 | -1.20181 | TMEM184B |
| ILMN_1711227 | 0.002559 | -1.22272 | GMDS |
| ILMN_1741477 | 0.001112 | -1.20017 | SMAD4 |
| ILMN_1772522 | 0.00317 | -1.25959 | ZFP161 |
| ILMN_1754234 | 0.000894 | -1.17905 | ZMYND11 |
| ILMN_2330495 | 0.001807 | -1.23977 | OCIAD1 |
| ILMN_1688452 | 0.002546 | -1.26605 | LCMT1 |
| ILMN_1671911 | 0.002033 | -1.14836 | MTA1 |
